# Rise and spread of lead-silver metallurgy deciphered from peatland archives

**DOI:** 10.1126/sciadv.aec1413

**Published:** 2026-10-07

**Authors:** Hugo Delile, André-Marie Dendievel, Laura Chollet, Iscia Codjo, Adrien Barra, Janne Blichert-Toft

**Affiliations:** ^1^ CNRS, Université Lumière Lyon 2, Archéorient-MOM, 69007 Lyon, France.; ^2^ University Claude Bernard Lyon 1, CNRS, ENTPE, UMR 5023 LEHNA, 69518 Vaulx-en-Velin, France.; ^3^ Ecole Normale Supérieure de Lyon and CNRS, 69007 Lyon, France.; ^4^ CNRS, EVS, Université Lumière Lyon 2, 69007 Lyon, France.

## Abstract

How mining has shaped long-term economic and political landscapes across Europe and the Mediterranean remains poorly understood, largely because archaeological evidence is spatially fragmented and ore sources are difficult to trace from atmospheric lead (Pb) records. Here, we compile Pb isotope data from environmental archives and introduce the Environmental Match Intensity Index (EMII), a metric applied to peat records to track Pb–silver (Ag) ore-supply patterns and relative changes in the integrated mining-related atmospheric Pb signal over six millennia. EMII reveals an early mining signal centered on Laurion (Greece) during the Late Neolithic/Chalcolithic, followed by Bronze Age expansion structured by maritime routes linking the Cyclades, the Troad, and Anatolian coasts. From the Iron Age onward, our results challenge the conventional Iberian-centered mining model and instead reveal a polycentric and multimodal organization rooted in west-central European districts interconnected through major fluvial corridors and complementary terrestrial and maritime routes. EMII reaches its first major peak during the Early Roman period, declines during Late Antiquity and the Early Middle Ages, and rises again from the High Middle Ages onward. Despite these temporal fluctuations, the overall continental distribution of the contributing mining regions remains broadly stable. By connecting environmental archives with mining, transport, and urban demand, this framework provides a scalable approach for reconstructing the long-term organization of metal production and circulation and their contribution to anthropization processes across different chrono-cultural periods and geographical settings.

## INTRODUCTION

The quest for metals has long driven human exploration, shaping long-distance trade routes, fostering colonization and imperial ambitions, and laying the foundations of premodern economic world systems (*1*). From the polymetallic copper (Cu) metallurgy of the Chalcolithic to the modern-day demand for technology-critical elements underpinning the clean energy transition, metals have remained central to societal transformation (*2*). Among historically important trace metals, none has attracted more sustained scholarly attention than lead (Pb). Beyond its intrinsic interest, Pb is most commonly associated with silver (Ag) in galena (PbS), which has historically underpinned major metallurgical economies. As such, Pb-Ag systems provide a robust proxy for tracing broader metallurgical dynamics across time and space. The widespread use and circulation of Pb, together with its chemical and isotopic properties, have provided independent evidence for the chronology and scale of past metal production, the organization of exchange networks, and the emergence and expansion of coinage in antiquity (*3*–*6*). Pb has also played a central role in forensic science, revealing modern human exposure levels far exceeding those found in uncontaminated prehistoric populations (*7*). Chronological reconstructions of Pb exposure in ancient societies (*8*–*10*), particularly in Rome, have also informed the controversial hypothesis that Pb poisoning among the Roman elite contributed to the decline of the Empire (*11*, *12*).

Palaeoenvironmental sciences have transformed our understanding of Pb’s legacy by harnessing natural archives such as peatlands, lake sediments, and polar ice cores as high-resolution proxies. These continuous records provide detailed chronologies of atmospheric Pb emissions over several millennia and document major sociopolitical disruptions, including medieval monetary reforms (*13*), disease outbreaks, and periods of territorial expansion and warfare (*14*, *15*). They also reveal changes in pollution sources, from early mining and metallurgy to the combustion of coal, residual oil, and other fossil fuels since the onset of the Industrial Revolution (*14*, *16*, *17*). A major challenge concerns the spatial scale at which mining-related atmospheric Pb emissions are recorded in environmental archives. This issue is compounded by uncertainties in the location, magnitude, and timing of ancient mining activities, as well as analytical and geological limitations, which together complicate the reconstruction of ore provenance from Pb isotope records preserved in peatlands, lake sediments, and ice cores (Fig. 1) (*18*, *19*). Some studies interpret these records as reflecting macroregional or even hemispheric atmospheric footprints, based on atmospheric transport modeling and ice-core evidence (*10*, *13*–*15*, *19*), whereas others emphasize predominantly local to regional deposition patterns, as indicated by Pb isotope fingerprints in peatlands, lakes, and sediments (*18*, *20*, *21*). This contrast has direct implications for tracing the main geographical sources of Pb-Ag ore through time. Resolving these sources would help reconstruct the long-term circulation of metallurgical knowledge, technologies, and raw materials that supported the development of interconnected economic regions across Europe and the Mediterranean.

**Fig. 1.**
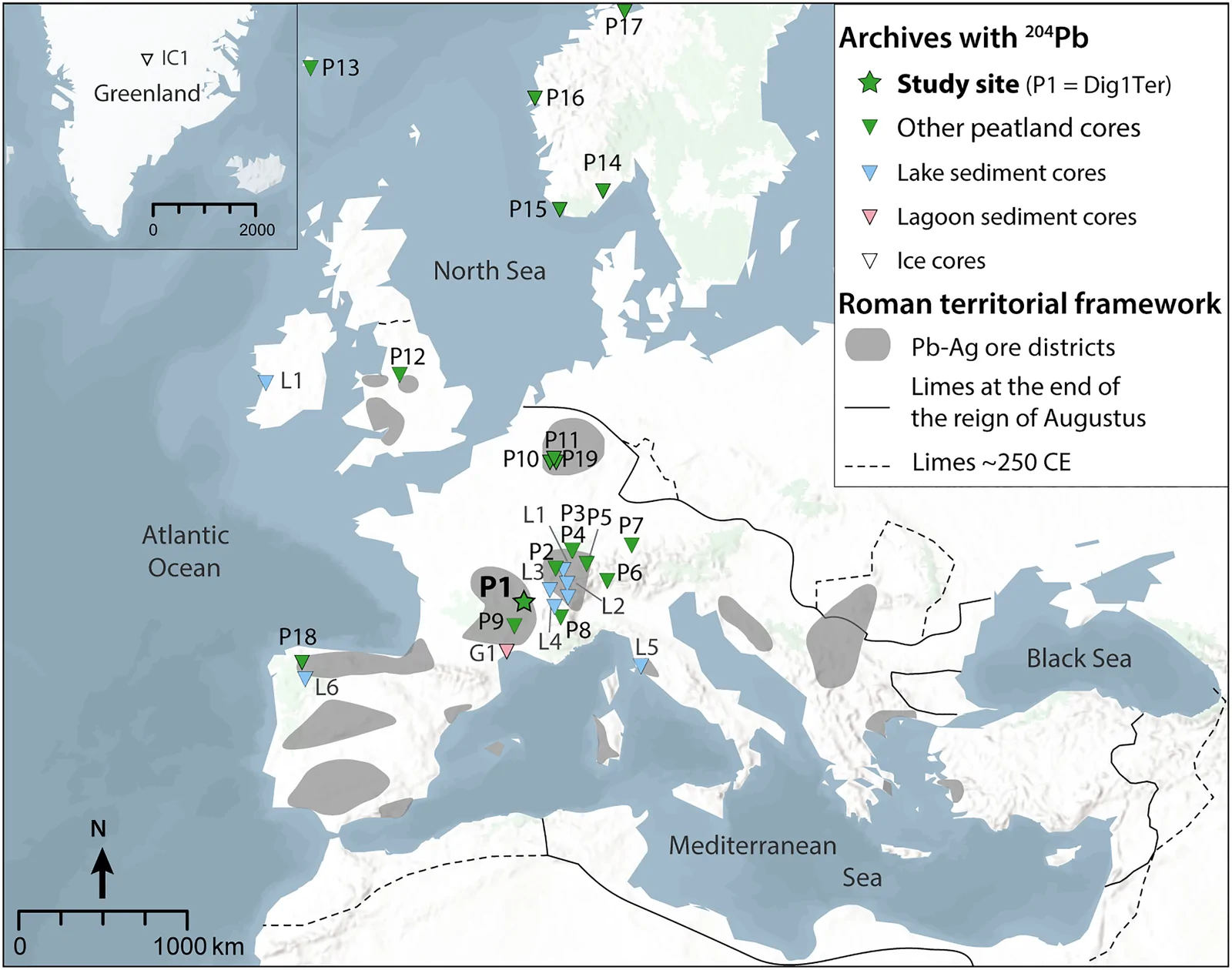
Location of atmospheric environmental archives reporting ^204^Pb-normalized isotope ratios compiled in this study. Only peatland records were retained for EMII analysis. Gray-shaded areas indicate the principal Pb-Ag mining districts exploited during the Roman period (after 62 and 126). See table S1 for detailed information on each archive.

Pb isotope analysis was first applied in the 1970s to investigate the mining origin of Ag used in Archaic and Classical Greek coinage (*22*) and was extended to environmental archives in the 1990s (*23*). Its application nevertheless remains constrained by key limitations: (i) isotopic overlap among mining regions reduces source resolution (*4*, *24*); (ii) incomplete and poorly documented Pb-Ag ore databases hinder reproducibility and transparency; (iii) potential mixing of raw materials can obscure primary isotopic signatures (*5*, *25*–*28*); and (iv) limited use of ^204^Pb-normalized isotope ratios in archaeometry and environmental sciences restricts robust source identification (*5*, *29*–*31*). These limitations have hindered the identification of the main Pb-Ag ore sources and the reconstruction of their relative importance and spatiotemporal dynamics since the emergence of metallurgy. They have consequently limited broader understanding of the role of metallurgy in the formation of early economic systems, particularly for periods that lack detailed historical documentation. In this study, we address the long-standing issue of spatial representativeness in atmospheric Pb records across Europe and the Mediterranean by compiling all available Pb isotope datasets from environmental archives that report ^204^Pb-normalized isotope ratios (Fig. 1 and table S1). We supplement this compilation with data generated in this study from a recently recovered peat core from the Pilat Massif in France (Figs. 1 and 2 and fig. S1; see also Materials and Methods) and apply a recently developed archaeometry-based algorithmic approach for Pb ore provenancing (*27*). This method stands apart from previous efforts (*32*–*34*) by accounting for the mass-dependent isotope fractionation commonly present in ore datasets and by incorporating the Lyon Pb Isotope Database, which contains ∼7000 georeferenced Pb isotope analyses of Pb-Ag ores (Fig. 3).

**Fig. 2.**
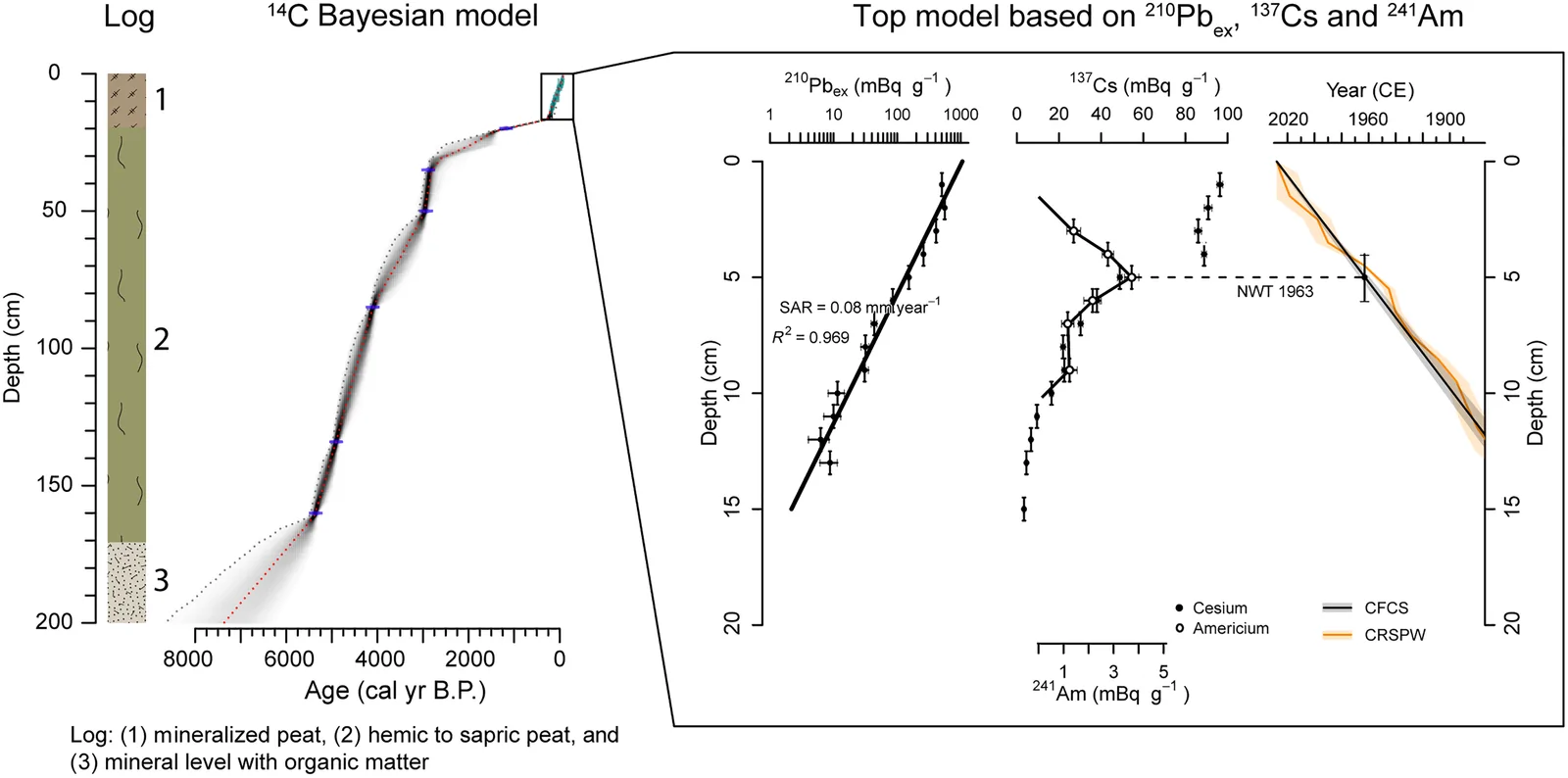
Chronostratigraphic framework of the Dig1Ter peat core. The figure shows the composite age-depth model for the Dig1Ter core recovered from the Digonnière peatland (Massif Central, France). The bottom part of the sequence was dated using a Bayesian radiocarbon age-depth model, whereas the topmost 15 cm were modeled from ^210^Pb, ^137^Cs, and ^241^Am activities using the constant rate of supply (CRS) and constant flux–constant sedimentation (CFCS) models. Also shown is the core lithology, which includes three major stratigraphic units. The resulting composite model provides a continuous chronological framework spanning the past six millennia. *r*^2^, coefficient of determination.

**Fig. 3.**
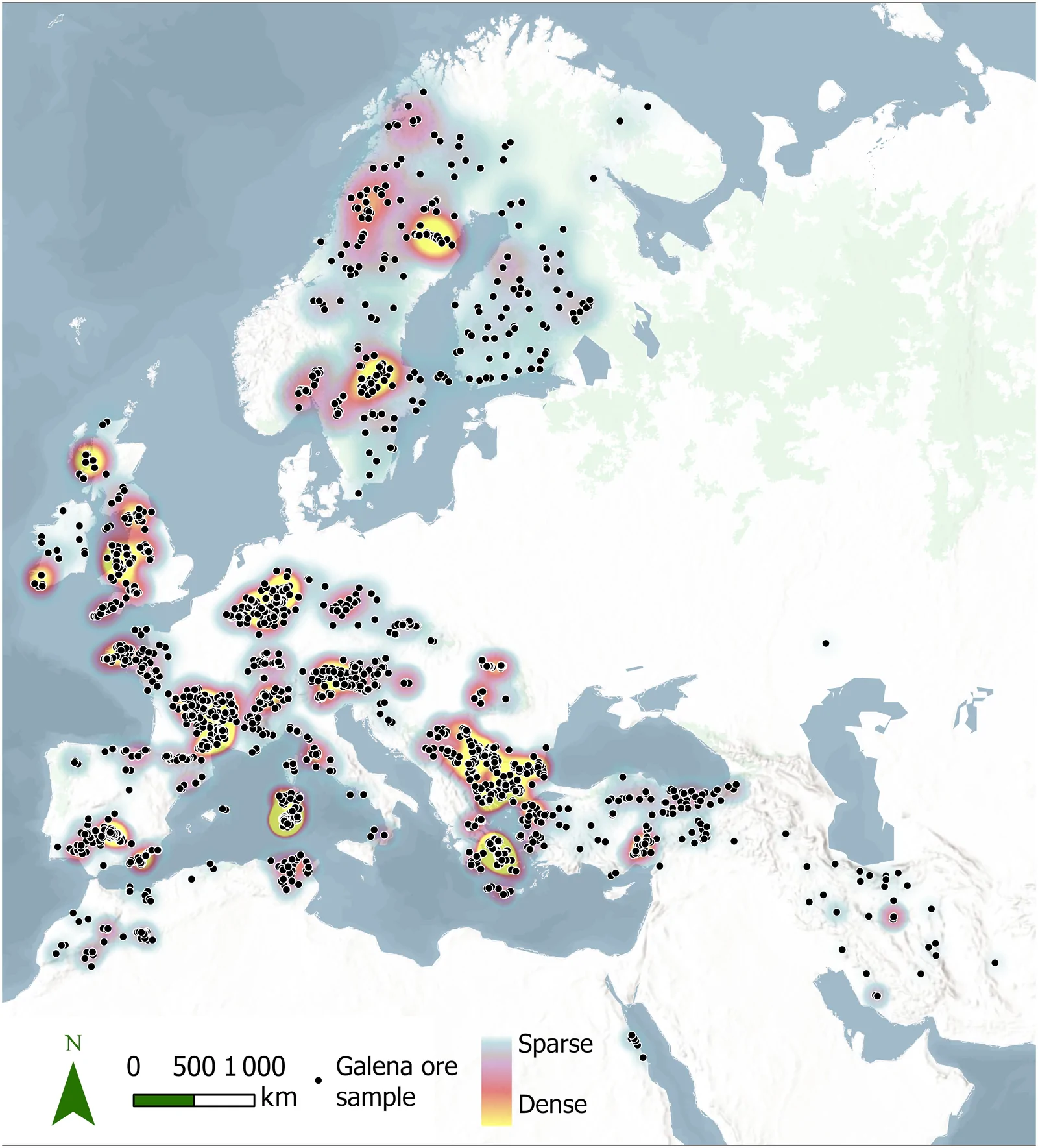
Geographic distribution of galena samples from the Lyon Pb Isotope Database (∼7000 entries) used in the EMII analysis. Black dots represent individual samples, whereas the underlying heatmap shows their spatial density and highlights the principal Pb-Ag metallogenic regions across Europe and the Mediterranean. The database represents potential ore-source fields irrespective of their period of exploitation. Data are from the Lyon Pb Isotope Database (version 2025), available through Zenodo (https://zenodo.org/records/15119912). Map image is the intellectual property of Esri and is used herein under license. Copyright 2026 Esri and its licensors. All rights reserved.

## RESULTS

### ^204^Pb isotope data in atmospheric archives

Among the nearly 100 coring sites compiled from a recent synthesis of palaeopollution records based on Pb concentrations (*19*) and supplemented with the most recent published data, approximately one quarter report measurements of ^204^Pb. This isotope, the only nonradiogenic Pb isotope, serves as the denominator in Pb isotope ratios used for source discrimination. The resulting Pb isotope database assembled from atmospheric environmental archives comprises 866 samples from 27 cores, including data from the Dig1Ter core analyzed in this study (Figs. 1 and 2, figs. S1 and S2, and tables S1 to S3; see also Materials and Methods). The three ^204^Pb-normalized isotope ratios measured in the Dig1Ter samples (^206^Pb/^204^Pb, ^207^Pb/^204^Pb, and ^208^Pb/^204^Pb) are reported in table S4. The geographical distribution among archive types is uneven: Peatlands account for slightly more than half of all entries, whereas lake sediments, ice cores, and lagoon records contribute broadly similar proportions (Fig. 4 and table S1). Based on the originally published age-depth models, each sample was assigned to 1 of the 12 chrono-cultural periods derived from established archaeological frameworks for Western Europe, from the Neolithic to the Contemporary period. Because the chronological resolution of these models varies, individual samples may represent different time spans depending on the archive and sampling strategy. Aggregation within broad chrono-cultural periods reduces the influence of this temporal heterogeneity and supports long-term comparisons but limits the resolution of finer-scale chronological dynamics.

**Fig. 4.**
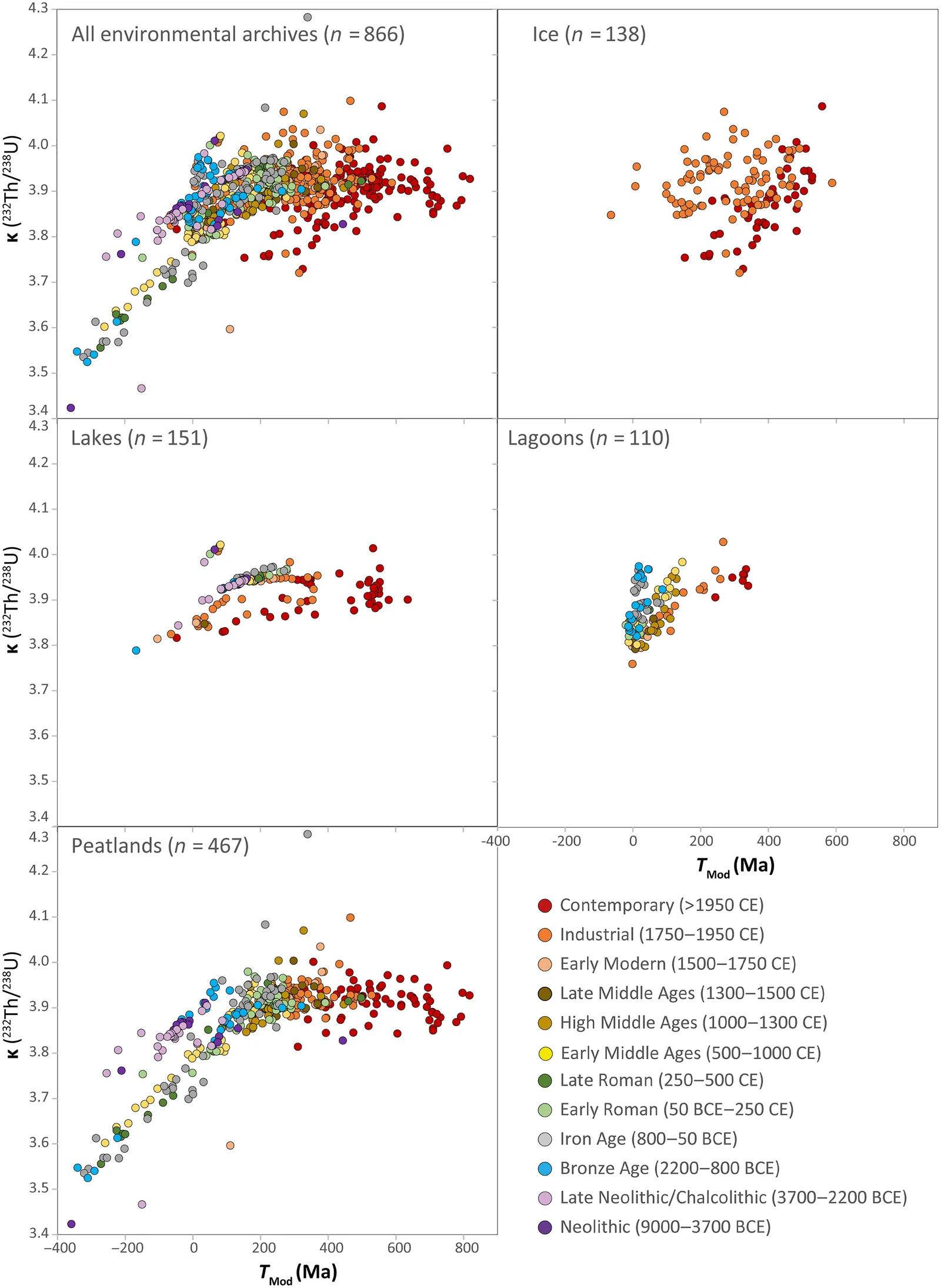
Biplots of the geochemically informed parameters *T*_mod_ (Ma) and κ (^232^Th/^238^U) for atmospheric environmental archives. The five panels show *T*_mod_ versus κ for all samples in the Pb isotope database reporting ^204^Pb-normalized ratios, first for the complete dataset and then separately for ice cores, lake sediments, lagoon deposits, and peatlands. *T*_mod_ and κ were calculated from the measured ^206^Pb/^204^Pb, ^207^Pb/^204^Pb, and ^208^Pb/^204^Pb ratios. Corresponding biplots of the measured isotope ratios are provided in figs. S3 and S4. Detailed information on each archive is given in table S1.

Pb isotope data from the environmental archives are displayed as biplots based on the geochemically informed parameters *T*_mod_, μ, and κ, calculated from the measured ^206^Pb/^204^Pb, ^207^Pb/^204^Pb, and ^208^Pb/^204^Pb ratios. *T*_mod_ is a model-derived Pb age, expressed in million years (Ma), and reflects the most recent U/Pb fractionation event in the source province. The μ (^238^U/^204^Pb) and κ (^232^Th/^238^U) parameters provide complementary information on the U/Pb and Th/U characteristics of the source reservoir. Negative *T*_mod_ values occur when Pb isotope compositions are more radiogenic than the present-day endpoint of the reference Pb-evolution model, which causes the calculated model age to project formally beyond the present. This phenomenon is known as the “future Pb paradox” (*35*). Figure 4 plots *T*_mod_ against κ to translate the measured Pb isotope ratios into geologically interpretable parameters and to reveal source-related patterns that may be difficult to distinguish in conventional isotope-ratio space. Corresponding biplots based on the three measured ^204^Pb-normalized isotope ratios are provided in figs. S3 and S4.

Ice-core Pb isotope data form a scattered cloud (Fig. 4), largely reflecting atmospheric Pb emissions over the past ∼250 years from multiple industrial sources, including coal combustion, ore smelting, and leaded gasoline, together with long-range contributions from North America and Asia and the processing of Australian Broken Hill ores in Europe (*15*, *36*, *37*). These records are therefore of limited relevance to the present study, which focuses on ore-derived Pb inputs over millennial timescales. Lake and lagoon sediment datasets are likewise dominated by postindustrial samples (Fig. 4). In addition, total digestion was used for more than 70% of lake samples and all lagoon samples; the measured Pb isotope compositions therefore combine labile Pb, primarily of anthropogenic and atmospheric origin, with residual geological Pb bound within mineral lattices. In these sedimentary archives, such mixing can make it difficult to reliably isolate the original isotopic fingerprints of mining-derived atmospheric fallout. At several sites, contributions from multiple urban sources of anthropogenic Pb also cannot be excluded.

Given the abundance of available data from peatlands, their frequent location in remote and mountain environments that limit local contamination and their representation across all 12 chronological periods, this study therefore focuses exclusively on peatlands as the most suitable archive type for tracing ore-derived Pb emissions over millennial timescales (*38*).

### Insights from a local peatland archive

Among the 467 peat samples included in the Pb isotope database of atmospheric environmental archives, 10% come from the Dig1Ter core, recently retrieved from the Pilat Massif in the French Massif Central (Fig. 1 and figs. S1 and S2). The site lies in the heart of Western Europe, within a region rich in Pb and Ag ore deposits associated with the Hercynian massifs (see Materials and Methods). Its location provides a long-term atmospheric record of mining activity from one of Europe’s most intensively exploited metallogenic regions. The chronology of the 2-m core was established using ^14^C, ^210^Pb, ^137^Cs, and ^241^Am dating. The resulting age-depth model defines a continuous sequence spanning the past six millennia (Fig. 2 and tables S2 to S4; see also Materials and Methods). Each sample was assigned to 1 of the 12 chronological periods used throughout this study: Neolithic (9000–3700 BCE), Late Neolithic/Chalcolithic (3700–2200 BCE), Bronze Age (2200–800 BCE), Iron Age (800–50 BCE), Early Roman (50 BCE–250 CE), Late Roman (250–500 CE), Early Middle Ages (500–1000 CE), High Middle Ages (1000–1300 CE), Late Middle Ages (1300–1500 CE), Early Modern (1500–1750 CE), Industrial (1750–1950 CE), and Contemporary (>1950 CE). Because no Dig1Ter samples fall within the Early Modern interval, only 11 periods are represented in this sequence, enabling comparisons from the Neolithic to the Contemporary period (Fig. 5).

**Fig. 5.**
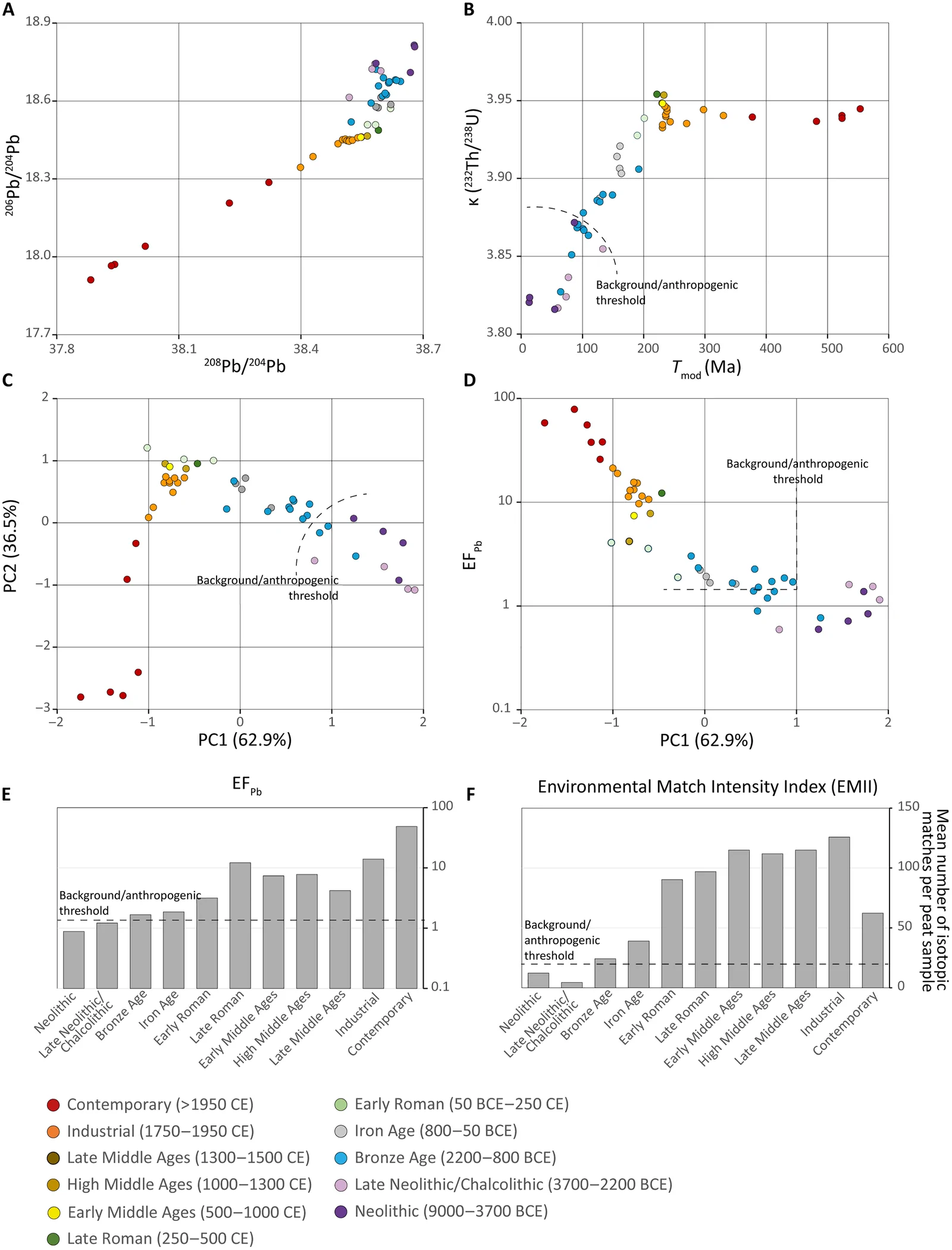
Pb isotope compositions, Pb enrichment factors, and EMII for the Dig1Ter core. (**A**) Biplot of ^208^Pb/^204^Pb versus ^206^Pb/^204^Pb; (**B**) biplot of the geochemically informed parameters *T*_mod_ (Ma) and κ (^232^Th/^238^U); (**C**) principal components analysis of Pb isotope ratios, shown as PC1 versus PC2; (**D**) relationship between PC1 and the Pb enrichment factor (EF_Pb_); (**E**) mean EF_Pb_ values for each chronological period; and (**F**) EMII, expressed as the mean number of statistically significant ore-source matches per peat sample for each chronological period represented in the Dig1Ter core (see Materials and Methods). Matches correspond to statistically significant compatibilities between the Pb isotope compositions of peat samples and galena ore-source fields represented in the Lyon Pb Isotope Database (Fig. 3).

The different representations of the Pb isotope data, including raw isotopic ratios (Fig. 5A and fig. S2), geochemically informed parameters (*T*_mod_ and κ; Fig. 5B and fig. S2), principal components analysis on ^204^Pb-normalized values (Fig. 5, C and D), and Pb enrichment factors (EF_Pb_; Fig. 5, D and E), reveal a coherent chronological trend. Natural background signatures prevail before 2200 BCE, whereas the anthropogenic imprint increases through the Bronze and Iron Ages and reaches its highest levels during the Modern and Contemporary periods. The transition from the natural background field to anthropogenically influenced Pb signatures is defined by the following thresholds: (i) *T*_mod_ > 100 Ma, (ii) κ > 3.88, (iii) PC1 < 1, (iv) PC2 > 0, and (v) EF_Pb_ > 1.5. The Pb model age threshold aligns with the well-established geological division of the European basement between Alpine and Hercynian orogenic provinces (*39*). The more radiogenic Pb isotopic compositions that characterize the natural background field agree with the global assessment of precontamination peatland records, which reports mean ratios of ^206^Pb/^204^Pb ∼ 18.90, ^207^Pb/^204^Pb ∼ 15.66, and ^208^Pb/^204^Pb ∼ 38.74 (*40*).

Figure 5F presents the Environmental Match Intensity Index (EMII), a semiquantitative index designed to track temporal changes in the mining-related atmospheric Pb signal. The index adapts a recently developed archaeometric algorithm for Pb ore provenancing (*27*) to environmental archives (see Materials and Methods). For each chronological period, EMII represents the mean number of statistically significant matches per peat sample against galena Pb isotope signatures from Pb-Ag ore deposits compiled in the Lyon Pb Isotope Database (Fig. 3). This approach enables diachronic comparisons of metallurgical activity based on isotopic density rather than binary source attribution. A statistical match is defined as the isotopic compatibility between a sample and the range of compositions expected for a given ore district, where compatibility is quantified using a chi-squared distance in a three-dimensional Pb isotope space (see Materials and Methods). EMII does not assign individual samples to unique sources but identifies recurrent compatibilities across the dataset. The index thus provides a relative, semiquantitative measure of temporal changes in the integrated mining-related atmospheric Pb signal and supports spatiotemporal comparisons of provenance patterns (see Materials and Methods). To ensure that isotopic comparisons primarily reflect mining-derived inputs rather than natural background, the reference dataset was restricted to galena (PbS) ores (Fig. 3), which represent the main carrier phase of Pb in ore deposits.

Because peatland archives integrate emissions from several contemporaneous sources, individual samples commonly record mixed contributions from multiple ore bodies. In Pb isotope space, these mixtures plot along mixing arrays between end-members rather than coinciding with a single ore signature (*27*). Where atmospheric Pb inputs are dominated by emissions associated with a single isotopically coherent ore-source field, the mixed composition remains close to that field and yields a high density of statistically significant matches, while contributions from other potential ore sources remain weakly represented. Where atmospheric Pb inputs reflect more balanced contributions from several potential ore sources, samples plot in intermediate regions of isotope space that may not correspond to any single ore-source field in the reference database, and statistically significant matches become more diffuse across multiple potential sources. This behavior is consistent with Pb isotope studies showing that mixtures from multiple sources generate compositional fields that may not coincide with individual ore-source signatures and can remain partly unresolved (*6*, *27*, *28*). EMII accommodates such configurations by quantifying recurrent statistically significant matches across the dataset, thereby highlighting the metallogenic regions most consistently represented despite mixed inputs.

In the Dig1Ter core, EMII is calculated using only samples from the peat sequence and summarized across the 11 chronological periods represented in this archive, from the Neolithic to the Contemporary period (Fig. 5F). Its behavior is compared with independent geochemical proxies, such as EF_Pb_, within the well-constrained local stratigraphy (Fig. 2). EMII closely follows EF_Pb_ and the other Pb isotope indicators (Fig. 5, A to E). Values remain very low (<12) during the Neolithic and Late Neolithic/Chalcolithic periods, which indicates a limited mining-related atmospheric Pb signal. They increase moderately during the Bronze and Iron Ages (>24) and then rise sharply from the Roman period onward, approaching 100 during the Roman phase and exceeding 100 from the Early Middle Ages onward. In contrast to EF_Pb_, which reaches its maximum during the Contemporary period, EMII declines by nearly half (Fig. 5, E and F). This divergence reflects the increasingly mixed and globalized origin of atmospheric Pb over the past ∼250 years, including long-range contributions from North America and Asia and the processing of Australian Broken Hill ores in Europe (*15*, *36*, *37*).

## DISCUSSION

Having demonstrated the consistency of EMII with independent geochemical proxies in the well-constrained Dig1Ter peatland archive (Fig. 5), the same framework is applied to the European peatland network compiled from the literature (Fig. 1 and table S1) to reconstruct continental-scale patterns through time (Fig. 6). For the European compilation, EMII is summarized across 12 chrono-cultural periods to resolve major historical transitions, including subdivisions of the Roman and medieval periods (Fig. 6). The geovisualizations are organized into three groups: (i) the Late Neolithic/Chalcolithic, Bronze Age, and Iron Age (Fig. 7); (ii) the Roman period and Middle Ages (Fig. 8); and (iii) the Early Modern, Industrial, and Contemporary periods (Fig. 9). Additional figures in the Supplementary Materials provide finer temporal resolution by subdividing the Roman period into Early and Late Roman phases (fig. S5) and the medieval period into the Early, High, and Late Middle Ages (fig. S6).

**Fig. 6.**
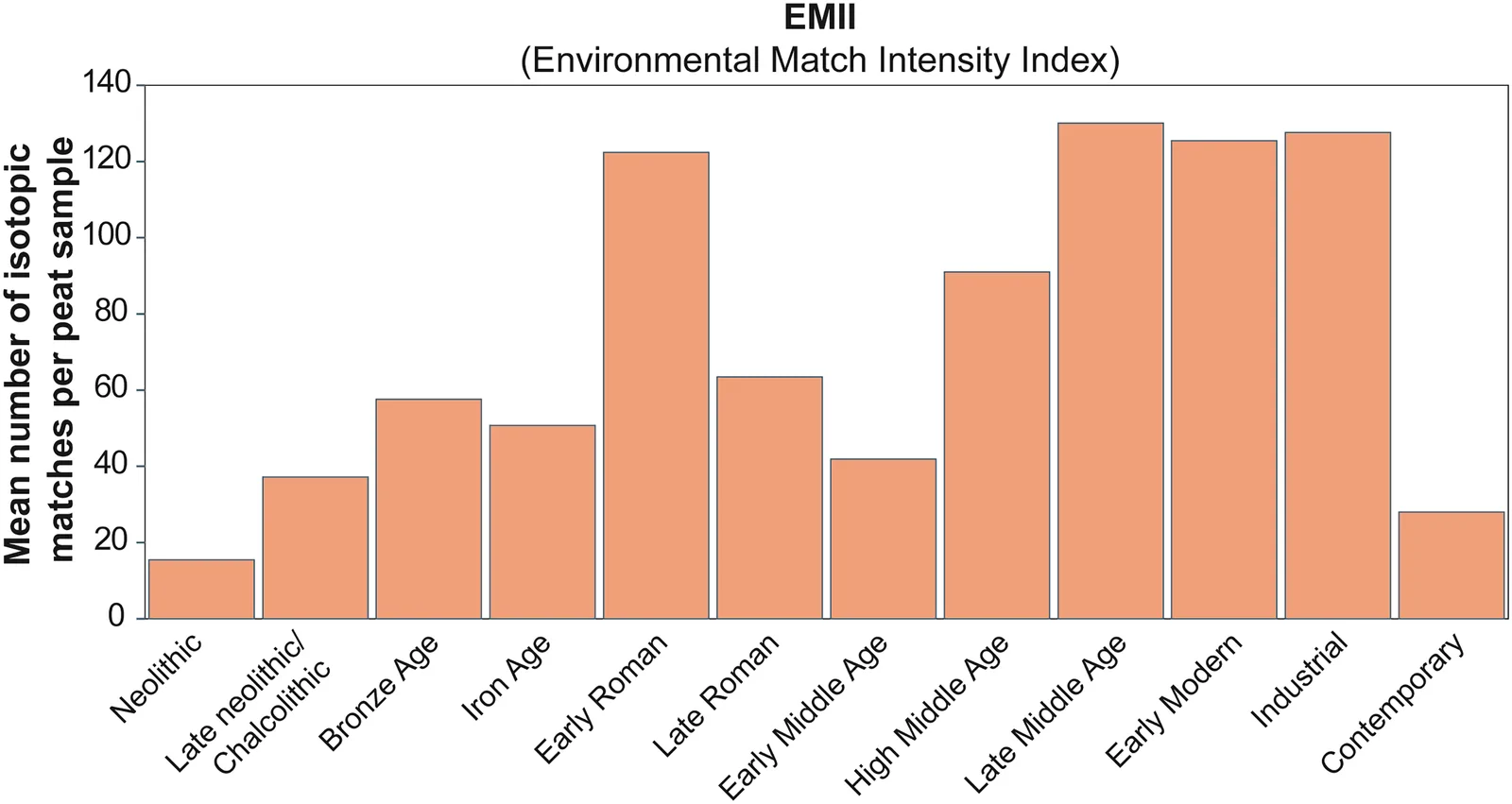
EMII for the compiled network of 19 peatland records. For each chronological period, EMII represents the mean number of statistically significant ore-source matches per peat sample between peat-derived Pb isotope compositions and galena signatures from Pb-Ag deposits represented in the Lyon Pb Isotope Database. EMII provides a semiquantitative proxy for the intensity of mining-derived atmospheric Pb inputs through time. The chronological periods are defined as follows: Neolithic (9000–3700 BCE), Late Neolithic/Chalcolithic (3700–2200 BCE), Bronze Age (2200–800 BCE), Iron Age (800–50 BCE), Early Roman (50 BCE–250 CE), Late Roman (250–500 CE), Early Middle Ages (500–1000 CE), High Middle Ages (1000–1300 CE), Late Middle Ages (1300–1500 CE), Early Modern (1500–1750 CE), Industrial (1750–1950 CE), and Contemporary (>1950 CE).

**Fig. 7.**
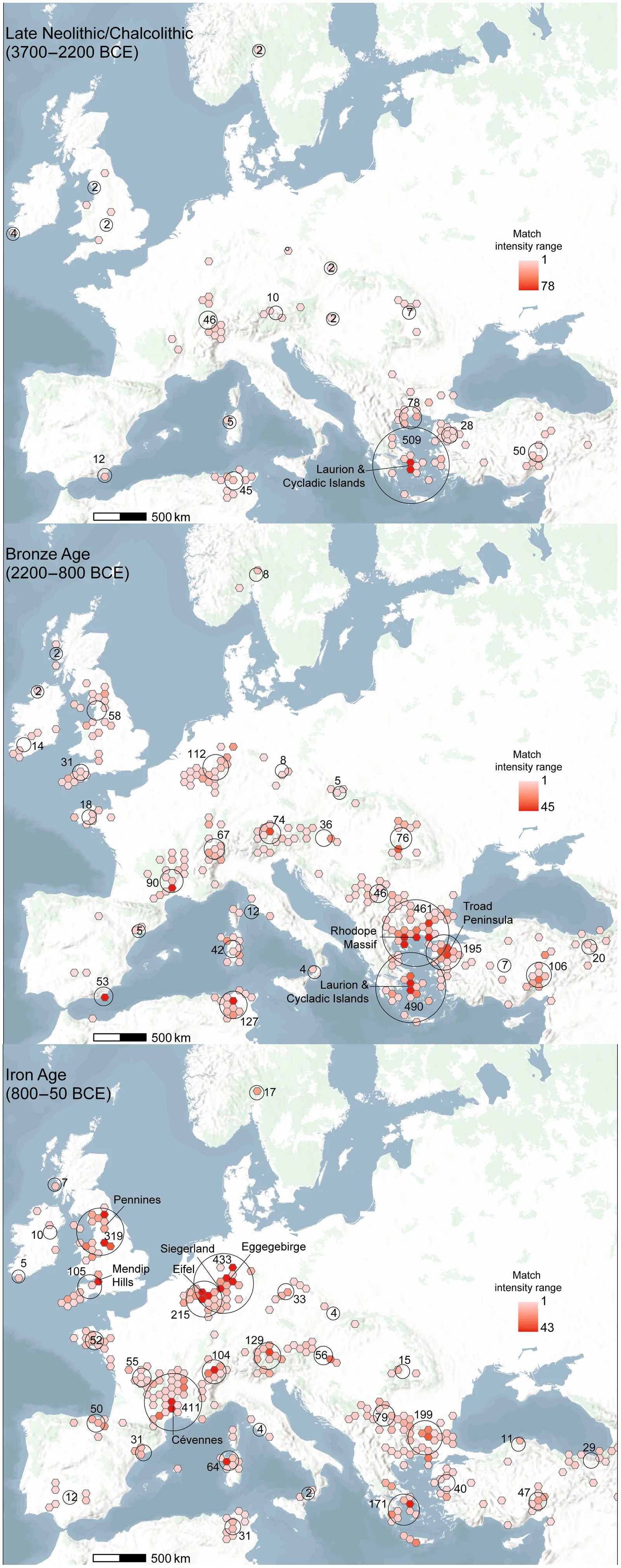
Spatial distribution of potential Pb-Ag source regions inferred from EMII matches across Europe and the Mediterranean during the Late Neolithic/Chalcolithic, Bronze Age, and Iron Age. For each chronological period, Pb isotope compositions measured in the 19 peatland records (Fig. 1 and table S1) were compared with galena Pb isotope signatures from the Lyon Pb Isotope Database (Fig. 3) using the EMII. Color-coded hexagonal cells, each covering 4000 km^2^, show the number of isotopic matches within each cell. Proportional circles generated by spatial clustering represent the total number of matches at each aggregated location, with numerical labels indicating the corresponding counts. Hexagonal cells with the highest match densities are labeled with the names of the corresponding Pb-Ag ore-bearing regions. The mapped areas represent potential source regions inferred from isotopic compatibility, irrespective of their documented period of exploitation. Map image is the intellectual property of Esri and is used herein under license. Copyright 2026 Esri and its licensors. All rights reserved.

**Fig. 8.**
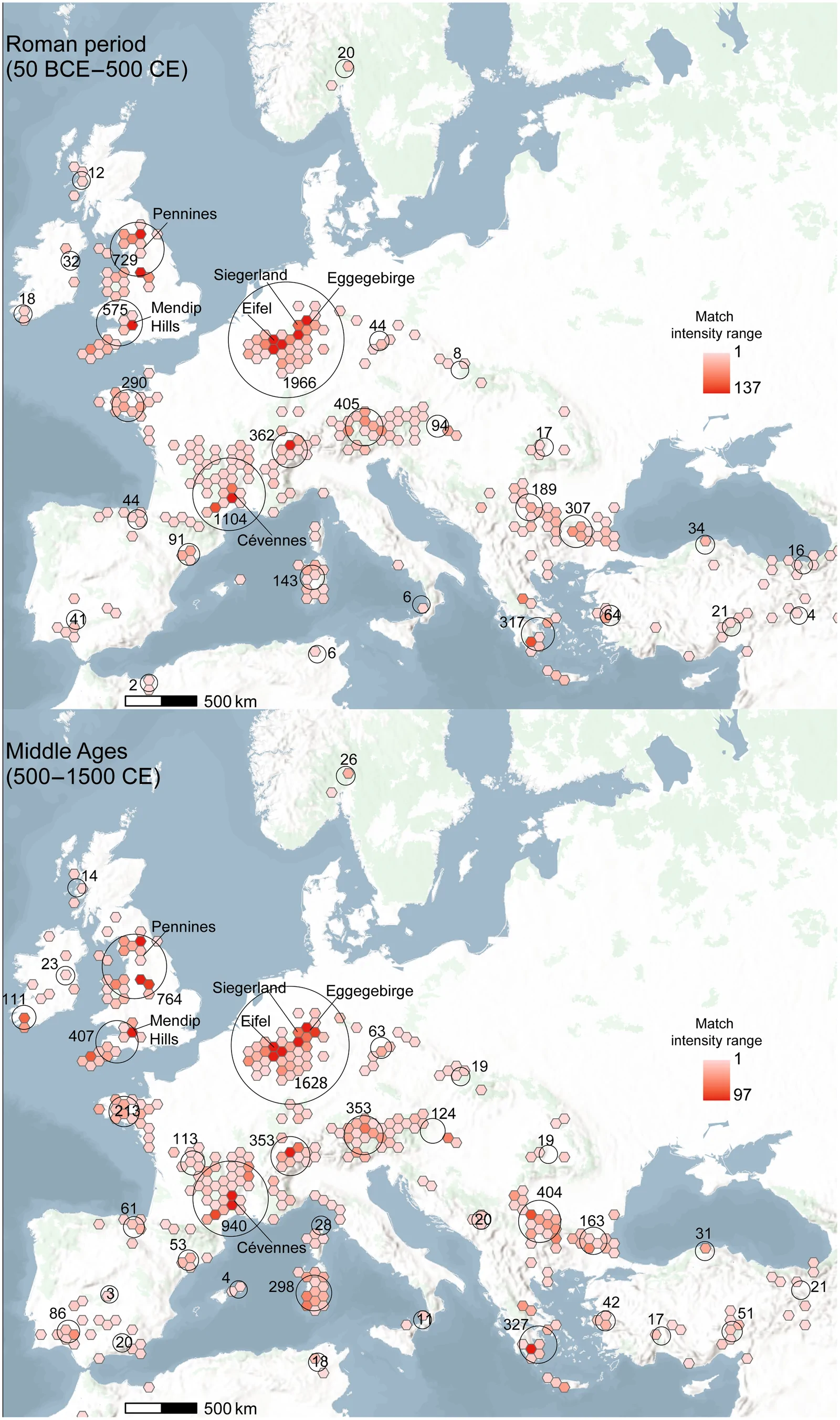
Spatial distribution of potential Pb-Ag source regions inferred from EMII matches across Europe and the Mediterranean during the Roman and medieval periods. For each chronological period, Pb isotope compositions measured in the 19 peatland records (Fig. 1 and table S1) were compared with galena Pb isotope signatures from the Lyon Pb Isotope Database (Fig. 3) using the EMII. Color-coded hexagonal cells, each covering 4000 km^2^, show the number of isotopic matches within each cell. Proportional circles generated by spatial clustering represent the total number of matches at each aggregated location, with numerical labels indicating the corresponding counts. Hexagonal cells with the highest match densities are labeled with the names of the corresponding Pb-Ag ore-bearing regions. The mapped areas represent potential source regions inferred from isotopic compatibility, irrespective of their documented period of exploitation. Map image is the intellectual property of Esri and is used herein under license. Copyright 2026 Esri and its licensors. All rights reserved.

**Fig. 9.**
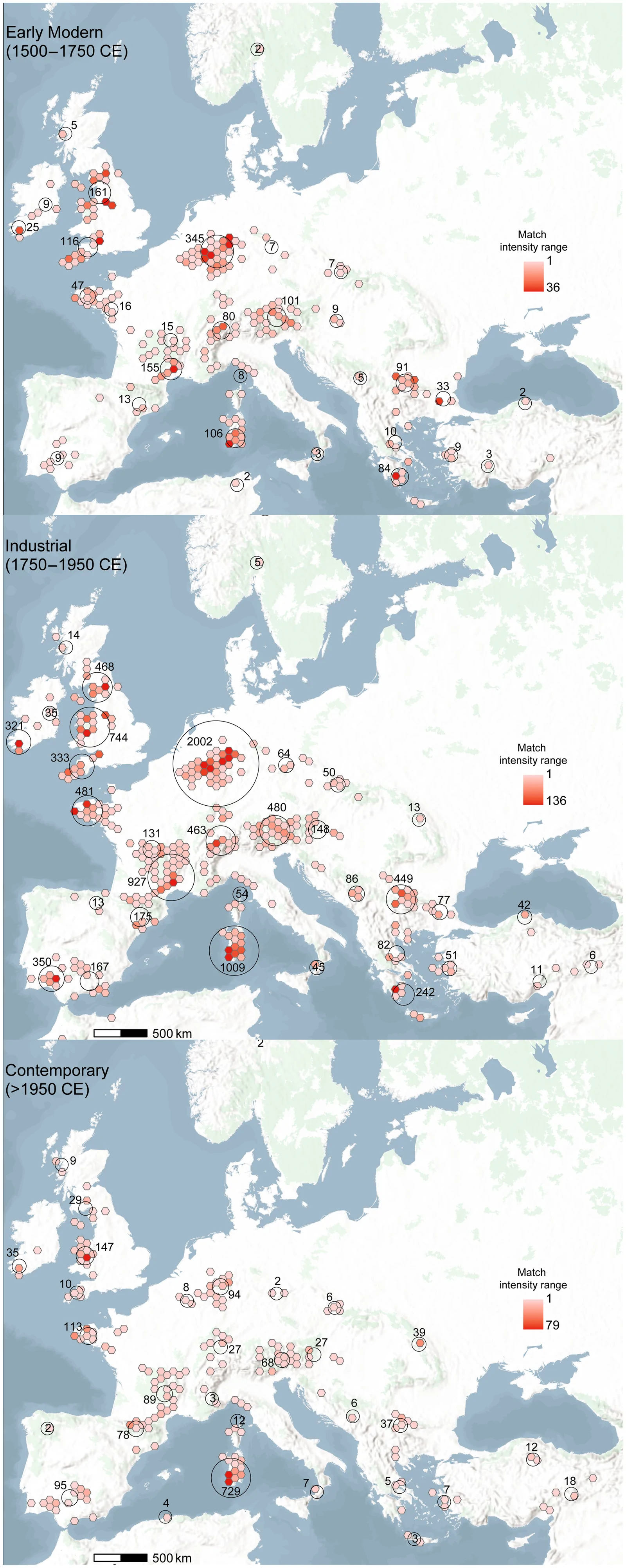
Spatial distribution of potential Pb-Ag source regions inferred from EMII matches across Europe and the Mediterranean during the Early Modern, Industrial, and Contemporary periods. For each chronological period, Pb isotope compositions measured in the 19 peatland records (Fig. 1 and table S1) were compared with galena Pb isotope signatures from the Lyon Pb Isotope Database (Fig. 3) using the EMII. Color-coded hexagonal cells, each covering 4000 km^2^, show the number of isotopic matches within each cell. Proportional circles generated by spatial clustering represent the total number of matches at each aggregated location, with numerical labels indicating the corresponding counts. The mapped areas represent potential source regions inferred from isotopic compatibility, irrespective of their documented period of exploitation. Map image is the intellectual property of Esri and is used herein under license. Copyright 2026 Esri and its licensors. All rights reserved.

### Emergence and organization of early metallurgical systems (Neolithic to Iron Age)

#### Late Neolithic/Chalcolithic (3700–2200 BCE)

Applied to the peatland subset of the Pb isotope database compiled from atmospheric environmental archives, EMII increases from ∼15 during the Neolithic to ∼37 during the Late Neolithic/Chalcolithic (Fig. 6), indicating the emergence of a broader mining-related atmospheric Pb signal consistent with increasing metallurgical activity. This trend closely mirrors the earliest evidence of atmospheric Pb pollution, previously recorded as localized and spatially confined traces in peatlands from southeastern Europe around 3600 BCE (*41*) and southwestern Europe by 3000 BCE (*42*). EMII geovisualization reveals a marked concentration of isotopic matches in the Aegean region (Greece), particularly around the Laurion mining district in Attica (Greece) and the Cycladic islands of Serifos, Kythnos, and Kea (Fig. 7), which together account for more than 60% of the matches. Laurion was exploited for its Cu and Pb, but primarily for its Ag, with galena serving as the principal Ag-bearing mineral and an important source of Pb. This early atmospheric pollution signal is particularly noteworthy because it coincides with archaeological evidence for the earliest mining activities at Laurion during the late fourth to early third millennium BCE (*43*). These results underscore Laurion’s prominence during the earliest phases of Ag metallurgy. Archaeological evidence further indicates that extraction initially focused on surface and near-surface mineralization through small-scale and spatially dispersed operations, before shifting during the third millennium BCE toward shallow underground workings and the systematic pursuit of ore veins (*44*–*47*). This development reflects an early intensification and organization of extraction systems, consistent with the increase in EMII observed during the Late Neolithic/Chalcolithic (Fig. 6).

#### Bronze Age (2200–800 BCE)

The Bronze Age is marked by a further rise in EMII to ∼58 (Fig. 6), reflecting intensified Pb and Ag mining and smelting activities. This continental-scale increase echoes sporadic, but convergent, local signals from Western Europe, including the Dig1Ter core analyzed in this study, as well as from central Europe (*48*), southwestern Europe (*42*), and southeastern Europe (*41*). The geovisualization of EMII results for the Bronze Age reveals a coherent and spatially extensive pattern of potential Pb-Ag source regions across the Aegean and surrounding areas, extending from Laurion and the Cyclades (Serifos, Kythnos, Siphnos, and Kea) through the Rhodope Massif (Bulgaria-Greece; e.g., northeastern Chalkidiki, Thasos, Kirki-Sappes-Kassiteres, Evros, and Madzharovo) to the Troad Peninsula (Türkiye) (Fig. 7). This spatial pattern points to a predominantly eastern metallurgical system centered on the Aegean and structured by maritime exchange routes. It was likely supported by Cycladic societies, whose insular networks fostered early trade connections across the Aegean. Archaeological and textual evidence likewise indicates maritime routes linking the Cyclades to the Troad and Anatolian coasts (*49*), forming a backbone for the circulation of metals and technological know-how. Although archaeological research has long identified key sites such as Siphnos, Laurion, and Asia Minor as major sources of Ag (*50*), the wider geographical consistency of such activity has remained difficult to assess, owing to the local and discontinuous nature of excavation data. In contrast, the present geochemical signal provides a macroregional perspective that brings these site-specific observations together within a broader picture of Bronze Age metallurgical expansion. Together, these data point to the coordinated development of metal procurement and the diffusion of technological expertise across a vast, interconnected maritime exchange network.

#### *Iron Age (800*–*50 BCE)*

The transition from the Late Bronze Age to the Early Iron Age (12th to 8th centuries BCE) marks a turning point in European metallurgical history. During this interval, long-distance exchange networks between the eastern and western Mediterranean expanded, notably through the Phoenicians’ early exploitation of Ag-rich galena deposits in Iberia to supply eastern Mediterranean markets (*5*, *14*, *25*, *51*). EMII decreases slightly to ∼51 during the Iron Age (Fig. 6). This modest decline coincides with a period of sociopolitical fragmentation among the complex societies of the eastern Mediterranean. At the same time, EMII-based geospatial patterns challenge the conventional Ibero-centric mining model by highlighting substantial contributions from central and Western European ore districts, including the French Massif Central (Cévennes), the Rhenish Massif (Siegerland and Eifel), the German Central Uplands (Eggegebirge), and southern Britain (Pennines and Mendip Hills) (Fig. 7). This distribution reveals a shift toward an inland-centered metallurgical organization structured along major fluvial corridors and complemented by maritime connections. Iron Age metal-supply networks therefore appear to have been more geographically diversified than previously assumed, with west-central European sources contributing substantially to wider Mediterranean exchange systems. This shift coincides with the rise of Late Hallstatt and La Tène Celtic societies, which progressively developed proto-urban settlements and communication routes along the Rhône-Saône fluvial corridor (*52*). Archaeological evidence indicates that, during this period, trade routes combined terrestrial and fluvial segments and connected the Rhine, Moselle, and Meuse basins to the Rhône through Besançon and Basel (*52*). These northern corridors were closely connected to mining districts in the Rhenish Massif. Classical sources further attest to Pb and Ag production in southern Gaul during the late Iron Age, notably among the *Ruteni* and *Gabalies*, as reported by Strabo (Geography III.2.8; IV.1.12; IV.2.2). The upland mining districts of the Cévennes and Mont Lozère, located close to the Rhône valley, were already integrated into exchange networks linking them to emerging southern urban centers via terrestrial routes along the eastern margin of the Massif Central and the Rhône corridor (*52*).

These connections helped integrate inland resource zones into Mediterranean exchange networks and connected small fortified Celtic sites, some of which functioned as emporia, with Greek colonies established along the French Mediterranean coast, including Marseille, Béziers, Agde, and Olbia (*53*). The expansion of these exchange networks is also reflected in the growing use of Ag coinage. Greek monetary systems first introduced drachmas and obols during the fourth century BCE, while local production developed in southern Gaul from the late third century BCE and intensified markedly during the second and first centuries BCE (*54*). A recent synthesis identifies particularly high densities of Ag coinage in the Cévennes and the upper Saône and Rhône basins (*55*), which suggests close connections between Pb-Ag production areas and major exchange networks across the Massif Central and neighboring regions. Rather than reflecting purely local production, these patterns underscore the integration of mining districts within broader circulation systems. This configuration reveals a coevolution between expanding trade and communication corridors and major mining districts during the Iron Age. Western European metallurgy therefore appears to have developed through regional exchange networks, particularly along the transalpine and Rhône-Saône axes, rather than being driven primarily by eastern Mediterranean colonial dynamics, as traditionally proposed (*14*, *25*, *51*).

### Roman peak and large-scale integration

Unlike earlier periods, the Roman and medieval phases are documented by extensive archaeological, archaeometric, and textual evidence. EMII should therefore be considered a complementary proxy that reflects the integrated atmospheric signal of metallurgical activity across several regions. Differences between EMII results and conventional archaeological syntheses may thus arise from the different nature and spatial scale of the evidence on which they rely.

#### Pb and Ag mining output from the Early to Late Roman period

The transition to the Roman period marks a major inflection point in the EMII trajectory. Values reach a maximum of ∼122 during the Early Roman period before declining to ∼63 in the Late Roman phase (Fig. 6). This Early Roman peak reflects an unprecedented intensification of Pb and Ag mining and smelting activities, in agreement with numerous environmental records (*19*). Greenland ice cores likewise record exceptionally high Pb emissions during the *Pax Romana*, when imperial consolidation and sustained economic expansion reached their height (*14*). Both EMII and independent ice-core records show a marked decline during the Late Roman period. EMII decreases by approximately a factor of 2 (Fig. 6), whereas Pb concentrations and depositional fluxes in Greenland ice fall by approximately a factor of 3 (*10*, *14*). Archaeological and numismatic evidence suggests that the contraction of Roman Pb-Ag production after the second century CE was neither uniform nor abrupt. Rather, it reflected the gradual weakening of large, centrally controlled mining systems amid the political and economic disruptions of the third century CE (*56*, *57*), together with the absence of major new mines capable of replenishing precious-metal supplies (*58*). Mining nevertheless persisted into Late Antiquity, although in more localized and less intensive forms (*56*, *59*). The resulting contraction probably deepened the imbalance between metal supply and monetary demand during the third-century crisis (*57*, *58*). Major epidemic outbreaks further aggravated these pressures by reducing labor availability and disrupting circulation networks (*14*, *60*).

The apparent reduction in Pb and Ag production between the Early and Late Roman periods (Fig. 6) is not accompanied by a reorganization of supply networks in the EMII-derived geospatial patterns. The main mining districts contributing to the atmospheric signal throughout the Roman period (Fig. 8) remain similar to those already active during the Iron Age, namely, the French Massif Central, the Rhenish Massif, the German Central Uplands, and southern Britain (Fig. 7). No marked spatial change is evident between the Early and Late Roman phases (fig. S5). This stability differs from the pattern commonly emphasized in archaeological syntheses, which identify Roman Hispania as the primary region of Pb production and export within the Empire, complemented by secondary districts in Britain and Germania after imperial expansion and the later development of mining regions in the Balkans, where exploitation appears to have continued longer than in Western Europe (*61*–*66*). This divergence does not necessarily indicate a contradiction but more likely reflects the different evidence represented by each approach. Archaeological and archaeometric studies document the location, chronology, and relative importance of individual mining districts, particularly in Hispania, whereas EMII records an integrated atmospheric signal from several contemporaneous sources, weighted by both emission intensity and long-range transport. The persistence of a broadly stable EMII spatial pattern throughout the Roman period does not imply that the location, intensity, or organization of mining activity within these regions remained unchanged. Rather, it indicates that the atmospheric Pb signal continued to bear the imprint of the same broad set of metallogenic provinces, without the emergence of new large-scale centers capable of reshaping its overall geography. This interpretation nevertheless requires caution because the temporal resolution of EMII remains limited to broad chronological periods. Identifying finer-scale reorganizations, including possible temporal offsets between western and Balkan mining systems, will require denser and more evenly distributed isotopic datasets for each time interval.

#### Reassessing the conventional Ibero-centric Roman mining model

At the scale of the Roman period, the EMII-derived geospatial patterns show that regional Pb-producing districts already active during the Iron Age continued to contribute to the atmospheric signal (Fig. 8), whereas Iberian sources remain weakly represented, particularly the southeastern districts of Cartagena-Mazarrón and the southwestern districts of the Sierra Morena. This limited Iberian contribution persists despite the inclusion of a Galician peat archive (Fig. 1), which accounts for ∼20% of the dataset and lies close to the Basque-Cantabrian metallogenic province. However, its location primarily constrains inputs from northern Iberia and does not necessarily provide equivalent sensitivity to emissions from southern districts. Atmospheric circulation may also have reduced their representation. Over Europe, prevailing westerlies, modulated by large-scale pressure systems such as the Azores High and Icelandic Low, generally favor northwest-to-southeast transport pathways and may therefore limit the northward and eastward dispersal of Pb aerosols released from southwestern Iberia [e.g., (*67*, *68*)]. However, atmospheric circulation should be considered a modulating influence rather than a sufficient explanation. The isotopic basis of EMII and its ability to identify matches over distances greater than ∼2500 km, including contributions from eastern Mediterranean sources (Fig. 7), indicate that circulation patterns alone cannot explain the observed spatial distribution. These results nevertheless diverge from the dominant archaeological view, which identifies Roman Hispania as the principal region of Pb production and export within the Empire, alongside secondary districts in Britain and Germania (*61*–*65*, *69*). This divergence does not call into question the importance of Iberian mining during the Roman period, which is extensively documented by mining remains, archaeological excavations, and archaeometric analyses. In this respect, the recent synthesis by Domergue and Rico (*64*) provides the most comprehensive account now available for any ancient mining region, based on an exceptionally rich corpus of archaeological, epigraphic, archaeometric, and textual evidence. The prominence accorded to Hispania also reflects the historiographical foundations of mining archaeology. From its inception, the discipline has drawn heavily on classical authors such as Polybius (X.38.7; XXXIV.9.8, as cited in Strabo III.2.10), Posidonius (as cited in Strabo III.2.9), Strabo (III.2.9–10), and Diodorus Siculus (V.35–38), whose emphasis on the mineral wealth of Hispania strongly influenced subsequent archaeological investigations (*61*, *63*). This historiographical legacy helped establish a conventional model of Roman mining centered on the primacy of Iberian production, while other mining regions received less systematic attention.

A well-documented case illustrates how interpretations of Roman metal supply have changed as additional datasets became available and earlier research biases were reassessed. Pb ingots recovered from shipwrecks at Saintes-Maries-de-la-Mer (France) were initially attributed to Iberian mining districts (*70*), in accordance with the prevailing view of Hispania as the principal source of Roman Pb. This attribution was later revised in favor of southern Gaul, particularly the Cévennes (*71*). More recent isotopic analyses, made possible by the expansion of Pb isotope reference datasets for European ore deposits, have instead linked the ingots to the Rhenish Massif and neighboring German mining districts (*71*, *72*). This progressive reassessment highlights the extent to which provenance studies depend on the availability and representativeness of Pb isotopic reference databases from Pb-Ag ore deposits and reveals the role of research bias in shaping current understanding of ancient production systems. It also suggests that continental mining regions were not marginal to Roman metal supply, but rather insufficiently identified within earlier analytical frameworks.

Within the Rhenish Massif, the main EMII clusters are concentrated in the Eifel and Siegerland regions, with a further extension toward the Eggegebirge, near the Sauerland mining district documented in the literature (Fig. 8). These clusters are consistent with isotopic and archaeological evidence for Roman Pb production in the northern Rhenish Massif and neighboring German districts (*73*). They also coincide with long-recognized ancient mining districts on both sides of the Rhine River (*62*, *63*, *65*), whose contribution to Roman metal supply may have been underestimated in broader syntheses. A similar correspondence emerges in the southern Massif Central, where a pronounced EMII signal identifies the Cévennes as a major potential Pb-Ag production center (Fig. 8). This location accords with archaeological syntheses (*62*), isotopic evidence linking Ag treasures to the Cévennes–Mont Lozère area (*74*), and broader isotopic studies that identify the Massif Central (*75*) and southern Gaul (*76*) as potential sources of Roman Ag. Environmental records from Mont Lozère provide further support, with evidence of metallurgical activity from the Late Iron Age to the Early Roman period (*65*, *77*). Together, these data confirm the southern Massif Central as an important component of the western Roman metal-supply system. In *Britannia*, EMII clusters clearly delineate the Pennine and Mendip regions (Fig. 8). These clusters coincide with two of the principal Pb-Ag mining districts identified in archaeological syntheses, which document a series of mining areas extending from the Mendip Hills in southwestern England, near the Severn estuary, northward through the Welsh borderlands of Shropshire and Flintshire, across the central Peak District in Derbyshire, and into the Pennines in Yorkshire (*62*, *63*, *65*, *69*). The strong EMII signals in both the Pennine and Mendip regions indicate that these districts were already active during the Late Iron Age, before the Roman conquest in the first century CE. Their continued prominence throughout the Roman period suggests that imperial control incorporated and intensified pre-existing production systems.

#### Metal flows across a polycentric and multimodal backbone of fluvial corridors

Roman metal circulation has traditionally been framed within a dominant south-to-north model rooted in the conventional view of Roman mining. Based largely on epigraphic and isotopic studies of Pb ingots recovered from Mediterranean shipwrecks, this framework has supported the reconstruction of several major circulation routes (*63*, *64*). One principal route carried Iberian Pb and Ag by sea to the major ports of southern Gaul, particularly *Narbo Martius* (Narbonne, France) and *Arelate* (Arles, France). From these ports, metals were transported toward the northern provinces and the Rhine frontier to supply military zones in the northwestern Empire. In line with the presence of northern districts in Britain and the Germanic regions, traditionally regarded as peripheral, secondary north-to-south circulation routes have been proposed. These routes connected northwestern Europe with the Mediterranean through maritime, fluvial, and terrestrial transport, notably via the English Channel, the Seine basin, and the Rhône-Saône corridor (*63*, *64*, *77*–*79*).

The EMII geovisualization results (Fig. 8) suggest that the relative importance of these circulation patterns should be reassessed, as northern supply routes appear more prominent than previously assumed. Finds of British and German Pb ingots along the Channel coast, in the Seine estuary, along the Saône corridor, in the Rhône delta, and across Mediterranean maritime routes provide direct material evidence of substantial north-to-south metal flows (*79*). Roman metal supply therefore did not represent a new organization but rather extended a polycentric and multimodal system inherited from the Iron Age. This system relied on continental corridors and incorporated maritime connections from *Britannia*. This interpretation aligns with models of Roman economic organization that emphasize hierarchical and multimodal transport systems based on maritime, fluvial, and terrestrial routes across the Empire (*79*–*84*). From the late first century BCE onward, as Roman territorial control became consolidated, the Rhône-Saône corridor developed into a major axis for both fluvial and terrestrial transport (*79*, *85*). At this strategic crossroads, *Lugdunum* (Lyon, France) became a central node from which the principal roads of Gaul extended toward the Rhine, the Channel via Boulogne-sur-Mer, the Atlantic coast through *Aquitania*, and the Mediterranean (Strabo, IV.6.11). Rather than depending solely on direct routes between producers and consumers, metal circulation was organized around regional storage and redistribution centers, with *Lugdunum* at the heart of the Rhône-Saône-Moselle-Rhine axis (*79*). As Bravard (*52*) has shown, navigation on the Rhône was not confined to traffic toward the Mediterranean but also extended upstream along the Saône toward the northern territories. This longitudinal route connected the Rhône basin with the Rhine region and neighboring river systems, including the Loire and the Seine. Together with the associated terrestrial routes, these waterways formed an integrated multimodal corridor between the Mediterranean and Channel coasts (*79*). From the late first century BCE onward, navigation on the Rhône relied on flat-bottomed vessels adapted to river transport, as shown by shipwrecks discovered at the river ports of *Lugdunum* (*86*) and *Arelate* (*87*). With cargo capacities ranging from several tens to more than 100 tonnes, these vessels carried heavy goods both downstream and upstream and provided the transport capacity required for the large-scale circulation of metals during the Roman period (*79*). This circulation system is consistent with the widespread metallurgical activity reflected in the EMII results (Fig. 6).

Along parts of the Rhône corridor, this territorial organization was reinforced by complementary terrestrial routes, notably the southern branch of Agrippa’s road network between *Lugdunum* and *Arelate*. This route formed a major transport axis within the increasingly dense urban network that developed between the two cities and linked river navigation, road transport, and regional economic activity (*52*, *85*). In this context, the provenance of the Pb used in pipes from Gallo-Roman cities of the Rhône valley provides important evidence for assessing the respective contributions of Iberian and German mining districts to urban development and infrastructure (*64*). Recent Pb isotope analyses of pipes from *Lugdunum* and *Vienna* (Vienne, France), a major center of Pb-pipe production in Roman Gaul (*88*–*91*), indicate that the Rhenish Massif was the predominant source, with additional contributions from British ores (*92*). The agreement between the isotopic signatures of these artifacts and the atmospheric signal provides strong evidence that the patterns of metal production, circulation, and use were closely connected across the western Roman Empire.

### Medieval reorganization and persistence of polycentric metallurgical networks

#### Decline and recovery of atmospheric Pb emissions during the Middle Ages

The transition to the Early Middle Ages (500–1000 CE) marks a continued decline in atmospheric Pb emissions, extending the downward trend that began during the Late Roman period. EMII falls from ∼63 to ∼42, its lowest value since the Late Neolithic/Chalcolithic period (Fig. 6). This trajectory agrees with environmental records spanning from the Iberian Peninsula, the North Atlantic, and Greenland (Fig. 1), which likewise document a sharp reduction in Pb emissions between the fifth and seventh centuries CE (*14*, *15*, *20*, *93*). This decline is generally attributed to the combined effects of several processes rather than to a single dominant cause (*13*, *14*). These include the demographic consequences of the Justinianic plague, climatic instability associated with the Late Antique Little Ice Age (*94*, *95*), and the progressive weakening of western Roman political and economic structures (*96*). The relative importance of these factors nevertheless remains debated, particularly the strength and direction of the causal relationships between climatic stress and societal change (*97*–*99*).

Following this period of decline, EMII rises steadily through the High and Late Middle Ages, reaching ∼91 and ∼130, respectively (Fig. 6). This trend parallels the long-term increase recorded in Greenland ice-core Pb fluxes and lake-sediment Pb concentrations. Although these archives differ in nature, all show a marked rise from the Early to the Late Middle Ages: EMII increases by a factor of ∼3, Greenland Pb fluxes by ∼7 (*15*), and lake-sediment Pb concentrations by nearly an order of magnitude (*22*, *100*). High-resolution environmental records place the culmination of this medieval rise around 1200 CE, when atmospheric Pb pollution reached levels comparable with those recorded during the Early Roman period (*15*, *22*, *100*). More broadly, this rise coincided with sustained economic, demographic, and technological expansion across medieval Europe (*101*–*104*). These high-resolution records also document a subsequent decline during the Late Middle Ages, often associated with major societal disruptions such as the Black Death in the mid-14th century (*15*, *58*, *104*). The broader chronological resolution of EMII, designed to capture integrated long-term signals, does not resolve these shorter-term fluctuations and instead emphasizes the sustained high level of metallurgical activity throughout the High and Late Middle Ages (Fig. 6).

#### A dual-core metallogenic pattern in the Early Middle Ages

Despite the general decline in mining production during the transition to the Middle Ages, the main metallogenic provinces inherited from the Roman period retained a prominent place in the EMII spatial pattern. The Rhenish Massif and German Central Uplands account for 24% of isotopic matches, Britain for 19%, and the southern Massif Central for 15% (Fig. 8). This continuity accords with archaeological syntheses, which indicate that post-Roman mining remained largely concentrated in previously exploited districts, with few new production areas opened during this period (*56*). More broadly, these results suggest that the Roman metal-supply system, based on a polycentric and multimodal network connected by major fluvial corridors, persisted into the Middle Ages. This continuity reflects the long-term importance of river transport, particularly along axes such as the Rhône-Saône corridor, which continued to channel circulation and exchange after the Roman period (*52*, *79*, *80*). Alongside this dominant continental core, the EMII spatial pattern also includes mining districts in the Alps (10%), the Balkans (8%), and Sardinia (8%) (Fig. 8).

This apparent long-term stability, however, conceals marked temporal changes that emerge at finer chronological resolution. During the Early Middle Ages, the metallogenic landscape was organized around two principal centers: central Europe, represented by the Rhenish Massif and German Central Uplands, and the southern Massif Central. Each accounts for ∼20% of the total isotopic matches (fig. S6). British and Balkan mining districts form a secondary group, with each contributing ∼13%. This pattern marks a substantial shift from the Late Roman period. The contributions of central European and British districts decrease by 35 and 33%, respectively, whereas those of the Massif Central and the Balkans increase by 49 and 125%. This configuration closely mirrors current archaeological and historical knowledge of early medieval mining activity and its regional hierarchy. In particular, the prominent role of French districts aligns with the well-documented importance of the Melle mining complex in western France, which was the principal center of Ag production in Western Europe before the 10th century and a major supplier to the Carolingian monetary system (*13*, *15*, *105*, *106*). By contrast, early medieval Pb-Ag production in central Europe and Britain appears more geographically restricted and less intensively documented during this period, before its later expansion. For the medieval period, secure evidence of Pb production in Britain dates from the ninth century in the Derbyshire ore field of the southern Pennines (*107*, *108*). In central Europe, mining activity is only weakly attested before the expansion of the Harz district from the mid-10th century onward (*13*, *109*). Last, the Early Medieval rise of Balkan contributions is also consistent with peatland records that indicate renewed metallurgical activity in southeastern Europe between 800 and 1000 CE (*110*). At the continental scale, however, EMII places the Balkans within a secondary group alongside Britain rather than within the principal early medieval core.

#### Shift toward a northwestern and central European core in the High and Late Middle Ages

From the High Middle Ages onward, the metallogenic landscape shifted toward a northwestern and central European core. The relative contribution of the Rhenish Massif and German Central Uplands increases by 25%, while that of Britain rises by 48%. Conversely, the contributions of the Massif Central and the Balkans decrease by 25 and 47%, respectively (fig. S6). This redistribution agrees with archaeological evidence for the dominant role of British and central European mining districts in Pb-Ag production during the High and Late Middle Ages. In Britain, production was concentrated in the Derbyshire ore field of the southern Pennines, and in other Pennine districts, where substantial output is documented from the 11th to 12th centuries onward (*13*, *15*, *60*, *69*, *109*). In central Europe, major mining regions such as the Harz Mountains (Rammelsberg) and the Erzgebirge (Freiberg) became important production centers during the High Middle Ages (*15*, *69*, *111*).

Outside this dominant core, the Balkans and Sardinia followed contrasting trajectories. The contribution of the Balkans, which had reached a level comparable with that of Britain during the Early Middle Ages, declined markedly thereafter. Sardinia, by contrast, shows a later but pronounced increase of 125% between the High and Late Middle Ages, reaching a contribution comparable to, or greater than, that of the Balkans. This rise agrees with historical and archaeological evidence for the revival of Sardinian Ag production from the mid-13th century onward, particularly in the Iglesiente district under Pisan administration (*111*).

#### From persistent European mining signals to globalized Pb inputs

Beyond the medieval period, EMII values remain high during the Early Modern and Industrial periods (∼125–128) before declining to ∼28 in the Contemporary period (Fig. 6). This trajectory is broadly consistent with Greenland ice-core records, which document a recovery of atmospheric Pb emissions from the mid-15th century. This revival was driven by renewed activity in German mining districts, notably the Erzgebirge and Harz, followed by increased production in British districts such as Derbyshire from the late 17th century onward. Atmospheric Pb emissions then accelerated markedly during the Industrial Revolution and reached a maximum in the mid-20th century (*14*, *15*, *69*, *112*, *113*). EMII geovisualization shows that the Early Modern and Industrial periods were still dominated by northwestern and central European mining regions, with major contributions from British and German districts (Fig. 9).

The absence of a Contemporary EMII peak comparable with the mid-20th-century maximum recorded in Greenland ice cores is expected because, over the past ∼250 years, atmospheric Pb deposition has increasingly reflected a mixture of industrial sources. These include coal combustion, ore smelting, and, during much of the 20th century, leaded gasoline emissions, together with long-range contributions from North America and Asia and the processing of Australian Broken Hill ores in Europe (*15*, *36*, *37*, *100*). The low Contemporary EMII value therefore does not indicate weaker atmospheric Pb emissions. Rather, it reflects the progressive loss of correspondence between atmospheric Pb isotope compositions and distinct European ore signatures as industrial inputs became increasingly diverse and globalized.

### Perspectives

#### Methodological developments

The EMII framework offers an approach for reconstructing large-scale metallurgical dynamics from environmental archives by integrating isotopic signals across space and time. It primarily resolves provenance relationships and provides a relative, semiquantitative measure of temporal changes in the integrated mining-related atmospheric Pb signal recorded by the peatland archive network. At the level of each archive, however, the measured isotopic composition remains fundamentally weighted by the time-varying depositional contributions of the different sources. Although Pb aerosol deposition rates generally decrease exponentially with distance from emission sources [e.g., (*10*, *21*, *114*–*116*)], this physical constraint does not strongly affect the isotopic patterns identified here. The distribution of peatland archives across Europe is uneven, with denser coverage in central Europe and fewer records toward the northern and eastern margins (Fig. 1). However, the comparable distance (∼500 to 1200 km) between the weighted geographic center of the peatland network and the main western European mining districts (Fig. 1), together with the detection of eastern Mediterranean isotopic signatures more than 2500 km away, indicates that the observed spatial patterns are not primarily controlled by proximity effects. These considerations highlight several directions for refining and extending the EMII framework.

A first perspective is to improve the temporal resolution of the EMII framework. In its current form, EMII mainly resolves broad historical periods, as shown by the aggregated distributions in Fig. 6. Extending the analysis to centennial or subcentennial scales would help distinguish changes in metallurgical production and the organization of ancient exchange networks, including short-lived or regionally restricted phases of mining activity. Such refinement will require substantially more isotopic data for each time interval to preserve robust matching statistics. This will involve denser sampling within individual peatland archives, additional high-resolution environmental records, and better harmonization of age-depth models. A finer temporal resolution would also allow direct comparison of the timing of metallurgical activity among mining provinces and reveal regional offsets in their production trajectories.

A second perspective is to extend the EMII framework spatially. The method is not limited to Europe and can be applied to other continental settings where Pb isotope records including ^204^Pb are available and where isotopic databases of ore mineralizations exist. Extensive ore Pb isotope datasets already exist for Europe, North America, and China (*117*, *118*). Comparable resources are also available in the Southern Hemisphere, including continent-wide regolith Pb isoscapes for Australia (*119*) and Pb isotope and *T*_mod_-μ-κ datasets for the Andean metallogenic belts of South America (*120*). Applying a harmonized EMII framework across these regions would allow intercontinental comparisons of metallurgical trajectories and exchange systems and support a transcontinental reconstruction of the emergence, diffusion, and connectivity of metal production networks through time.

A major next step is to move beyond semiquantitative isotopic indicators toward quantitative reconstructions of past metallurgical activity. Converting Pb fluxes reconstructed from palaeoenvironmental records into estimates of extraction volumes remains a long-standing challenge. Early mass-balance calculations and historical syntheses provided first-order estimates of Pb production (*3*, *121*, *122*), while later ice-core and isotopic studies showed that reconstructed atmospheric Pb fluxes broadly track independently established histories of metallurgical production, although they did not directly quantify extraction volumes (*17*, *23*, *123*, *124*). Further progress could come from combining EMII with physically based atmospheric transport models, such as FLEXPART (*14*, *15*, *125*), to calibrate isotopic match intensities against modeled source-receptor sensitivity fields. This approach could help translate archive-derived isotopic signals into quantitative estimates of atmospheric Pb deposition and, ultimately, absolute Pb fluxes (tonnes per year). These fluxes could then be related to galena extraction through mass-balance scaling relationships, enabling quantitative comparisons of metallurgical production among provinces through time.

#### Conceptual framework

A final perspective is to develop an integrative framework capable of reconstructing the full life cycle of metals, from geological resources to finished products and their circulation within economic systems. At the upstream end, mining archaeology provides essential constraints on the chronology and spatial organization of extraction districts and can also support estimates of production volumes through large-scale syntheses and mining databases (*126*). In combination with archaeometric approaches (*63*), it accounts for the internal variability of ore deposits, because Pb isotope signatures may differ considerably within a single mining district because of geological heterogeneity (*22*, *118*). These complementary approaches strengthen source attribution and improve the temporal and spatial resolution at which active mining districts can be identified. At the downstream end, numismatics and archaeometry provide key evidence for the transformation of ores into metal products, such as coinage, and their integration into economic systems. Pb isotopic analyses of Ag coinage and metal artifacts make it possible to trace metal sources after metallurgical processing, while documenting processes of standardization, circulation, and redistribution within monetary and economic networks (*4*–*6*, *25*–*28*, *50*, *51*, *76*). These datasets therefore establish a direct link between mining systems and economic organization and provide key insights into the organization and evolution of ancient economies. Evidence for fluvial and maritime routes, transport capacities, and shipwreck distributions, increasingly compiled in large-scale databases (*127*) and combined with network models of connectivity and transport costs (*128*), reveals the transport framework through which metals were moved, redistributed, and incorporated into regional and supra-regional economic systems.

The quest for metals was closely tied to the growth of urban centers and states, whose increasing demand drove the expansion of mining and exchange networks across progressively larger territories (*1*, *85*). EMII adds an environmental dimension to this reconstruction by showing how metallurgical activity left an atmospheric imprint that changed in intensity and geographical extent through time. This convergence between environmental records and the archaeological history of metal supply calls for a framework capable of explaining how urban growth, resource exploitation, and environmental change became mutually reinforcing over the *longue durée*.

The concept of an urban palaeoanthropocene provides a suitable analytical framework for describing these anthropization processes by bringing together environmental signals, socio-technical systems, and economic networks. Several long-term records reveal parallel changes in urban expansion, land use, and metallurgical activity. Global syntheses of land-use change indicate a marked intensification of anthropogenic pressure from the late Holocene onward, with a notable inflection around the early first millennium BCE associated with the expansion of urbanized landscapes (*129*). Greenland ice-core records likewise document a rise in atmospheric Pb emissions at the hemispheric scale during this period (*14*, *15*). The temporal correspondence between these records suggests that urban expansion and metallurgical production were closely connected components of long-term environmental change. Within the urban palaeoanthropocene framework, the links between metal production, exchange networks, and urban consumption can be understood as a tripartite system sustained by positive feedbacks that contributed to long-term anthropization processes. The development of urban centers increased demand for metallic resources. This growing demand drove the expansion of supply areas toward new mining districts. The exploitation of these resources and the exchange networks through which metals circulated, in turn, contributed to the emergence and growth of urban centers. This growth further increased demand. This feedback mechanism highlights the central role of metal exploitation and circulation in patterns of human expansion and the progressive transformation of natural environments.

The spatial patterns identified by EMII therefore have broader implications. The transition from the Bronze Age to the Iron Age marks a shift from an eastern Mediterranean system organized around maritime exchange networks to a western, polycentric, and multimodal system. This shift reflects not only a reorganization of metal supply but also a redistribution of anthropogenic pressures. Rather than indicating a unidirectional process of diffusion from a single chronological threshold, these results reveal a networked and regionally differentiated expansion of anthropization across multiple spatial scales. Together, they suggest that the palaeoanthropocene, particularly in its urban expression, should be understood not as a sudden event but as a long-term trajectory that emerged from interactions among urban development, resource exploitation, and environmental transformation. By linking environmental archives with archaeological and historical evidence, EMII contributes to a more integrated reconstruction of the economic foundations of premodern world systems, the anthropization processes associated with them, and their lasting environmental legacies.

## MATERIALS AND METHODS

### Study area

The Dig1Ter peat core (45.34804°N, 4.47731°E) was recovered from an ombrotrophic peatland in the Massif du Pilat, ∼12 km south of Saint-Etienne (Massif Central, France; fig. S1). The site lies within the Natura 2000 protected area FR8201761, at an elevation of 1055 m, and is underlain by biotite-rich monzonitic leucogranite (*130*). A drainage ditch borders the southern edge of the peatland, while *Pinus sylvestris* covers most of its 2.4-ha surface. The western and northern sections are the most open parts of the peatland but show signs of substantial degradation. The peatland is adjacent to the Semène River and contributes to the water supply of this river, which is a right-bank tributary of the Loire River.

The development of the Digonnière peatland is documented by radiocarbon dates obtained from basal peat layers in previously recovered cores, which place the onset of peat formation between 10,500 and 9900 calibrated years before the present (cal yr B.P.) (*131*). Palaeoecological reconstructions indicate that early vegetation was initially dominated by hazel (*Corylus*), followed by the establishment of a mixed deciduous forest composed of *Quercus*, *Tilia*, *Ulmus*, and *Fraxinus* between 9000 and 7000 cal yr B.P. (*132*). Around 5000 cal yr B.P., a cold and humid episode favored the development of a beech-fir (*Fagus*-*Abies*) forest around the site. In the upper part of the pollen record, increases in cereal-type and Poaceae pollen, together with *Calluna* and *Pinus*, indicate a marked opening of the landscape and drying of the peatland. This signal and the establishment of conifer plantations are recorded in the uppermost 15 cm of the sequence and are attributed to land-use changes that probably began during the second half of the 19th century (*132*). A 2-m-long peat core was recovered from the northern part of the peatland, ∼45 m from the left bank of the Semène River. The stratigraphy comprises three distinct units (Fig. 2): (1) root-rich, mineralized peat (0 to 20 cm); (2) homogeneous, moderately decomposed black sapric peat (20 to 169 cm); and (3) organic-rich sandy layer (170 to 200 cm).

### Dating methodology

#### Radiocarbon dating and modeling

The Late Holocene chronology of the Dig1Ter core is based on six accepted radiocarbon dates obtained from carefully selected macrofossils, mainly *Salix*/*Betula* twigs and *Eriophorum* remains (Fig. 2 and table S2). One date, obtained from bulk peat near the top of the core, was excluded because it yielded an anomalously old age relative to the ^210^Pb chronology (see the “Radionuclide dating and modeling” section). Five radiocarbon samples were prepared at the Centre de Datation par le Radiocarbone in Lyon (CDRC, Univ. Lyon 1) and measured at the ARTEMIS facility in Saclay, whereas two additional samples were analyzed at the Poznań Radiocarbon Laboratory. All radiocarbon ages were calibrated at 2σ using the IntCal20 calibration curve (*133*), and the age-depth model was constructed using the “rbacon” package (v.3.3.1) in R (*134*).

#### Radionuclide dating and modeling

The chronology of the upper 15 cm of the Dig1Ter core was established using fallout radionuclides (^210^Pb, ^137^Cs, and ^241^Am) (Fig. 2). The radionuclide ^210^Pb, a product of the ^238^U decay series, accumulates naturally in lake and peat sediments through atmospheric deposition (half-life of 22.3 years). Due to the long-term stability of Pb isotopes in sediments, the decay of excess ^210^Pb is widely used to reconstruct recent accumulation rates (*135*, *136*). However, the retention mechanisms and efficiency of ^210^Pb in peatland vegetation and, consequently, within peat deposits remain incompletely understood. Discrepancies may therefore arise between atmospheric fallout and ^210^Pb measured in *Sphagnum* mosses (*137*, *138*).

The anthropogenic radionuclides ^137^Cs and ^241^Am were also measured. Both were released primarily during atmospheric nuclear weapons testing (NWT), with maximum fallout around 1962–1963. A secondary ^137^Cs peak associated with the Chernobyl nuclear accident occurred in 1986 and is widely used as a chronological marker across Europe (*139*, *140*). Although ^137^Cs may be mobile within peat profiles and is therefore unsuitable for quantitative dating, ^241^Am remains stable in many peatlands and provides a robust stratigraphic marker of NWT fallout (*141*, *142*).

All radionuclide activities were measured nondestructively by gamma spectrometry at the Modane Underground Laboratory (LSM), using detectors operated by the LSCE (Laboratoire des Sciences du Climat et de l’Environnement, UMR 8212 CNRS). Age-depth models for the uppermost 15 cm were constructed using the constant flux–constant sedimentation and constant rate of supply models implemented in the “serac” package (v.1.2) in R (*136*).

### Geochemical analyses

#### Major and trace element analysis

About 50 bulk peat samples were selected for elemental geochemical analysis. Each dry sample was powdered using an agate mortar. Approximately 50 mg of each sample were acid digested in the clean laboratory at the Ecole Normale Supérieure de Lyon as follows. A mixture of 3 ml of concentrated HNO_3_ and 1 ml of Suprapur 30% H_2_O_2_ was added to each sample. The beakers were left at room temperature for 24 hours with their lids loosely screwed on to degas before being tightly closed and placed on a hot plate at 120° to 130°C for 48 hours. After evaporation to dryness, this step was repeated once. Complete digestion was achieved using distilled 6 M HCl. After evaporation to dryness, the samples were redissolved in 3 ml of distilled 7 M HNO_3_, evaporated to dryness, and redissolved in dilute distilled HNO_3_ for major and trace element analysis by quadrupole inductively coupled plasma mass spectrometry (iCAP Q, Thermo Fisher Scientific) using In (2 parts per billion) as the internal standard. The major and trace element data are listed in table S4.

#### Pb isotope analysis

An aliquot of ∼500 mg of each sieved sample was sequentially leached with hot chloroform and 1 M distilled HBr, using a protocol adapted from methods originally developed for sedimentary matrices and subsequently applied to peat archives (*143*–*147*). Heating and ultrasonication steps were alternated throughout the procedure. The leachate fractions, representing the anthropogenic component of Pb, were evaporated to dryness and digested in a 3:1 mixture of distilled concentrated HNO_3_:H_2_O_2_ followed by distilled 6 M HCl as described above. The samples were redissolved in distilled 1 M HBr and Pb subsequently separated on a 0.5-ml anion-exchange column (AG1-X8, 100 to 200 mesh). The sample matrix was eluted with 1 M HBr and the Pb fraction collected with distilled 6 M HCl. Pb isotopic ratios were measured by multicollector inductively coupled plasma mass spectrometry (Nu Plasma 500 HR, Nu Instruments) at the Ecole Normale Supérieure de Lyon. Thallium was added to the samples and the NIST 981 Pb standard to correct for instrumental mass fractionation. The NIST 981 Pb standard was measured systematically after every one to two samples throughout the individual day-long analytical sessions to ensure accurate sample-standard bracketing using the NIST 981 values of Eisele *et al.* (*148*). The external reproducibility was better than 100 parts per million (ppm; 0.01%) for ^204^Pb-normalized ratios and better than 50 ppm (0.005%) for ^207^Pb/^206^Pb and ^208^Pb/^206^Pb. In-run analytical uncertainties (2SE) for both samples and standards were consistently smaller than the external reproducibility. The measured Pb isotopic ratios and the derived geochemical parameters *T*_mod_ [Pb model ages in million years (Ma)], μ (mu; the ^238^U/^204^Pb ratio), and κ (kappa; the ^232^Th/^238^U ratio) are listed in table S4.

### Processing of Pb isotope data for provenance analysis

#### Archaeometry-based algorithmic approach for Pb ore provenancing

The provenancing algorithm applied in this study builds on recent advances in isotopic data modeling that account for both mass-dependent isotope fractionation and the full covariance structure of analytical uncertainties (*5*, *6*, *27*, *28*). A key feature of the method is that it compares each environmental or archaeological sample not directly with individual ore analyses, but with the mass-fractionation line defined for each reference ore field. These lines are mathematical expressions of how Pb isotopic ratios (^206^Pb/^204^Pb, ^207^Pb/^204^Pb, and ^208^Pb/^204^Pb) covary as a function of mass-dependent fractionation, and they define the isotopic signature of a given ore field in three-dimensional space.

To evaluate compatibility between a sample and a potential ore source, the algorithm calculates a chi-squared distance (*d*) between the isotopic composition of the sample and the mass-fractionation line associated with each reference ore field. This statistical distance quantifies how closely the sample’s isotopic ratios (^206^Pb/^204^Pb, ^207^Pb/^204^Pb, and ^208^Pb/^204^Pb) align with the expected variations along each district’s reference line. The distance is computed using a generalized chi-squared metric, which integrates both the isotopic values and their analytical uncertainties, while fully accounting for the covariance structure of the measurements. Because the squared distance *d^2^* follows a chi-squared distribution with two degrees of freedom, provenance compatibility can be evaluated at adjustable and quantifiable levels of statistical confidence. In this study, a match is considered plausible when *d* falls below a defined threshold. The rationale for the choice of this threshold, particularly the criterion *d* < 3, is detailed below.

Natural atmospheric Pb inputs, derived mainly from diffuse crustal and volcanic sources, are characterized by broad, nonspecific isotopic compositions (*40*) and limited spatial and temporal coherence. By contrast, the reference database (Fig. 3) used in this study is restricted to galena mineralizations from defined metallogenic provinces. Within this framework, the archaeometry-based algorithmic approach for Pb ore provenancing (*27*) identifies statistically significant correspondences between environmental samples and these ore fields in multidimensional isotope space. Diffuse natural background inputs are unlikely to produce recurrent and spatially structured matches within the ore database, whereas anthropogenic Pb derived from galena generates coherent patterns across multiple samples and chronological periods. All provenance analyses were conducted using the Lyon Pb Isotope Database, which comprises Pb isotope data for ∼7000 galena samples from Europe and the Mediterranean (Fig. 3). The database includes sample identifiers, geographical origins, coordinates, isotope ratios (^206^Pb/^204^Pb, ^207^Pb/^204^Pb, and ^208^Pb/^204^Pb), and the derived parameters [*T*_mod_, μ (^238^U/^204^Pb), and κ (^232^Th/^238^U)]. Initially compiled by Delile *et al.* (*143*, *144*) and Blichert-Toft *et al.* (*39*), it was subsequently expanded and curated before its public release in 2025 (*5*, *27*, *28*). The database is intended to be merged with the Bochum GlobalID database (*33*) to support its continued development. Both the database and the provenancing algorithm used in this study are openly available through Zenodo (https://zenodo.org/records/15119912), ensuring full reproducibility of the provenance analysis.

#### Definition and computation of the EMII

The EMII was developed to quantify the degree to which the isotopic signal preserved in atmospheric environmental archives matches known Pb-Ag ore signatures. It enables diachronic comparisons of ore-related anthropogenic Pb signals across time and space. For each peat sample *i* assigned to chronological period *p*, the algorithm counts the number *M*_*i*,*p*,*d*_ of statistically significant ore-source matches. A match is identified when the Pb isotopic composition of the peat sample falls within a chi-squared distance *d* < 3 of the mass-fractionation line associated with an ore field. These match counts are then summed across the *S*_*p*_ peat samples assigned to period *p* and divided by *S*_*p*_. EMII therefore represents the mean number of statistically significant ore-source matches per peat sample for a given chronological period

$$\text{EMI}I_{p,\;d}=\frac{\sum_{i=1}^{S_{p}}M_{i,p,d}}{S_{p}}$$

where $M_{i,p,d}$ is the number of statistically significant ore-source matches identified for peat sample *i*, assigned to period *p*, using the chi-squared distance threshold *d* < 3; and $S_{p}$ is the total number of peat samples assigned to period *p*.

As expected from the cumulative nature of the matching procedure, the total number of isotopic matches per period is positively correlated with the number of available peat samples [correlation coefficient (*r*) = 0.64]. By contrast, EMII, expressed as the mean number of matches per sample, shows only a weak correlation with sample count (*r* = −0.14). This limited relationship indicates that uneven sample availability has little influence on EMII and supports comparisons among periods represented by different numbers of samples. The European peatland compilation is summarized across 12 chrono-cultural periods (Fig. 6), whereas only 11 of these periods are represented in the Dig1Ter core because no sample falls within the Early Modern interval (Fig. 5F).

#### Rationale for the choice of distance threshold (d)

To evaluate the plausibility of a match between an environmental sample and an ore source, the algorithm calculates the chi-squared distance *d* between the isotopic composition of the sample and the mass-fractionation lines associated with each reference ore field (*27*). The squared distance *d*^2^ follows a chi-squared distribution with two degrees of freedom, as determined by the dimensionality of the isotopic space (^206^Pb/^204^Pb, ^207^Pb/^204^Pb, and ^208^Pb/^204^Pb). The threshold *d* = 3 corresponds to χ^2^ = 9; the criterion *d* < 3 therefore defines an ∼99% confidence region for isotopic compatibility. This threshold was recommended by Albarède *et al.* (*5*) as an appropriate balance between limiting false-positive matches and retaining plausible correspondences, particularly where analytical uncertainties are greater or reference isotopic fields are less well constrained. The spatial patterns derived from EMII geovisualization and the temporal EMII trends reported here are insensitive to the precise value of *d* within a reasonable range. Comparative tests using thresholds of *d* < 1, *d* < 2, *d* < 4, and *d* < 5 produced the same overall temporal and geographical patterns and identified the same principal metallogenic regions. Although the absolute number of matches increases with larger *d*, the relative distribution of matches across time and space remains stable. This stability supports the robustness of the selected threshold.

#### Assessment of potential spatial bias in EMII values

One potential concern when using the EMII is that uneven spatial coverage of the ore database could introduce bias. Regions with denser isotopic documentation might appear to produce more matches simply because they are represented by more galena analyses near a given isotopic composition. Several independent observations nevertheless indicate that this effect has only a limited influence on EMII results.

First, the provenancing algorithm does not compare each environmental sample with individual ore analyses. Instead, it evaluates proximity to the mass-fractionation line statistically defined for each reference ore field. These lines describe the internal isotopic geometry of each field from the distribution and covariance of the three ratios (^206^Pb/^204^Pb, ^207^Pb/^204^Pb, and ^208^Pb/^204^Pb). The number of galena analyses therefore does not directly increase the number of potential matches, although it affects how well each reference line is constrained. More extensively sampled ore fields consequently do not automatically exert greater statistical influence on EMII.

Second, we compared the distributions of isotope ratios in the galena reference database (Fig. 3), the peat samples from environmental archives, and the matches identified by the algorithm (fig. S7). For ^207^Pb/^204^Pb, the distribution of matches is strongly correlated with that of the ore dataset (*r* = 0.94), and both display similar Gaussian profiles and inflection points. By contrast, the match distributions for ^206^Pb/^204^Pb and ^208^Pb/^204^Pb differ markedly from those of the galena dataset. The ore data show clear bimodal distributions, with secondary peaks at ∼18.7 to 19.0 for ^206^Pb/^204^Pb and ∼38.75 to 39.0 for ^208^Pb/^204^Pb, whereas these peaks are absent from the match distributions. The latter remain unimodal, which indicates that the matches are not simply inherited from variations in ore-data density but result from the selective filtering performed by the algorithm.

Third, the peat samples, compiled independently of the ore database, show Pb isotope ratio distributions that correlate strongly with those of the ore-source fields (*r* = 0.64, 0.90, and 0.67 for ^206^Pb/^204^Pb, ^207^Pb/^204^Pb, and ^208^Pb/^204^Pb, respectively). This correspondence may reflect the long-term imprint of mining-related emissions on atmospheric Pb deposition across Europe. Particularly notable are the primary modes observed in the distribution of the peat samples near ∼18.35 to 18.40 (^206^Pb/^204^Pb), ∼15.67 (^207^Pb/^204^Pb), and ∼38.37 to 38.65 (^208^Pb/^204^Pb), along with a minor secondary peak around 38.8 to 38.9 (^208^Pb/^204^Pb).

Together, these observations indicate that the matching procedure does not simply reproduce the density structure of the ore reference database. Instead, it applies stringent multivariate compatibility criteria based on the full geometry of mass-fractionation trajectories in three-dimensional Pb isotope space and the covariance structure of analytical uncertainties. Although no database can be spatially or compositionally exhaustive, the observed decoupling between ore-sample frequency and match occurrence supports the use of EMII as a relative, semiquantitative measure of the temporal and spatial distribution of mining-related atmospheric Pb signals.

#### Geovisualization of EMII results

To visualize the spatiotemporal distribution of ore-source matches identified by EMII, we produced a series of period-specific maps in ArcGIS Pro (v.3.4, Esri). Each map combines two complementary layers:

1) Density polygons: Feature binning was used to overlay the study area with a regular grid of hexagonal cells, each covering 4000 km^2^. The number of isotopic matches within each cell was calculated and color coded to represent their spatial density. Only cells containing at least one match are displayed; zero is therefore not included as a mapped class, and the lightest color represents one match. The upper limit of the color scale corresponds to the highest number of matches recorded within a single cell for the chronological period concerned.

2) Proportional circles: These represent the total number of matches at aggregated locations. The Feature Clustering tool was used to group geographically close ore-source matches and display them as circles of varying size. Circle size represents the absolute number of matches within each cluster, while labels indicate the corresponding counts. This layer facilitates comparisons among regions and, provided that a common symbol-size scale is used, among chronological periods.

## Supplementary Materials

### This PDF file includes:

Figs. S1 to S7 Tables S1 to S4 References
