# Supplementary material for: Rise and spread of lead-silver metallurgy deciphered from peatland archives
Source: Sci Adv. 2026 Oct 7;12(41):eaec1413. doi: 10.1126/sciadv.aec1413 (PMC13644759; doi:10.1126/sciadv.aec1413)
Supplement: Supplementary file 1 [file sciadv.aec1413_sm.pdf]

Science Advances
AAAS

Supplementary Materials for

Rise and spread of lead-silver metallurgy deciphered from peatland archives

Hugo Delile *et al.*

Corresponding author: Hugo Delile, hugo.delile@cnrs.fr

Sci. Adv. **12**, eaec1413 (2026)
DOI: 10.1126/sciadv.aec1413

This PDF file includes:

Figs. S1 to S7
Tables S1 to S4
References

Fig. S1. Location of the Dig1ter core and the pollen core previously studied by Argant and Cubizolle (132) in the Digonnière peatland, Pilat Massif (Massif Central, France). Basemap imagery: BD ORTHO® 2022, source: IGN, under the French Open Licence 2.0 (Licence Ouverte 2.0).

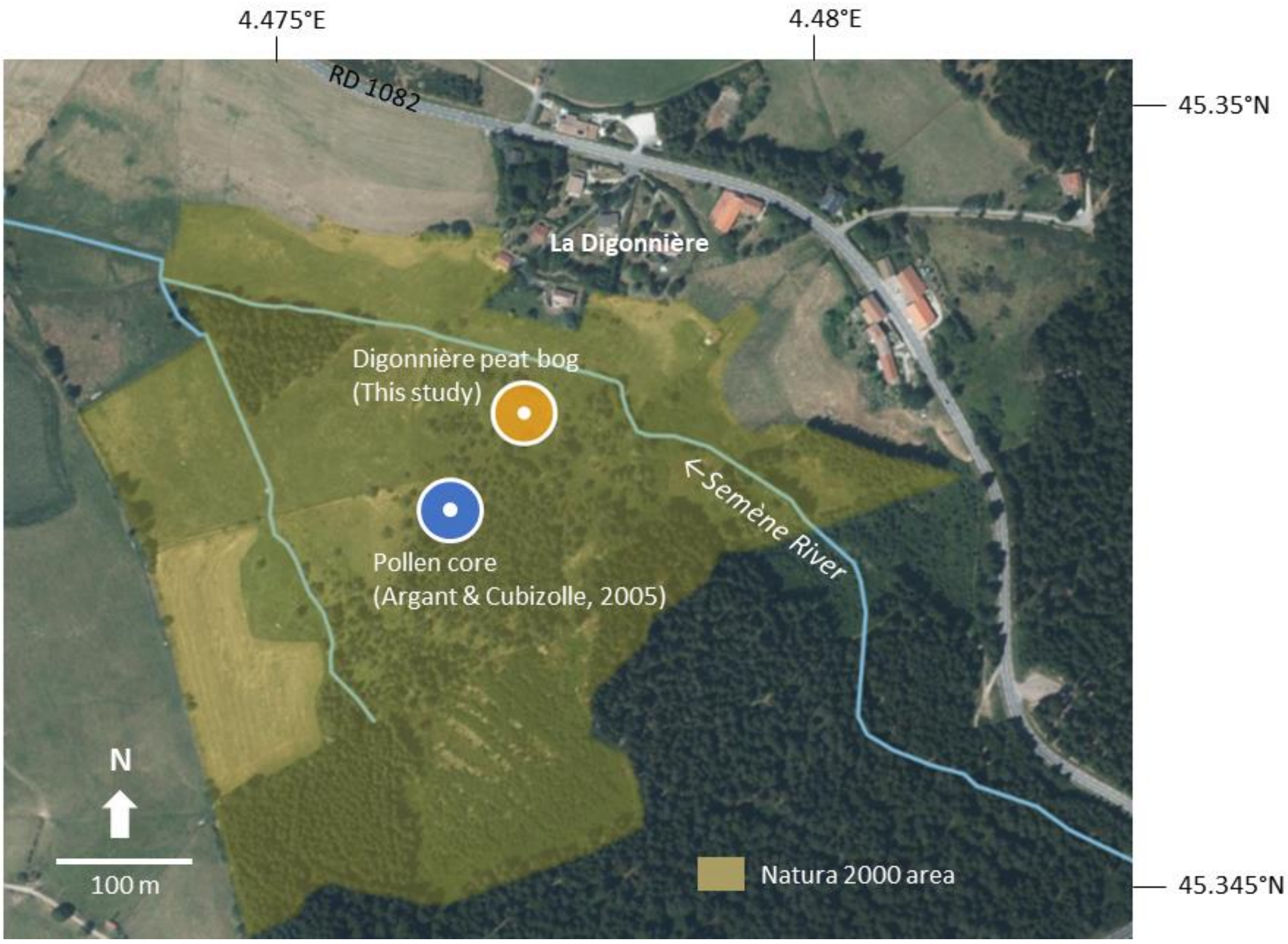

Fig. S2. Downcore variations in Pb isotope compositions, derived geochemical parameters, and Pb enrichment in the Dig1ter core. Depth profiles show $^{206}Pb/^{204}Pb$, $^{207}Pb/^{204}Pb$, $^{208}Pb/^{204}Pb$, T_{mod} (Pb model age, Ma), κ ($^{232}Th/^{238}U$), μ ($^{238}U/^{204}Pb$), and the Pb enrichment factor (EF_{Pb}) throughout the Dig1ter peat core.

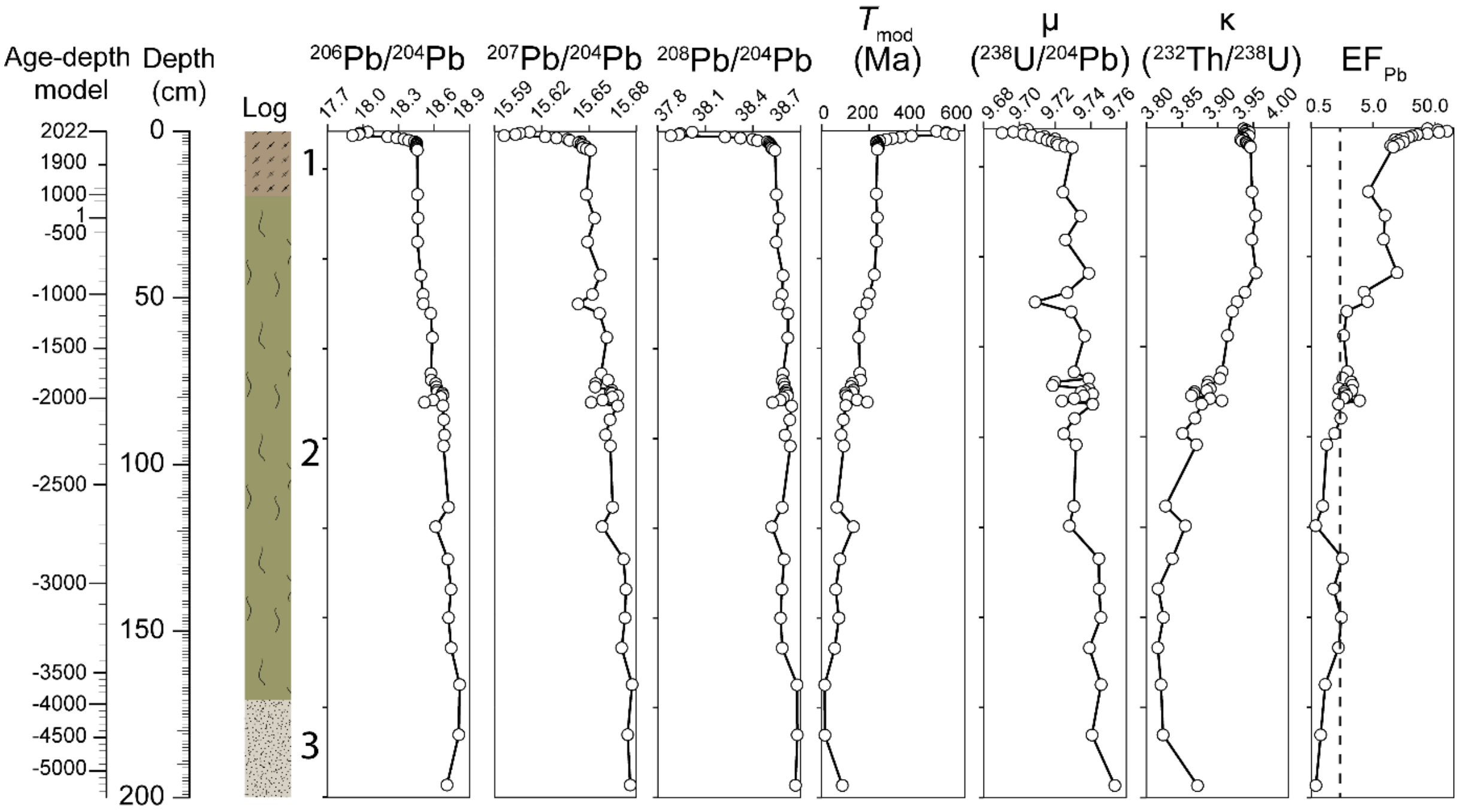

Log: 1) Mineralized peat, 2) Hemic to sapric peat, 3) mineral level with organic matter

Fig. S3. Biplots of $^{206}Pb/^{204}Pb$ versus $^{208}Pb/^{204}Pb$ for atmospheric environmental archives. The five panels show the distribution of Pb isotope compositions for all samples in the database, followed by separate subsets for ice cores, lake sediments, lagoon deposits, and peatlands.

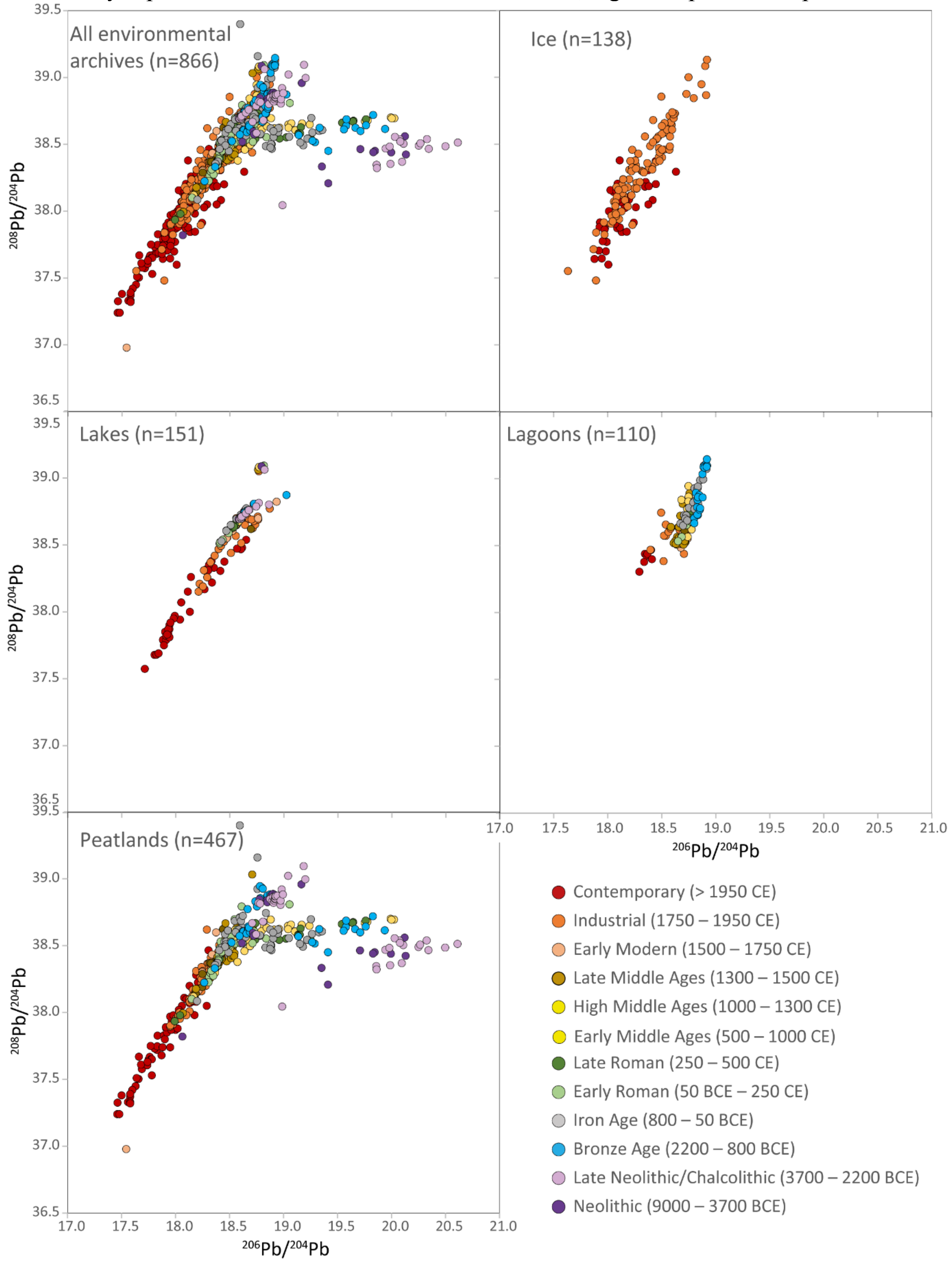

Fig. S4. Biplots of $^{206}Pb/^{204}Pb$ versus $^{207}Pb/^{204}Pb$ for atmospheric environmental archives. The five panels show the distribution of Pb isotope compositions for all samples in the database, followed by separate subsets for ice cores, lake sediments, lagoon deposits, and peatlands.

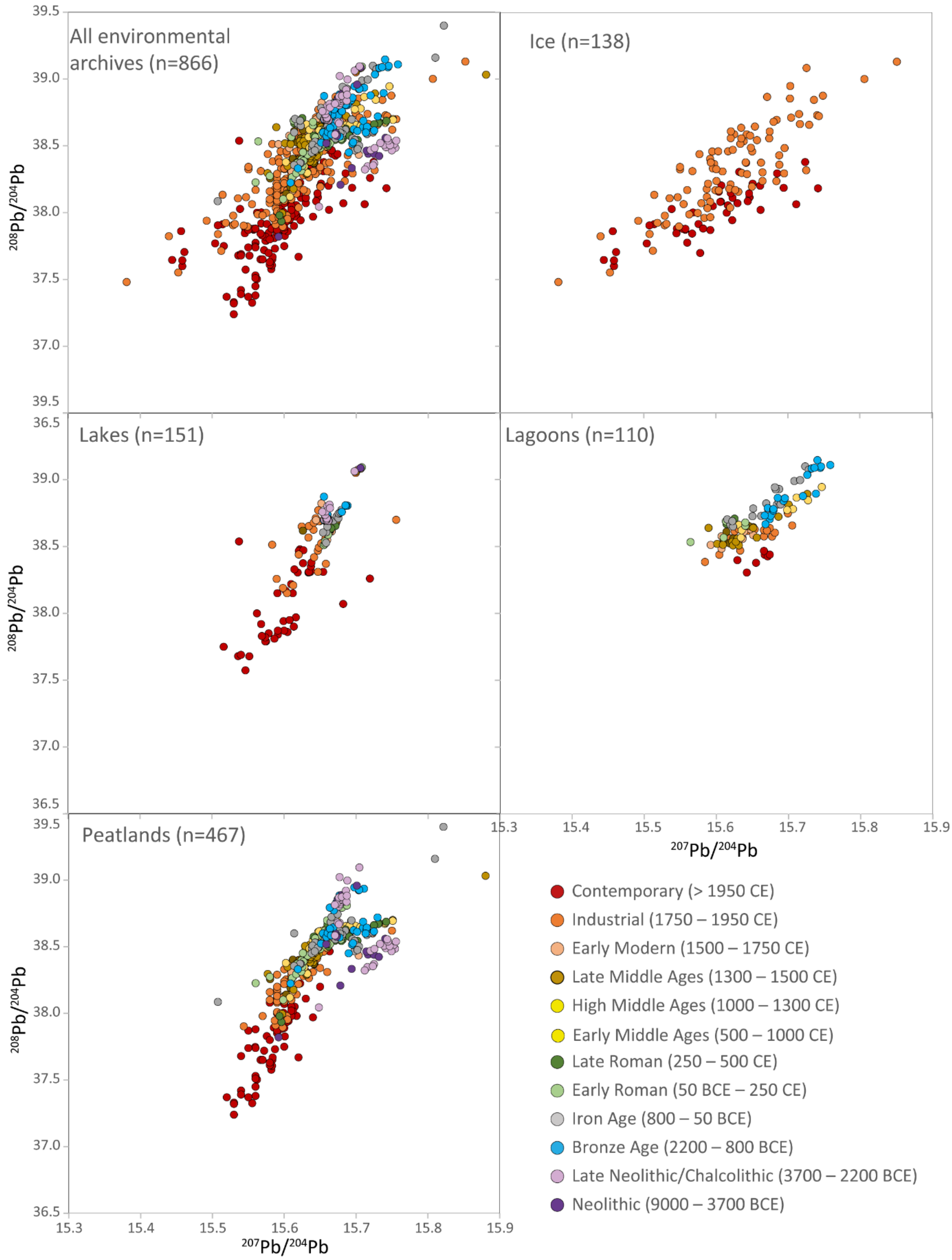

Fig. S5. Spatial distribution of potential Pb–Ag source regions inferred from EMII matches across Europe and the Mediterranean during the Early and Late Roman periods. For each chronological period, Pb isotope compositions measured in the 19 peatland records (Fig. 1; Table S1) were compared with galena Pb isotope signatures from the Lyon Pb Isotope Database (Fig. 3) using EMII. Colour-coded hexagonal cells, each covering 4,000 km², show the number of isotopic matches within each cell. Proportional circles generated by spatial clustering represent the total number of matches at each aggregated location, with numerical labels indicating the corresponding counts. The mapped areas represent potential source regions inferred from isotopic compatibility, irrespective of their documented period of exploitation. Map image is the intellectual property of Esri and is used herein under license. Copyright © 2026 Esri and its licensors. All rights reserved.

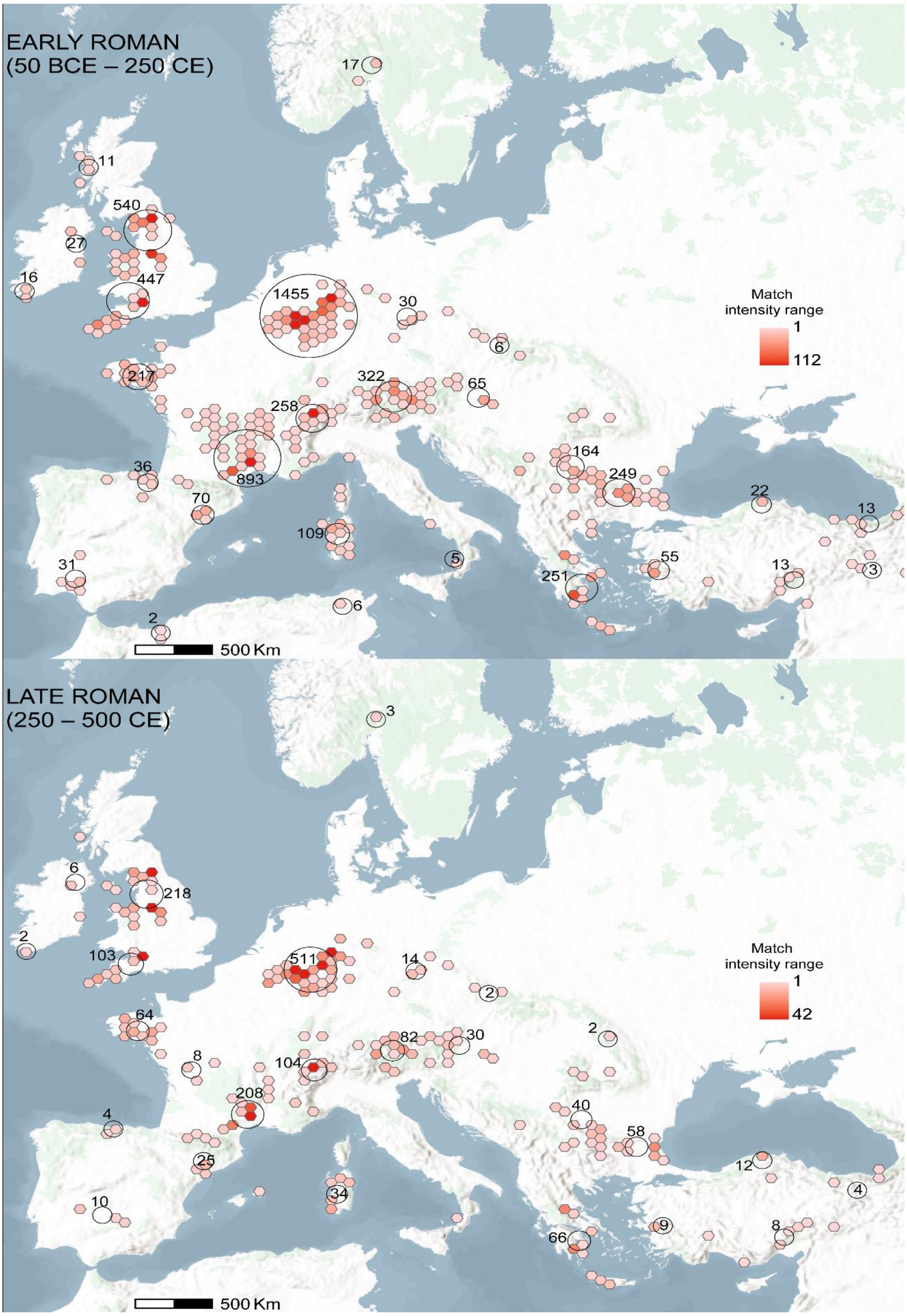

Fig. S6. Spatial distribution of potential Pb–Ag source regions inferred from EMII matches across Europe and the Mediterranean during the Early, High, and Late Middle Ages. For each chronological period, Pb isotope compositions measured in the 19 peatland records (Fig. 1; Table S1) were compared with galena Pb isotope signatures from the Lyon Pb Isotope Database (Fig. 3) using EMII. Colour-coded hexagonal cells, each covering 4,000 km², show the number of isotopic matches within each cell. Proportional circles generated by spatial clustering represent the total number of matches at each aggregated location, with numerical labels indicating the corresponding counts. The mapped areas represent potential source regions inferred from isotopic compatibility, irrespective of their documented period of exploitation. Map image is the intellectual property of Esri and is used herein under license. Copyright © 2026 Esri and its licensors. All rights reserved.

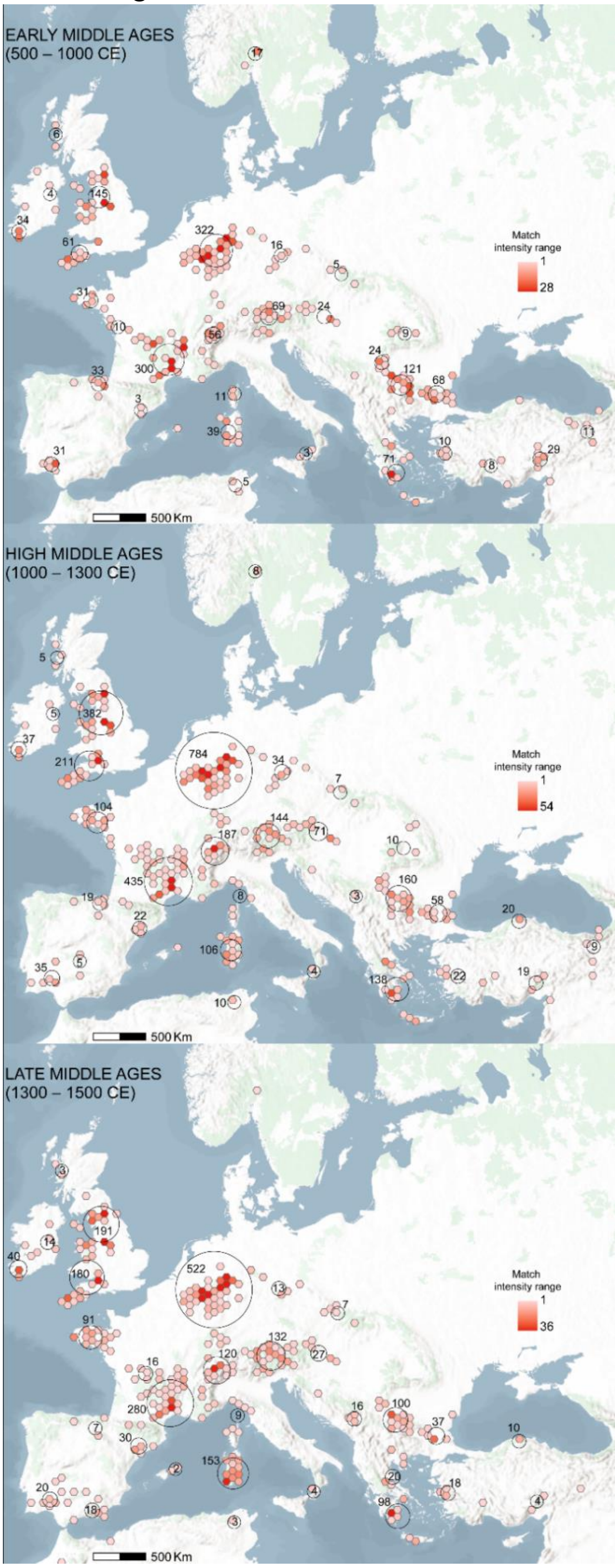

Fig. S7. Comparison of Pb isotope frequency distributions among ore samples, peat samples, and statistically identified matches. The figure compares the frequency distributions of $^{206}Pb/^{204}Pb$, $^{207}Pb/^{204}Pb$, and $^{208}Pb/^{204}Pb$ across three datasets: (i) galena samples from the reference ore database (orange histograms; Fig. 3), (ii) peat samples from environmental archives (green histograms; Fig. 1), and (iii) isotope-ratio values associated with statistically significant ore-source matches identified by the archaeometry-based algorithm (blue histograms). Here, "matches" refer to statistically significant isotopic compatibilities between peat samples and ore-source fields, as defined by the similarity threshold described in the Methods. Correlation coefficients (r, reported in each panel) quantify the similarity between the ore-sample distribution and the distributions of matches (middle row) and peat samples (lower row).

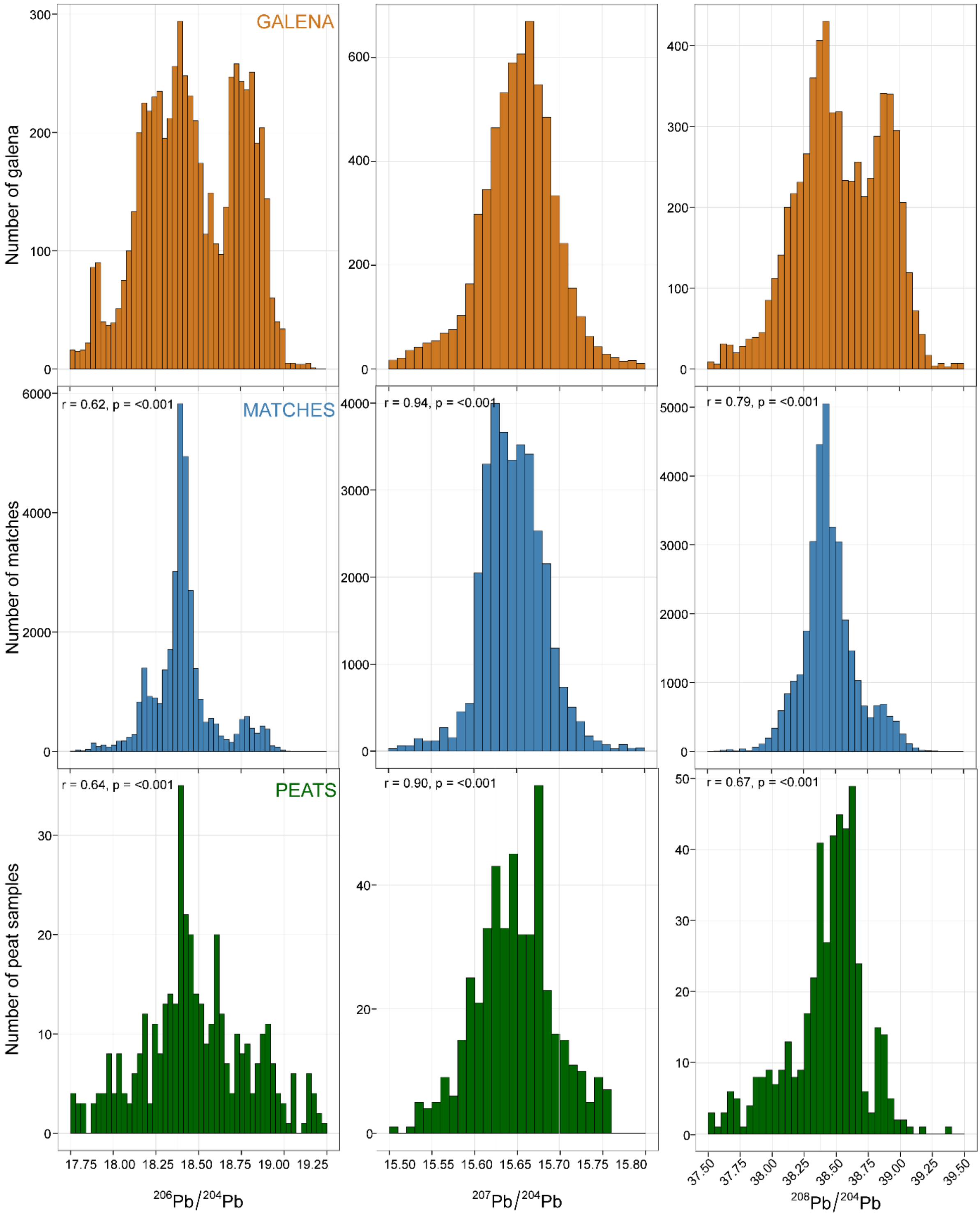

Table S1. Atmospheric environmental archives reporting ^{204}Pb-normalised isotope ratios. The table lists all atmospheric environmental archives compiled in this study. Only peatland records were retained for the quantitative EMII analysis, whereas other archive types were included for comparative and descriptive purposes (Fig. 1; Methods).

Code used in Fig. 1	N samples	Archive	Name	Region	Country	North	East	References	URL
IC1	138	Ice core	Summit-2010	Summit Central Station	Greenland	72.6	-38.3	Wensman et al. 2022 (36)	https://doi.org/10.1016/j.ancene.2022.100340
L1	33	Lacustrine core	BV and PC cores	Lake Geneva	Switzerland	46.512	6.585	Monna et al. 1999 (149)	https://doi.org/10.1021/es9902468
L2	15	Lacustrine core	Lake Anterne	Haute Savoie	France	45.991	6.797	Arnaud et al. 2004 (150)	https://doi.org/10.1039/B314947A
L3	11	Lacustrine core	Le Bourget Lake - B16	Savoie	France	45.758	5.857	Arnaud et al. 2004 (150)	https://doi.org/10.1039/B314947A
L4	32	Lacustrine core	Lake Blanc	Isère	France	45.115	6.106	Garçon et al. 2012 (151)	https://doi.org/10.1016/j.apgeochem.2011.12.010
L5	7	Lacustrine core	Lago Accesa	Tuscany	Italy	42.988	10.895	Pasquetti et al. 2025 (152)	https://doi.org/10.1016/j.ancene.2025.100464
L6	53	Lacustrine core	Laguna Roya	Zamora	Spain	42.136	-6.794	Griffore et al. 2023 (153)	https://doi.org/10.1016/j.ancene.2023.100375
G1	110	Lagoon core	Pierre Blanche PB06	Hérault	France	43.506	3.873	Elbaz-Poulichet et al. 2011 (154)	https://doi.org/10.1021/es2004599
P1	48	Peat core	Digonnière	Pilat	France	45.348	4.476	This study	This paper
P2	20	Peat core	Praz Rodez	Jura	Switzerland	46.565	6.172	Weiss et al. 1999 (155)	https://doi.org/10.1021/es980882q
P3	11	Peat core	Etang de la Gruere	Jura	Switzerland	47.24	7.05	Weiss et al. 1999 (155)	https://doi.org/10.1021/es980882q
P4	13	Peat core	Tourbière des Genevez	Jura	Switzerland	47.239	7.049	Weiss et al. 1999 (155)	https://doi.org/10.1021/es980882q
P5	23	Peat core	Schopfenwaldmoor	Bern Oberland	Switzerland	46.743	7.847	Weiss et al. 1999 (155)	https://doi.org/10.1021/es980882q
P6	12	Peat core	Gola di Lago	Lugano District	Switzerland	46.103	8.964	Weiss et al. 1999 (155)	https://doi.org/10.1021/es980882q
P7	46	Peat core	Füramoos	Baden-Württemberg	Germany	47.991	9.887	Kern et al. 2021 (48)	https://doi.org/10.1016/j.ancene.2021.100310
P8	23	Peat core	Fangeas	Hautes-Alpes	France	44.714	6.447	Py et al. 2014 (156)	https://doi.org/10.1016/j.quaint.2014.05.002
P9	48	Peat core	Narses Mortes	Mont Lozère	France	44.446	3.611	Baron et al. 2005 (77)	https://doi.org/10.1021/es048165l
P10	16	Peat core	Clefaye Bog	Hautes-Fagnes	Belgium	50.5	6.03	Allan et al. 2018 (157)	https://doi.org/10.3390/atmos9070253

P11	30	Peat core	Misten Bog	Hautes-Fagnes	Belgium	50.5	6.03	Allan et al. 2018 (157); De Vleeschouwer et al. 2007 (158)	https://doi.org/10.3390/atmos9070253 ; https://doi.org/10.1016/j.scitotenv.2007.02.017
P12	32	Peat core	Lindow Bog	Manchester	UK	53.323	-2.269	Le Roux et al. 2004 (93)	https://doi.org/10.1039/B401500B
P13	13	Peat core	Myrarnar	Streymoy	Faroe Islands	62.161	-7.089	Shotyk et al. 2005 (159)	https://doi.org/10.1016/j.gca.2004.06.011
P14	4	Peat core	Bog 2	Southern Norway	Norway	58.95	8.9	Dunlap et al. 1999 (160)	https://doi.org/10.1016/S0012-821X(99)00020-5
P15	3	Peat core	Bog 3	Southern Norway	Norway	58.417	6.8	Dunlap et al. 1999 (160)	https://doi.org/10.1016/S0012-821X(99)00020-5
P16	4	Peat core	Bog 4	Western Norway	Norway	61.317	5.5	Dunlap et al. 1999 (160)	https://doi.org/10.1016/S0012-821X(99)00020-5
P17	3	Peat core	Bog 7	Western Norway	Norway	64.117	10.5	Dunlap et al. 1999 (160)	https://doi.org/10.1016/S0012-821X(99)00020-5
P18	100	Peat core	Penido Vello	Xistral Mountains	Spain	43.47	-7.57	Kylander et al. 2005 (20)	https://doi.org/10.1016/j.epsl.2005.09.024
P19	17	Peat core	CR1 & CR2	Hautes-Fagnes	Belgium	50.52	6.08	Renson et al. 2008 (161)	https://doi.org/10.1016/j.apgeochem.2008.06.010

Table S2. Radiocarbon dating results for the Dig1ter peat core. The table reports sample depths, dated materials, laboratory codes, measured radiocarbon ages (BP), associated uncertainties, and calibrated calendar-age ranges (cal BCE/CE; 2σ) used to construct the Dig1ter age–depth model.

Sample	Lab code	Depth (cm)	Measured Age (BP)	Calibrated Age (2σ)	Status	Dated material
Dig1Ter10	POZ-163056	12–13	1105 ± 30	885–996 CE	rejected (too old)	Bulk peat
Dig1Ter8	Ly-20071 (SacA-69647)	19.5–20.5	1170 ± 30	772–974 CE	accepted	Small wood fragments (*Alnus* and *Betula*) + *Eriophorum* spines
Dig1Ter2	POZ-162728	34.5–35.5	2885 ± 30	1131–977 BCE	accepted	Small wood fragments (*Alnus* and *Betula*) + *Eriophorum* spines
Dig1Ter9	Ly-20072 (SacA-69648)	49.5–50.5	2930 ± 30	1222–1016 BCE	accepted	Small wood fragments (*Alnus* and *Betula*) + *Eriophorum* spines
Dig1Ter4	Ly-20410 (SacA-70510)	84.5–85.5	4105 ± 30	2866–2503 BCE	accepted	Small wood fragments (*Salix* and *Betula*)
Dig1Ter6	Ly-20069 (SacA-69645)	134.5–135.5	4905 ± 30	3766–3637 BCE	accepted	Small wood fragments (indet.)
Dig1Ter7	Ly-20070 (SacA-69646)	159.5–160.5	5355 ± 30	4326–4054 BCE	accepted	Small wood fragments (*Betula*)

Table S3. Radionuclide activity measurements for the Dig1ter peat core. The table reports sample depth intervals and activities of excess ^{210}Pb, ^{137}Cs, and ^{241}Am (Bq kg^{-1}), together with their associated uncertainties. These measurements were used to establish the recent chronology of the upper part of the core and to complement the radiocarbon-based age–depth model.

Depth average (cm)	Depth min (cm)	Depth max (cm)	^{210}Pbex	^{210}Pbex_error	^{137}Cs	^{137}Cs error	^{241}Am	^{241}Am error
1	0.5	1.5	504.3682131	8.334781174	96.36110186	1.360183833	0	0.618182682
2	1.5	2.5	557.767131	11.58948363	90.78485757	1.744422612	0	0.772206365
3	2.5	3.5	409.8597761	9.561950477	86.0048639	1.605018453	1.405820655	0.274495258
4	3.5	4.5	258.7952369	5.531825633	88.86258293	1.149855401	2.771967492	0.230068128
5	4.5	5.5	152.5791281	5.262974589	48.86174286	1.026111283	3.724412745	0.282676842
6	5.5	6.5	85.12396842	6.032827508	37.97386933	1.282027098	2.161476771	0.350068462
7	6.5	7.5	43.64075981	4.428847706	30.26477984	1.099385533	1.167639885	0.247646205
8	7.5	8.5	31.58719328	4.545218747	21.94397281	1.035221706	0	0.84011689
9	8.5	9.5	30.83288068	4.548017943	22.50729279	1.040063894	1.243620564	0.3034697
10	9.5	10.5	11.51007488	3.241386203	16.42357415	0.854999836	0	0.867125811
11	10.5	11.5	9.980244594	2.954120522	9.529450705	0.683444344	0	0.71300403
12	11.5	12.5	6.270231131	2.254978275	6.682461698	0.466385195	0	0.60605993
13	12.5	13.5	8.789717672	2.661877647	4.566187622	0.463588479	0	0.644219031
15	14.5	15.5	0	5.565692273	3.403468169	0.456105164	0	0.58528554

Table S4. Geochemical and Pb isotope data for the Dig1ter peat core. The table reports sample depth intervals, calibrated ages derived from the age–depth model, major- and trace-element concentrations (ppm), Pb isotope ratios ($^{206}Pb/^{204}Pb$, $^{207}Pb/^{204}Pb$, $^{208}Pb/^{204}Pb$, $^{207}Pb/^{206}Pb$, and $^{208}Pb/^{206}Pb$) with associated 2σ uncertainties, and the derived geochemical parameters T_{mod}, μ, and κ for each sample.

				Major and trace elements concentrations (ppm)																
Sample	Depth min	Depth max	Age model	Na	Mg	Al	Fe	Ti	V	Cr	Mn	Co	Ni	Cu	Ge	As	Mo	Cd	Sb	Te
Di1	0	1	2022	149.66	708.98	2781.17	2732.72	89.79	5.73	4.24	75.13	1.32	3.11	9.30	0.04	3.93	2.38	0.85	1.12	0.59
Di2	1	2	2015	144.75	788.19	2679.30	3356.78	80.81	6.42	4.32	62.49	1.94	2.91	7.47	1.31	1.94	18.14	1.59	1.80	0.88
Di3	2	3	2008	144.37	609.21	2475.13	4249.76	77.58	6.35	6.70	45.19	1.29	7.16	6.68	1.08	4.37	6.53	1.16	1.73	0.59
Di4	3	4	1994	144.54	682.78	3804.38	9679.71	139.27	10.06	9.61	48.36	1.79	6.69	6.98	4.32	9.05	12.21	1.60	2.14	1.40
Di5	4	5	1974	150.55	570.99	4454.15	8655.66	188.27	7.78	7.54	28.96	1.43	3.66	4.12	1.15	16.39	4.40	1.68	2.43	NA
Di6	5	6	1961	151.09	513.47	4890.69	5653.92	213.14	6.61	6.62	19.12	1.00	3.00	1.08	0.79	12.85	4.08	0.99	2.27	1.08
Di7	6	7	1949	132.72	432.08	4253.33	7025.05	184.88	5.82	5.14	14.03	0.75	2.03	3.03	0.01	12.14	1.56	0.55	1.03	0.75
Di8	7	8	1933	117.98	403.43	5116.04	3399.41	193.18	5.85	5.22	11.46	0.70	2.62	1.74	0.04	5.58	1.09	0.53	0.83	0.24
Di9	8	9	1917	119.32	534.95	7186.24	1697.14	205.66	7.59	6.79	10.69	0.78	0.78	NA	NA	2.60	0.96	0.38	0.60	1.37
Di10	9	10	1901	116.45	391.05	6025.44	1106.23	211.44	6.96	6.71	8.33	0.73	2.68	24.88	0.02	2.35	0.45	0.36	0.37	0.68
Di11	10	11	1896	104.48	331.92	4923.87	1129.33	135.09	4.91	3.95	5.44	0.66	1.25	1.13	0.89	1.27	1.22	0.48	1.14	1.27
Di12	11	12	1881	108.38	365.38	5158.44	1193.72	143.93	5.60	5.31	4.30	0.70	1.87	2.37	0.03	1.20	0.32	0.37	0.19	0.52
Di13	12	13	1868	109.74	373.30	5573.09	1009.06	145.74	5.28	4.56	5.04	0.65	1.68	NA	0.33	0.62	0.70	0.32	0.15	0.03
Di14	13	14	1854	94.37	357.27	4513.03	1350.97	133.10	4.88	4.23	3.60	0.53	1.21	1.87	0.05	1.88	0.11	0.18	0.09	0.35
Di15	14	15	1841	89.93	305.29	4112.19	1228.01	136.83	4.50	3.98	5.13	0.51	1.11	1.45	NA	1.38	0.29	0.23	0.19	0.31
Di16	15	16	1827	192.94	418.98	5201.35	1186.39	127.89	5.01	3.55	5.96	0.55	1.89	NA	0.16	0.47	0.50	0.25	NA	0.46
Di18	17	18	1319	111.83	351.55	4606.56	1139.00	145.64	5.69	4.60	3.66	0.64	1.08	1.70	0.05	1.66	0.33	0.12	0.26	0.31
Di19	18	19	1054	105.31	345.71	4268.69	971.79	123.35	5.10	4.75	3.49	0.62	1.57	1.87	0.06	0.87	0.33	0.10	0.14	0.31
Di20	19	20	790	92.02	492.71	5382.63	1439.68	123.23	6.05	5.63	9.46	0.84	2.41	0.04	2.66	1.41	8.51	0.47	0.54	1.42
Di22	21	22	419	166.10	594.87	3537.46	1885.98	79.13	5.70	3.15	12.04	1.43	4.04	1.55	0.21	0.70	0.91	0.21	6.25	NA
Di24	23	24	205	195.56	685.67	3765.28	2106.66	86.17	8.33	4.16	16.59	1.37	2.83	1.55	0.56	0.26	1.18	0.12	1.08	2.63
Di25	24	25	99	130.72	595.32	3299.54	2635.58	76.04	8.68	3.96	15.10	1.34	1.90	3.42	0.03	1.27	0.75	0.19	0.09	0.26
Di26	25	26	-8	163.33	694.68	3038.39	3019.05	73.12	10.42	3.53	18.93	1.44	1.45	1.37	0.46	0.75	1.04	0.19	0.40	0.41
Di28	27	28	-276	195.23	849.61	4589.92	4981.80	119.33	13.34	4.56	26.97	1.46	2.73	1.22	1.01	0.90	1.23	0.33	0.27	0.00
Di31	30	31	-677	159.64	987.04	3963.23	5545.00	96.42	18.06	4.19	27.41	1.58	3.29	0.58	0.53	0.98	1.31	0.06	NA	0.02
Di33	32	33	-752	199.85	888.51	4401.81	5092.83	116.00	17.12	3.84	33.07	1.80	3.38	0.72	0.82	0.58	1.97	0.00	NA	NA
Di34	33	34	-790	163.57	878.33	3698.91	4937.48	117.35	16.99	4.54	36.13	2.15	2.78	2.90	NA	2.47	1.71	0.18	0.10	0.68
Di35	34	35	-827	183.44	939.61	3497.78	6057.50	110.47	14.24	4.51	37.99	2.27	2.87	2.82	NA	2.83	2.11	0.15	0.09	0.86
Di36	35	36	-865	174.00	889.52	3310.32	3683.00	80.83	10.80	3.45	37.38	2.29	1.60	NA	1.12	1.06	1.30	0.06	0.06	0.49
Di38	37	38	-888	157.07	834.79	3366.84	3479.96	107.49	18.42	4.33	34.72	2.07	2.73	2.43	0.05	1.68	1.83	0.09	0.11	0.30
Di39	38	39	-899	169.47	970.33	4839.19	3810.52	169.05	22.32	5.67	42.52	2.23	3.47	3.26	0.04	1.60	2.31	0.19	0.14	0.57
Di41	40	41	-922	176.67	934.40	2181.43	2178.47	64.88	8.64	2.29	50.22	2.40	2.81	NA	0.31	0.27	1.55	0.35	0.22	1.32
Di43	42	43	-942	148.47	927.43	4257.28	3030.48	162.47	22.57	4.87	39.78	1.93	2.95	2.47	0.06	1.91	2.75	0.13	0.10	0.51
Di46	45	46	-973	212.88	1136.38	3635.31	3921.15	96.07	11.46	3.32	56.10	1.96	3.36	0.27	0.61	0.90	1.95	0.05	2.29	NA
Di48	47	48	-998	144.78	869.60	2402.98	2975.14	69.34	8.02	2.59	48.69	1.99	2.28	1.67	0.02	1.96	2.31	0.14	0.08	0.51
Di49	48	49	-1011	135.56	1064.79	2955.14	3093.73	81.26	17.65	3.06	51.43	1.69	1.83	NA	2.51	0.21	9.89	0.13	0.41	1.69
Di51	50	51	-1036	169.99	991.53	3258.92	2345.23	89.17	13.19	3.33	52.98	2.00	3.20	NA	0.15	1.61	2.11	0.05	NA	NA
Di56	55	56	-1192	216.83	1085.44	3325.30	2383.61	90.73	9.76	2.81	59.90	2.31	3.13	NA	0.94	1.77	1.86	0.29	0.13	NA
Di61	60	61	-1362	260.92	1540.11	4233.73	4854.43	206.81	32.61	5.98	69.93	2.64	5.83	0.87	0.85	1.43	4.00	0.11	0.18	NA
Di65	64	65	-1485	166.30	1101.94	3014.79	2593.43	101.79	7.88	3.88	64.83	2.69	2.69	NA	NA	0.93	1.24	NA	0.00	1.53
Di76	75	76	-1857	150.82	976.58	2472.85	2371.92	73.22	10.34	3.19	64.33	1.56	3.19	NA	0.00	0.38	1.07	0.36	NA	0.01
Di79	78	79	-1958	118.99	878.48	1958.95	2109.62	71.80	4.31	2.31	59.13	1.88	1.69	NA	1.91	0.25	5.56	0.10	0.11	0.40
Di86	85	86	-2167	257.03	1639.74	4841.44	3498.29	311.44	18.33	7.15	90.27	2.69	6.00	3.73	0.17	1.32	1.45	0.21	0.34	NA
Di99	98	99	-2385	143.22	968.45	3093.58	1771.94	106.11	8.18	3.22	53.78	1.75	2.24	NA	2.88	NA	5.18	0.14	0.35	1.53
Di121	120	121	-2746	212.93	1071.97	2442.80	1712.97	70.61	24.01	4.46	65.81	2.27	4.04	0.71	NA	1.15	2.69	0.76	0.21	NA
Di141	140	141	-3083	224.86	1111.22	3730.39	2254.41	260.51	32.24	6.38	77.01	1.93	5.59	4.10	0.29	2.79	2.11	0.64	0.27	NA
Di159	158	159	-3400	157.65	1232.61	12151.05	2390.61	433.26	29.94	29.94	76.55	1.39	1.39	8.10	NA	5.79	2.23	0.00	0.01	1.56
Di167	166	167	-3737	192.17	1281.59	13877.07	2923.77	638.84	41.88	21.78	79.67	1.36	10.77	7.03	1.06	7.54	2.56	0.33	0.11	NA
Di171	170	171	-3940	170.96	1629.86	21890.15	4112.67	970.79	61.06	23.36	75.92	1.86	12.70	5.84	3.94	5.55	5.09	0.70	0.09	0.11
Di175	174	175	-4145	166.39	1997.45	20354.27	5133.55	1177.53	34.72	18.83	78.89	1.72	11.19	2.00	8.67	2.51	1.12	0.16	NA	2.36
Di186	185	186	-4703	173.74	2082.74	17506.85	5292.70	1098.42	26.42	14.96	86.08	2.05	10.41	0.69	3.15	1.41	0.53	0.16	NA	0.44
Di197	196	197	-5264	106.92	2810.23	32639.02	7905.88	1191.02	32.68	23.54	94.75	2.75	12.72	1.94	4.02	2.66	3.47	0.00	0.07	0.71
Di199	198	199	-5367	197.54	4693.24	55126.49	13936.82	1898.70	62.68	45.49	153.95	5.20	23.50	4.91	7.27	6.06	3.39	0.13	0.00	0.67

									Isotopic ratios Pb												
Te	Ba	Ce	Pr	Nd	Gd	Dy	Yb	Pb	206Pb/204Pb	2 sigma	207Pb/204Pb	2 sigma	208Pb/204Pb	2 sigma	207Pb/206Pb	2 sigma	208Pb/206Pb	2 sigma	T	µ	k
0.59	43.13	1.84	4.77	15.29	0.38	0.15	0.09	42.47	18.0406	0.0006	15.6123	0.0005	38.018	0.001	0.86500	0.00001	2.10735	0.00006	482.01	9.70	3.94
0.88	42.42	4.48	5.24	16.32	0.20	0.22	0.03	55.82	17.9706	0.0006	15.6060	0.0005	37.943	0.002	0.86840	0.00001	2.11132	0.00005	523.45	9.70	3.94
0.59	31.90	4.20	5.93	5.81	0.32	0.11	0.04	75.74	17.9657	0.0006	15.6032	0.0006	37.934	0.002	0.86850	0.00001	2.11152	0.00008	523.66	9.70	3.94
1.40	36.59	11.53	10.14	39.30	0.55	0.36	0.13	100.85	17.9113	0.0007	15.5961	0.0005	37.884	0.002	0.87076	0.00002	2.11503	0.00004	553.41	9.69	3.94
NA	32.36	8.36	9.64	11.04	0.66	0.35	0.09	88.42	18.2072	0.0007	15.6222	0.0006	38.224	0.002	0.85803	0.00001	2.09941	0.00006	377.29	9.70	3.94
1.08	27.05	10.31	13.39	13.53	0.69	0.31	0.13	68.70	18.2864	0.0006	15.6293	0.0006	38.321	0.002	0.85470	0.00001	2.09565	0.00004	330.04	9.71	3.94
0.75	27.18	4.99	13.35	43.53	0.85	0.40	0.14	48.93	18.3446	0.0009	15.6364	0.0008	38.399	0.002	0.85200	0.00001	2.09323	0.00003	297.54	9.71	3.94
0.24	27.54	5.46	14.48	45.77	1.03	0.50	0.12	45.42	18.3859	0.0007	15.6377	0.0006	38.430	0.002	0.85011	0.00001	2.09018	0.00006	269.94	9.71	3.94
1.37	30.72	4.64	4.39	NA	0.53	0.25	NA	39.12	18.4357	0.0004	15.6447	0.0004	38.491	0.001	0.84860	0.00001	2.08785	0.00003	243.19	9.72	3.94
0.68	32.92	7.47	19.36	59.27	1.17	0.66	0.20	29.79	18.4512	0.0005	15.6432	0.0005	38.502	0.001	0.84740	0.00001	2.08671	0.00004	230.47	9.72	3.93
1.27	25.38	11.23	13.09	15.09	0.95	0.45	0.14	22.17	18.4536	0.0007	15.6448	0.0006	38.509	0.002	0.84779	0.00001	2.08679	0.00003	230.70	9.72	3.93
0.52	28.14	4.54	12.19	41.32	0.73	0.44	0.17	27.82	18.4478	0.0005	15.6458	0.0005	38.513	0.002	0.84768	0.00001	2.08763	0.00003	236.02	9.72	3.94
0.03	23.07	8.57	9.45	10.96	0.60	0.42	0.11	20.75	18.4513	0.0006	15.6478	0.0006	38.519	0.001	0.84805	0.00001	2.08762	0.00002	235.85	9.72	3.94
0.35	23.82	3.95	10.22	32.23	0.65	0.42	0.13	21.54	18.4453	0.0006	15.6460	0.0005	38.518	0.002	0.84779	0.00001	2.08824	0.00002	237.92	9.72	3.94
0.31	20.06	3.37	8.78	25.56	0.54	0.32	0.09	16.49	18.4496	0.0006	15.6483	0.0005	38.527	0.001	0.84772	0.00001	2.08821	0.00002	237.66	9.73	3.95
0.46	22.90	6.71	7.69	28.40	0.57	0.28	0.07	16.92	18.4587	0.0006	15.6509	0.0006	38.540	0.001	0.84787	0.00001	2.08786	0.00002	234.33	9.73	3.95
0.31	24.80	4.12	10.12	32.81	0.61	0.34	0.12	13.56	18.4609	0.0007	15.6483	0.0010	38.548	0.001	0.84718	0.00001	2.08808	0.00002	229.73	9.72	3.95
0.31	28.01	3.92	9.66	29.28	0.61	0.33	0.10	11.95	18.4653	0.0006	15.6535	0.0005	38.563	0.001	0.84730	0.00001	2.08841	0.00002	232.88	9.73	3.95
1.42	36.75	11.23	10.95	41.49	0.71	0.41	0.11	11.35	18.4607	0.0007	15.6490	0.0006	38.547	0.002	0.84769	0.00001	2.08800	0.00005	230.69	9.73	3.95
NA	41.03	4.45	5.36	7.58	0.47	0.17	0.12	12.04	18.4876	0.0008	15.6571	0.0007	38.590	0.002	0.84690	0.00001	2.08735	0.00003	221.45	9.74	3.95
2.63	48.68	3.54	4.53	5.07	0.27	0.17	0.12	3.83	18.5081	0.0008	15.6521	0.0007	38.583	0.002	0.84569	0.00001	2.08468	0.00004	200.91	9.73	3.94
0.26	47.09	1.71	4.64	15.67	0.42	0.24	0.12	3.85	18.5088	0.0016	15.6430	0.0014	38.564	0.003	0.84475	0.00001	2.08352	0.00003	189.39	9.71	3.93
0.41	42.46	2.64	3.43	3.86	0.27	0.15	0.08	1.72	18.5719	0.0009	15.6567	0.0009	38.620	0.002	0.84303	0.00002	2.07952	0.00004	161.22	9.73	3.92
0.00	50.42	3.94	5.82	5.58	0.36	0.19	0.07	2.49	18.5864	0.0010	15.6612	0.0009	38.621	0.002	0.84262	0.00001	2.07788	0.00004	156.39	9.74	3.91
0.02	40.45	3.22	3.82	4.53	0.30	0.20	0.08	2.31	18.5743	0.0008	15.6577	0.0007	38.590	0.002	0.84298	0.00001	2.07758	0.00005	160.66	9.73	3.91
NA	37.96	3.08	3.59	4.34	0.28	0.21	0.11	2.35	18.5778	0.0007	15.6620	0.0006	38.584	0.001	0.84306	0.00001	2.07691	0.00002	163.42	9.74	3.90
0.68	45.24	1.76	4.71	16.55	0.46	0.25	0.14	3.24	18.6142	0.0011	15.6541	0.0009	38.597	0.002	0.84056	0.00001	2.07349	0.00003	127.99	9.72	3.89
0.86	42.52	1.47	4.03	14.19	0.39	0.23	0.11	3.21	18.6196	0.0011	15.6538	0.0009	38.600	0.004	0.84029	0.00001	2.07306	0.00003	123.77	9.72	3.89
0.49	32.81	2.54	2.60	3.15	0.24	0.15	0.01	1.40	18.6243	0.0008	15.6644	0.0007	38.610	0.002	0.84107	0.00001	2.07308	0.00005	133.41	9.74	3.89
0.30	37.23	1.39	3.86	13.97	0.47	0.28	0.14	3.04	18.6303	0.0005	15.6637	0.0004	38.607	0.001	0.84039	0.00001	2.07229	0.00002	128.30	9.74	3.88
0.57	36.87	1.70	4.50	17.48	0.53	0.31	0.14	3.62	18.6701	0.0007	15.6645	0.0006	38.615	0.001	0.83864	0.00001	2.06831	0.00002	100.95	9.73	3.87
1.32	23.53	3.26	3.82	5.31	0.43	0.38	0.24	1.38	18.6744	0.0005	15.6681	0.0005	38.617	0.002	0.83901	0.00001	2.06790	0.00004	102.28	9.74	3.87
0.51	29.82	1.45	3.69	14.25	0.46	0.27	0.13	3.76	18.6581	0.0006	15.6646	0.0005	38.591	0.001	0.84718	0.00001	2.06833	0.00002	109.59	9.74	3.86
NA	29.66	2.74	3.69	3.89	0.32	0.23	0.13	1.99	18.5923	0.0007	15.6586	0.0006	38.572	0.002	0.84221	0.00001	2.07462	0.00004	149.05	9.73	3.89
0.51	28.87	1.24	3.69	16.07	0.45	0.38	0.26	2.61	18.5196	0.0004	15.6513	0.0004	38.523	0.001	0.84475	0.00001	2.08013	0.00001	191.75	9.72	3.91
1.69	31.47	4.72	5.61	24.57	0.73	0.53	0.24	0.02													
NA	26.76	3.23	3.73	4.89	0.37	0.33	0.16	1.53	18.6758	0.0007	15.6682	0.0005	38.645	0.002	0.83896	0.00001	2.06924	0.00002	101.37	9.74	3.88
NA	35.60	3.55	4.41	5.16	0.48	0.38	0.19	1.71	18.6816	0.0008	15.6633	0.0007	38.632	0.002	0.83843	0.00001	2.06790	0.00004	91.11	9.73	3.87
NA	55.08	4.48	6.71	6.86	0.49	0.41	0.23	3.07	18.6893	0.0007	15.6605	0.0006	38.602	0.002	0.83794	0.00001	2.06550	0.00004	82.20	9.72	3.85
1.53	55.04	0.85	1.11	NA	0.07	0.02	NA	1.13	18.6799	0.0008	15.6636	0.0007	38.635	0.002	0.83853	0.00001	2.06821	0.00003	92.77	9.73	3.87
0.01	26.97	2.65	3.21	3.64	0.27	0.14	0.11	0.81													
0.40	25.89	2.45	2.71	9.11	0.16	0.18	0.07	0.15													
NA	41.50	2.97	3.58	4.42	0.34	0.26	0.11	2.98	18.7220	0.0007	15.6650	0.0005	38.585	0.001	0.83672	0.00001	2.06094	0.00003	64.33	9.73	3.83
1.53	24.79	1.87	1.19	5.01	0.15	0.17	0.06	0.78	18.6141	0.0009	15.6584	0.0007	38.519	0.002	0.84120	0.00002	2.06930	0.00004	133.29	9.73	3.85
NA	33.12	2.36	3.38	3.74	0.24	0.21	0.09	1.41	18.7164	0.0011	15.6719	0.0010	38.596	0.003	0.83735	0.00001	2.06220	0.00005	77.02	9.74	3.84
NA	38.50	3.74	5.26	5.33	0.45	0.24	0.11	3.72	18.7430	0.0007	15.6734	0.0006	38.582	0.002	0.83623	0.00001	2.05850	0.00004	59.79	9.74	3.82
1.56	33.94	1.87	1.75	NA	0.34	0.17	NA	8.32	18.7234	0.0006	15.6729	0.0006	38.575	0.002	0.83709	0.00001	2.06026	0.00003	73.22	9.75	3.82
NA	45.05	12.09	16.03	18.13	1.31	0.57	0.19	10.94	18.7453	0.0007	15.6706	0.0006	38.584	0.002	0.83597	0.00001	2.05834	0.00006	54.75	9.74	3.82
0.11	74.22	58.51	195.55	NA	2.62	1.78	0.33	13.03													
2.36	85.11	129.44	159.12	180.57	11.91	4.12	0.40	12.32	18.8152	0.0006	15.6773	0.0006	38.678	0.002	0.83322	0.00001	2.05568	0.00005	12.93	9.75	3.82
0.44	79.76	39.06	48.63	52.75	3.40	1.23	0.50	9.77	18.8091	0.0007	15.6744	0.0006	38.679	0.002	0.83334	0.00001	2.05643	0.00006	13.66	9.74	3.82
0.71	83.68	74.33	81.48	269.71	3.87	2.10	0.30	8.83	18.7100	0.0008	15.6762	0.0007	38.669	0.002	0.83784	0.00001	2.06672	0.00004	86.89	9.75	3.87
0.67	140.80	136.03	142.33	493.51	7.40	3.57	0.69	21.31													

REFERENCES

1. E. Ben-Yosef, Archaeological science brightens Mediterranean dark age. *Proc. Natl. Acad. Sci. U.S.A.* **116**, 5843–5845 (2019).

2. D. Killick, T. Fenn, Archaeometallurgy: The study of preindustrial mining and metallurgy. *Ann. Rev. Anthropol.* **41**, 559–575 (2012).

3. C. C. Patterson, Silver stocks and losses in ancient and medieval times. *Econ. Hist. Rev.* **25**, 205–233 (1972).

4. F. Albarède, J. Blichert-Toft, F. de Callataÿ, G. Davis, P. Debernardi, L. Gentelli, H. Gitler, F. Kemmers, S. Klein, C. Malod-Dognin, J. Milot, P. Télouk, M. Vaxevanopoulos, K. Westner, From commodity to money: The rise of silver coinage around the Ancient Mediterranean (sixth–first centuries bce). *Archaeometry* **63**, 142–155 (2021).

5. F. Albarède, G. Davis, H. Gitler, J. Blichert-Toft, L. Gentelli, Levantine Hacksilber and the flow of silver in early Mediterranean commerce. *Archaeometry* **67**, 1547–1564 (2025).

6. G. Davis, J. Blichert-Toft, L. Gentelli, D. B. Gore, K. A. Sheedy, F. Albarède, Identifying silver ore sources for the earliest coins of Athens. *Archaeol. Anthropol. Sci.* **17**, 45 (2025).

7. C. C. Patterson, Contaminated and natural lead environments of man. *Arch. Environ. Health* **11**, 344–360 (1965).

8. C. C. Patterson, H. Shirahata, J. E. Ericson, Lead in ancient human bones and its relevance to historical developments of social problems with lead. *Sci. Total Environ.* **61**, 167–200 (1987).

9. O. López-Costas, M. Kylander, N. Mattielli, N. Álvarez-Fernández, M. Pérez-Rodríguez, T. Mighall, R. Bindler, A. Martínez Cortizas, Human bones tell the story of atmospheric mercury and lead exposure at the edge of Roman World. *Sci. Total Environ.* **710**, 136319 (2020).

10. J. R. McConnell, N. J. Chellman, A. Plach, S. M. Wensman, G. Plunkett, A. Stohl, N.-K. Smith, B. Møllesøe Vinther, D. Dahl-Jensen, J. P. Steffensen, D. Fritzsche, S. O. Camara-Brugger, B. T. McDonald, A. I. Wilson, Pan-European atmospheric lead pollution, enhanced

blood lead levels, and cognitive decline from Roman-era mining and smelting. *Proc. Natl. Acad. Sci. U.S.A.* **122**, e2419630121 (2025).

11. S. C. Gilfillan, Lead poisoning and the fall of Rome. *J. Occup. Med.* **7**, 53–60 (1965).

12. J. O. Nriagu, *Lead and Lead Poisoning in Antiquity* (John Wiley & Sons, 1983).

13. C. P. Loveluck, M. McCormick, N. E. Spaulding, H. Clifford, M. J. Handley, L. Hartman, H. Hoffmann, E. V. Korotkikh, A. V. Kurbatov, A. F. More, S. B. Sneed, P. A. Mayewski, Alpine ice-core evidence for the transformation of the European monetary system, AD 640–670. *Antiquity* **92**, 1571–1585 (2018).

14. J. R. McConnell, A. I. Wilson, A. Stohl, M. M. Arienzo, N. J. Chellman, S. Eckhardt, E. M. Thompson, A. M. Pollard, J. P. Steffensen, Lead pollution recorded in Greenland ice indicates European emissions tracked plagues, wars, and imperial expansion during antiquity. *Proc. Natl. Acad. Sci. U.S.A.* **115**, 5726–5731 (2018).

15. J. R. McConnell, N. J. Chellman, A. I. Wilson, A. Stohl, M. M. Arienzo, S. Eckhardt, D. Fritzsche, S. Kipfstuhl, T. Opel, P. F. Place, J. P. Steffensen, Pervasive Arctic lead pollution suggests substantial growth in medieval silver production modulated by plague, climate, and conflict. *Proc. Natl. Acad. Sci. U.S.A.* **116**, 14910–14915 (2019).

16. W. Shotyk, D. Weiss, P. G. Appleby, A. K. Cheburkin, R. Frei, M. Gloor, J. D. Kramers, S. Reese, W. O. V. D. Knaap, History of atmospheric lead deposition since 12,370 14C yr BP from a Peat Bog, Jura Mountains, Switzerland. *Science* **281**, 1635–1640 (1998).

17. S. Hong, J.-P. Candelone, C. C. Patterson, C. F. Boutron, Greenland ice evidence of hemispheric lead pollution two millennia ago by greek and roman civilizations. *Science* **265**, 1841–1843 (1994).

18. R. Bindler, Comment on "next-generation ice core technology reveals true minimum natural levels of lead (Pb) in the atmosphere: Insights From the black death" by More et al. *GeoHealth* **2**, 155–161 (2018).

19. N. Silva-Sánchez, X.-L. Armada, Environmental impact of roman mining and metallurgy and its correlation with the archaeological evidence: A european perspective. *Environ. Archaeol.* 10.1080/14614103.2023.2181295 (2023).

20. M. Kylander, D. J. Weiss, A. Martineez-Cortizas, B. Sprio, R. Garcia-Sanchez, B. J. Coles, Refining the pre-industrial atmospheric Pb isotope evolution curve in Europe using an 8000 year old peat core from NW Spain. *Earth Planet. Sci. Lett.* **240**, 467–485 (2005).

21. I. Renberg, M. L. Brännvall, R. Bindler, O. Emteryd, Stable lead isotopes and lake sediments—A useful combination for the study of atmospheric lead pollution history. *Sci. Total Environ.* **292**, 45–54 (2002).

22. Z. A. Stos-Gale, N. H. Gale, Metal provenancing using isotopes and the Oxford archaeological lead isotope database (OXALID). *Archaeol. Anthropol. Sci.* **1**, 195–213 (2009).

23. K. J. R. Rosman, W. Chisholm, S. Hong, J.-P. Candelone, C. F. Boutron, Lead from Carthaginian and Roman Spanish mines isotopically identified in Greenland ice dated from 600 B.C. to 300 A.D. *Environ. Sci. Technol.* **31**, 3413–3416 (1997).

24. C. Tomczyk, A database of lead isotopic signatures of copper and lead ores for Europe and the Near East. *J. Archaeol. Sci.* **146**, 105657 (2022).

25. T. Eshel, A. Gilboa, N. Yahalom-Mack, O. Tirosh, Y. Erel, Debasement of silver throughout the Late Bronze – Iron Age transition in the Southern Levant: Analytical and cultural implications. *J. Archaeol. Sci.* **125**, 105268 (2021).

26. K. J. Westner, T. Birch, F. Kemmers, S. Klein, H. E. Höfer, H.-M. Seitz, ROME'S rise to power. Geochemical analysis of silver coinage from the Western Mediterranean (fourth to second centuries BCE). *Archaeometry* **62**, 577–592 (2020).

27. F. Albarede, G. Davis, J. Blichert-Toft, L. Gentelli, H. Gitler, M. Pinto, P. Telouk, A new algorithm for using Pb isotopes to determine the provenance of bullion in ancient Greek coinage. *J. Archaeol. Sci.* **163**, 105919 (2024a).

28. F. Albarede, G. Davis, L. Gentelli, J. Blichert-Toft, H. Gitler, M. Pinto, P. Telouk, Bullion mixtures in silver coinage from ancient Greece and Egypt. *J. Archaeol. Sci.* **162**, 105918 (2024b).

29. R. M. Ellam, The graphical presentation of lead isotope data for environmental source apportionment. *Sci. Total Environ.* **408**, 3490–3492 (2010).

30. J. Zalasiewicz, C. N. Waters, M. Williams, C. P. Summerhayes, Eds., *The Anthropocene as a Geological Time Unit: A Guide to the Scientific Evidence and Current Debate* (Cambridge Univ. Press, Cambridge, 2019).

31. T. Rose, A. Wittke, S. Klein, Comment on "Limitations in using the Cu isotopic composition of minerals from ancient copper mines for archaeometric purposes—A case study" by P. A. Penanes, M. Costas-Rodríguez, M. Moldovan, J. I. García Alonso and F. Vanhaecke, J. Anal. At. Spectrom., 2023, 38, 1611. *J. Anal. At. Spectrom.* **40**, 2599–2603 (2025).

32. J. Longman, D. Veres, V. Ersek, D. L. Phillips, C. Chauvel, C. G. Tamas, Quantitative assessment of Pb sources in isotopic mixtures using a Bayesian mixing model. *Sci. Rep.* **8**, 6154 (2018).

33. S. Klein, T. Rose, K. J. Westner, Y.-K. Hsu, From OXALID to GlobaLID: Introducing a modern and FAIR lead isotope database with an interactive application. *Archaeometry* **64**, 935–950 (2022).

34. S. De Ceuster, J. Hoogewerff, P. Degryse, Provenancing ancient materials with lead isotopes: Overlap uncovered. *Sci. Rep.* **15**, 4628 (2025).

35. J. D. Kramers, I. N. Tolstikhin, Two terrestrial lead isotope paradoxes, forward transport modelling, core formation and the history of the continental crust. *Chem. Geol.* **139**, 75–110 (1997).

36. S. M. Wensman, A. E. Shiel, J. R. McConnell, Lead isotopic fingerprinting of 250-years of industrial era pollution in Greenland ice. *Anthropocene* **38**, 100340 (2022).

37. A. J.-M. Bory, W. Abouchami, S. J. G. Galer, A. Svensson, J. N. Christensen, P. E. Biscaye, A Chinese imprint in insoluble pollutants recently deposited in central Greenland as indicated by lead isotopes. *Environ. Sci. Technol.* **48**, 1451–1457 (2014).

38. F. De Vleeschouwer, S. Baron, J. M. Cloy, M. Enrico, V. Ettler, N. Fagel, H. Kempter, M. Kylander, C. Li, J. Longman, A. Martinez-Cortizas, S. Marx, N. Mattielli, T. Mighall, T. M. Nieminen, N. Piotrowska, X. Pontevedra-Pombal, S. Pratte, V. Renson, W. Shotyk, E. Shuttleworth, J. Sikorski, N. Stromsoe, J. Talbot, C. von Scheffer, D. Weiss, C. Zaccone, G. Le Roux, Comment on: "A novel approach to peatlands as archives of total cumulative spatial pollution loads from atmospheric deposition of airborne elements complementary to EMEP data: Priority pollutants (Pb, Cd, Hg)" by Ewa Miszczak, Sebastian Stefaniak, Adam Michczyński, Eiliv Steinnes and Irena Twardowska. *Sci. Total Environ.* **737**, 138699 (2020).

39. J. Blichert-Toft, H. Delile, C.-T. Lee, Z. Stos-Gale, K. Billström, T. Andersen, H. Hannu, F. Albarede, Large-scale tectonic cycles in Europe revealed by distinct Pb isotope provinces. *Geochem. Geophys. Geosyst.* **17**, 3854–3864 (2016).

40. M. E. Kylander, J. Klaminder, R. Bindler, D. J. Weiss, Natural lead isotope variations in the atmosphere. *Earth Planet. Sci. Lett.* **290**, 44–53 (2010).

41. J. Longman, D. Veres, W. Finsinger, V. Ersek, Exceptionally high levels of lead pollution in the Balkans from the early bronze age to the industrial revolution. *Proc. Natl. Acad. Sci. U.S.A.* **115**, 5661–5668 (2018).

42. A. Martínez Cortizas, L. López-Merino, R. Bindler, T. Mighall, M. E. Kylander, Early atmospheric metal pollution provides evidence for Chalcolithic/Bronze Age mining and metallurgy in Southwestern Europe. *Sci. Total Environ.* **545–546**, 398–406 (2016).

43. E. Andrikou, "The exploitation of the argentiferous ores in the lavreotike peninsula, attica, in antiquity: Some remarks on recent evidence," in *Metallurgy in Numismatics 6 Mines, Metals, and Money Ancient World Studies in Science, Archaeology and History* (Royal Numismatic Society, 2021), pp. 59–66.

44. P. Spitaels, “The Early Helladic period in Mine N°3 (Theatre Sector)” (Rapport Préliminaire sur les 9e, 10e, 11e en 12e Campagnes de Fouilles Thorikos VIII, 1972/1976, Comité des Fouilles Belges en Grèce, 1984).

45. A. Tarantola, C. Scheffer, A. Photiades, O. Vanderhaeghe, D. Morin, “Geologic and metallogenic overview of the Lavrion mining district: A guide for archaeological exploration,” in *Laurion*. *Interdisciplinary Approaches to an Ancient Greek Mining Landscape: Vol. Der Anschnitt, Beiheft 50 (= Veröffentlichungen aus dem Deutschen Bergbau-Museum Bochum, Nr. 255)*, F. Hulek, H. Lohmann, S. Nomicos, A. Hauptmann, Eds. (Deutsches Bergbau-Museum Bochum - Forschung, 2023), pp. 11–25; https://omp.ub.rub.de/index.php/DBM/catalog/book/264/chapter/388.

46. M. Nazou, “The prehistoric finds from Mine 3 and their relationship to the thorikos mining community,” in *Laurion*. *Interdisciplinary Approaches to an Ancient Greek Mining Landscape: Vol. Der Anschnitt, Beiheft 50 (= Veröffentlichungen aus dem Deutschen Bergbau-Museum Bochum, Nr. 255)*, F. Hulek, H. Lohmann, S. Nomicos, A. Hauptmann, Eds. (Deutsches Bergbau-Museum Bochum - Forschung, 2023), pp. 105–118; https://omp.ub.rub.de/index.php/DBM/catalog/book/264/chapter/396.

47. M. Vaxevanopoulos, J. Ross, K. Soukis, P. Voudouris, V. Melfos, “What did the ancient Greeks mine at Laurion and when did they mine it?,” in *Interdisciplinary Approaches to an Ancient Greek Mining Landscape: Vol. Der Anschnitt, Beiheft 50 (= Veröffentlichungen aus dem Deutschen Bergbau-Museum Bochum, Nr. 255)*, F. Hulek, H. Lohmann, S. Nomicos, A. Hauptmann, Eds. (Deutsches Bergbau-Museum Bochum - Forschung, 2023), pp. 27–45; https://omp.ub.rub.de/index.php/DBM/catalog/book/264/chapter/389.

48. O. A. Kern, A. Koutsodendris, F. Süfke, M. Gutjahr, B. Mächtle, J. Pross, Persistent, multi-sourced lead contamination in Central Europe since the Bronze Age recorded in the Füramoos peat bog, Germany. *Anthropocene* **36**, 100310 (2021).

49. J. L. Fitton, *Cycladic Art* (British Museum, Royaume-Uni de Grande-Bretagne et d’Irlande du Nord, ed. 2, 1999).

50. Z. Stos-Gale, G. Davis, “The minting/mining nexus: new understandings of archaic greek silver coinage from lead isotope analysis,” in *Mines, Metals, and Money Ancient World Studies in Science, Archaeology and History* (Royal Numismatic Society, 2021), pp. 87–100.

51. T. Eshel, Y. Erel, N. Yahalom-Mack, O. Tirosh, A. Gilboa, Lead isotopes in silver reveal earliest Phoenician quest for metals in the west Mediterranean. *Proc. Natl. Acad. Sci. U.S.A.* **116**, 6007–6012 (2019).

52. J.-P. Bravard, *Le Couloir Saône-Rhône: Géohistoire Des Liens Entre Des Montagnes Et Un Axe Fluvial* (L’Harmattan, 2025).

53. C. Joncheray, Les colonies grecques du sud de la Gaule (VII – II s. avant n. è.). *Cad. LEPAARQ* **15**, 401–417 (2018).

54. M. Py, Un bilan sur la circulation monétaire préaugustéenne à Lattes. *Gallia* **65**, 169–174 (2008).

55. K. Gruel, E. Hiriart, “Faciès métalliques des émissions monétaires gauloises de l’âge du Fer,” in *Mines et Métallurgies Anciennes. Mélanges En l’honneur de Béatrice Cauuet* (Ausonius Éditions, 2023), pp. 109–118; https://hal.science/hal-04274660.

56. J. C. Edmondson, Mining in the later roman empire and beyond: Continuity or disruption? *J. Rom. Stud.* **79**, 84–102 (1989).

57. A. Bowman, A. Wilson, “Quantifying the roman economy: Integration, growth, decline?,” in *Quantifying the Roman Economy: Methods and Problems*, A. Bowman, A. Wilson, Eds. (Oxford Univ. Press, 2009), pp. 3–84.

58. C. Howgego, The supply and use of money in the roman world 200 B.C. to A.D. 300. *J. Rom. Stud.* **82**, 1–31 (1992).

59. E. Giannichedda, Metal production in late antiquity: From continuity of knowledge to changes in consumption. *Late Antiq. Archaeol.* **4**, 187–209 (2008).

60. A. F. More, N. E. Spaulding, P. Bohleber, M. J. Handley, H. Hoffmann, E. V. Korotkikh, A. V. Kurbatov, C. P. Loveluck, S. B. Sneed, M. McCormick, P. A. Mayewski, Next-generation ice core technology reveals true minimum natural levels of lead (Pb) in the atmosphere: Insights from the Black Death. *GeoHealth* **1**, 211–219 (2017).

61. C. Domergue, *Les Mines de la Péninsule Ibérique dans l'Antiquité Romaine* (Collection de l'Ecole Française de Rome, École Française de Rome, 1990).

62. C. Domergue, *Les Mines Antiques: La Production des Métaux aux Époques Grecque et Romaine* (Picard, 2008).

63. C. Domergue, C. Rico, Les itinéraires du commerce du cuivre et du plomb hispaniques à l'époque romaine dans le monde méditerranéen. *Bull. Soc. Sci. Hist. Nat. Corse* **746–747**, 135–168 (2014).

64. C. Domergue, C. Rico, *Lateres Plumbei Hispani: Production et Commerce du Plomb Hispanique à l'Epoque Romaine (IIe siècle av.J.-C. - Ier siècle apr. J.-C.)* (Casa de Velázquez, 2023).

65. A. Hirt, "Gold and silver mining in the Roman Empire," in *Debasement: Manipulation of Coin Standards in Pre-Modern Monetary Systems*, K. Butcher, Ed. (Oxbow Books, 2020), pp. 111–124.

66. D. Mladenović, Roman gold and silver mining in the Central Balkans and its significance for the roman state. *Jahrb. Dtsch. Archaol. Inst.* **140**, 1–92 (2025).

67. R. M. Trigo, T. J. Osborn, J. M. Corte-Real, The North Atlantic Oscillation influence on Europe: Climate impacts and associated physical mechanisms. *Climate Res.* **20**, 9–17 (2002).

68. A. Mauri, B. a. S. Davis, P. M. Collins, J. O. Kaplan, The influence of atmospheric circulation on the mid-Holocene climate of Europe: A data–model comparison. *Clim. Past* **10**, 1925–1938 (2014).

69. R. F. Tylecote, *A History of Metallurgy* (Institute of Materials, 1992).

70. L. Long, C. Domergue, Le « véritable plomb de L. Flavivs Vervcla » et autres lingots. L'épave 1 des Saintes-Maries-de-la-Mer. *Mélanges Ec. Fr. Rome* **107**, 801–867 (1995).

71. P. R. Trincherini, P. Barbero, P. Quarati, C. Domergue, L. Long, Where do the lead ingots of the Saintes-Maries-De-La-Mer wreck come from? Archaeology compared with physics. *Archaeometry* **43**, 393–406 (2001).

72. P. Rothenhöfer, "Plumbum Germanicum. Germanic lead and the history of Augustean Germany," in *Archaeometallurgy in Europe, International Conference* (Associazione Italiana di Metalurgia, 2003), vol. 2, pp. 641–648.

73. M. Bode, A. Hauptmann, K. Mezger, Tracing Roman lead sources using lead isotope analyses in conjunction with archaeological and epigraphic evidence—A case study from Augustan/Tiberian Germania. *Archaeol. Anthropol. Sci.* **1**, 177–194 (2009).

74. I. Angelini, C. Canovaro, M. Venturino, G. Artioli, The silver treasure of Marengo: Silver provenancing and insights into late antiquity Roman and Gallo-Roman hoards. *Archaeol. Anthropol. Sci.* **11**, 4959–4970 (2019).

75. K. Butcher, M. Ponting, *The Metallurgy of Roman Silver Coinage: From the Reform of Nero to the Reform of Trajan* (Cambridge Univ. Press, 2015).

76. F. Albarede, M. Boni, J. Blichert-Toft, M. Vaxevanopoulos, K. Westner, J. Milot, M. Pinto, P. Télouk, Ag and Pb isotope systematics in galena ores from southern Sardinia and southern France flag potential silver sources in antiquity. *Archaeol. Anthropol. Sci.* **16**, 17 (2024).

77. S. Baron, M. Lavoie, A. Ploquin, J. Carignan, M. Pulido, J.-L. De Beaulieu, Record of metal workshops in peat deposits: History and environmental impact on the Mont Lozère massif, France. *Environ. Sci. Technol.* **39**, 5131–5140 (2005).

78. M. Besnier, Le commerce du plomb à l'époque romaine d'après les lingots estampillés. *C. R. Séances Acad. Inscr. B.-Lett.* **64**, 18–19 (1920).

79. M. Reddé, Voie maritime ou routes intérieures? La Gaule dans l'organisation du grand commerce vers le nord de l'Empire romain. *Rev. Etud. Anc.* **127**, 73–104 (2025).

80. G. Pagès, P. Dillmann, E. Vega, M. Berranger, S. Bauvais, L. Long, P. Fluzin, Vice-versa: The iron trade in the western Roman Empire between Gaul and the Mediterranean. *PLOS ONE* **17**, e0268209 (2022).

81. M. Bonifay, C. Raynaud, F. Berthault, S. Bien, E. Faure-Boucharlat, D. Foy, T. Mukai, E. Pellegrino, J.-P. Pelletier, D. Pieri, J. Piton, Y. Rigoir, T. Silvino, J.-C. Tréglia, I. Fauduet, I. Odenhardt-Donvez, Échanges et consommation. *Gallia* **64**, 93–161 (2007).

82. W. Scheidel, The shape of the Roman world: Modelling imperial connectivity. *J. Rom. Archaeol.* **27**, 7–32 (2014).

83. M. Flückiger, E. Hornung, M. Larch, M. Ludwig, A. Mees, Roman transport network connectivity and economic integration. *Rev. Econ. Stud.* **89**, 774–810 (2022).

84. T. Franconi, T. Brughmans, E. Borisova, L. Paulsen, From Empire-wide integration to regional localization: A synthetic and quantitative study of heterogeneous amphora data in Roman Germania reveals centuries-long change in regional patterns of production and consumption. *PLOS ONE* **18**, e0279382 (2023).

85. P. Leveau, "The western provinces," in *The Cambridge Economic History of the Greco-Roman World*, I. Morris, R. P. Saller, W. Scheidel, Eds. (Cambridge Univ. Press, 2007), pp. 649–670; www.cambridge.org/core/books/cambridge-economic-history-of-the-grecoroman-world/western-provinces/21222416D70F21093BC83E848562902C.

86. G. Ayala, *Lyon, les Bateaux de Saint-Georges: Une Histoire Sauvée des Eaux* (Éd. Lyonnaises d'Art et d'Histoire Institut National de Recherches Archéologiques Préventives, 2009).

87. S. Marlier, *Arles-Rhône 3, un Chaland Gallo-Romain du Ier Siècle Après Jésus-Christ* (Archaeonautica, CNRS Éditions, 2014), vol. 18; www.cnrseditions.fr/catalogue/revues/archaeonautica-18/.

88. A. Cochet, J. Hansen, *Conduites et Objets de Plomb Gallo-Romains de Vienne (Isère)* (Gallia Supplement, CNRS Éditions, 1986).

89. A. Cochet, *Le plomb en Gaule Romaine: Techniques de Fabrication et Produits* (Editions Monique Mergoil, 2000).

90. F. Adjajd, *Carte Archéologique de La Gaule 38-3: Vienne* (Maison des Sciences de l'Homme, 2014).

91. L. Brissaud, "Les fistulae estampillées VASSEDO. VF: Réflexion critique sur la valeur et la fonction d'un nom," in *Des vestiges aux Sociétés: Regards Croisés sur le Passage des Données Archéologiques à la Société Sous-Jacente*, J. Brancier, C. Rémeaud, T. Vallette, Eds. (Archéo.doct, Éditions de la Sorbonne, 2015), pp. 57–83.

92. C. Heredia, S. Guédron, A. T. Gourlan, B. Helly, H. Delile, L. Calzolari, S. Campillo, S. Santenac, L. Audin, P. Telouk, F. Albarede, Provenance of lead ores used for water pipes production in the ancient Roman Gaul (Vienne, France). *Quat. Sci. Rev.* **353**, 109227 (2025).

93. G. Le Roux, D. Weiss, J. Grattan, N. Givelet, M. Krachler, A. Cheburkin, N. Rausch, B. Kober, W. Shotyk, Identifying the sources and timing of ancient and medieval atmospheric lead pollution in England using a peat profile from Lindow bog, Manchester. *J. Environ. Monit.* **6**, 502–510 (2004).

94. U. Büntgen, V. S. Myglan, F. C. Ljungqvist, M. McCormick, N. D. Cosmo, M. Sigl, J. Jungclaus, S. Wagner, P. J. Krusic, J. Esper, J. O. Kaplan, M. A. C. de Vaan, J. Luterbacher, L. Wacker, W. Tegel, A. V. Kirdyanov, Cooling and societal change during the Late Antique Little Ice Age from 536 to around 660 AD. *Nat. Geosci.* **9**, 231–236 (2016).

95. K. A. F. Zonneveld, K. Harper, A. Klügel, L. Chen, G. De Lange, G. J. M. Versteegh, Climate change, society, and pandemic disease in Roman Italy between 200 BCE and 600 CE. *Sci. Adv.* **10**, eadk1033 (2024).

96. C. Wickham, *Framing the Early Middle Ages: Europe and the Mediterranean, 400-800* (OUP Oxford, 2005).

97. C. Norman, L. Schwinden, P. Krusic, A. Rzepecki, T. Bebchuk, U. Büntgen, Droughts and conflicts during the late Roman period. *Clim. Change* **178**, 87 (2025).

98. C. Norman, L. Schwinden, P. Krusic, A. Rzepecki, T. Bebchuk, U. Büntgen, Response to Foxhall Forbes et al. (2026) “Drought, conflict and the use of historical data and methodologies in interdisciplinary paleoclimatic research”. *Clim. Change* **179**, 74 (2026).

99. H. Foxhall Forbes, M. Fafinski, G. Halsall, J. M. Harland, D. Lawrence, K. Clarke-Neish, L. Foxhall, M. Abballe, M. Borroni, I. Lypiridou, V. Manolopoulou, A. Nikulina, J. Sypiański, D. Fleitmann, Drought, conflict and the use of historical data and methodologies in interdisciplinary palaeoclimatic research. *Clim. Change* **179**, 73 (2026).

100. I. Renberg, R. Bindler, M.-L. Brännvall, Using the historical atmospheric lead-deposition record as a chronological marker in sediment deposits in Europe. *Holocene* **11**, 511–516 (2001).

101. N. J. G. Pounds, *An Economic History of Medieval Europe* (Longman, 1994).

102. C. Wickham, *The Inheritance of Rome: A History of Europe from 400 to 1000* (Penguin Adult, 2010).

103. M.-C. Bailly-Maître, N. Minvielle Larousse, É. Kammenthaler, T. Gonon, G. Guionova, L’exploitation minière dans la vallée du Chassezac (Ardèche). *Arch. Médiév.* **43**, 47–76 (2013).

104. B. M. S. Campbell, *The Great Transition: Climate, Disease and Society in the Late-Medieval World* (Cambridge Univ. Press, Royaume-Uni de Grande-Bretagne et d’Irlande du Nord, 2016).

105. F. Téreygeol, “Y-a-t-il un lien entre la mise en exploitation des mines d’argent de Melle (Deux-Sèvres) et le passage au monométallisme argent vers 675?,” in *Wisigoths et Francs Autour de La Bataille de Vouillé (507). Recherches Récentes Sur Le Haut Moyen Âge Dans Le Centre-Ouest de La France*, L. Bourgeois, Ed. (Saint-Germain-en-Laye, AFAM, 2010), pp. 251–261.

106. F. Téreygeol, How to quantify medieval silver production at Melle. Metalla 20.2, 80–86 (2013).

107. I. Blanchard, *Mining, Metallurgy and Minting in the Middle Ages. Vol. 3, Continuing Afro-European Supremacy, 1250-1450 (African Gold Production and the Second and Third European Silver Production Long-Cycles)* (Franz Steiner Verlag, 2005).

108. S. E. Kelly, N. Brooks, *Charters of Christ Church Canterbury* (The British Academy by Oxford Univ. Press, Royaume-Uni de Grande-Bretagne et d'Irlande du Nord, 2013).

109. H. Kempter, B. Frenzel, The impact of early mining and smelting on the local tropospheric aerosol detected in ombrotrophic peat bogs in the Harz, Germany. *Water Air Soil Pollut.* **121**, 93–108 (2000).

110. J. Longman, D. Veres, V. Ersek, C. G. Tamas, A. Haliuc, E. Magyari, F. Gogaltan, S. Panajiotidis, M. Papadopoulou, Central-Eastern Europe as a centre of Middle Ages extractive metallurgy. *J. Archaeol. Sci.* **172**, 106093 (2024).

111. I. Blanchard, *Mining, Metallurgy and Minting in the Middle Ages. Vol. 1, Asiatic Supremacy, 425-1125* (Franz Steiner Verlag, 2001).

112. S. A. McGuire, M. Hubbe, W. J. Pestle, A. Sharifi, A. Pourmand, Geochemical tales of individual lives in the industrial revolution: Untangling the impact of pollutant exposure in two English towns. *Sci. Adv.* **11**, add1047 (2025).

113. C. Tomczyk, D. Malnar, C. Petit, S. Revillon, L. Costa, From German mines to Le Nôtre's gardens: The origin of the lead in the fountains of Vaux-le-Vicomte and Versailles (Île-de-France, France). *Archaeometry* **68**, 984–993 (2026).

114. M.-L. Bränvall, R. Bindler, O. Emteryd, I. Renberg, Four thousand years of atmospheric lead pollution in northern Europe: A summary from Swedish lake sediments. *J. Paleolimnol.* **25**, 421–435 (2001).

115. I. Renberg, M.-L. Brännvall, R. Bindler, O. Emteryd, Atmospheric lead pollution history during four millennia (2000 BC to 2000 AD) in Sweden. *Ambio* **29**, 150–156 (2000).

116. R. Bindler, Contaminated lead environments of man: reviewing the lead isotopic evidence in sediments, peat, and soils for the temporal and spatial patterns of atmospheric lead pollution in Sweden. *Environ. Geochem. Health* **33**, 311–329 (2011).

117. R. A. Bouchet, "Structure de la lithosphère continentale de l'Ouest USA: Contribution des isotopes du Plomb, du Néodyme, et de l'Hafnium," thesis, Université Claude Bernard Lyon 1, Lyon, École Normale Supérieure (2014).

118. Y.-K. Hsu, B. J. Sabatini, A geochemical characterization of lead ores in China: An isotope database for provenancing archaeological materials. *PLOS ONE* **14**, e0215973 (2019).

119. J. Liebmann, B. Ware, D. R. Mole, C. L. Kirkland, G. Fraser, K. Waltenberg, S. Bodorkos, D. L. Huston, N. J. Evans, B. J. McDonald, K. Rankenburg, P. Datta, S. Tessalina, A crustal Pb isotope map of southeastern Australia. *Sci. Data* **11**, 1222 (2024).

120. F. Albarède, A.-M. Desaulty, J. Blichert-Toft, A geological perspective on the use of Pb isotopes in Archaeometry. *Archaeometry* **54**, 853–867 (2012).

121. C. C. Patterson, Native copper, silver, and gold accessible to early metallurgists. *Am. Antiq.* **36**, 286–321 (1971).

122. D. M. Settle, C. C. Patterson, Lead in albacore: Guide to lead pollution in Americans. *Science* **207**, 1167–1176 (1980).

123. S. Hong, J.-P. Candelone, M. Soutif, C. F. Boutron, A reconstruction of changes in copper production and copper emissions to the atmosphere during the past 7000 years. *Sci. Total Environ.* **188**, 183–193 (1996a).

124. S. Hong, J.-P. Candelone, C. C. Patterson, C. F. Boutron, History of ancient copper smelting pollution during Roman and medieval times recorded in Greenland ice. *Science* **272**, 246–249 (1996b).

125. J. R. McConnell, N. J. Chellman, R. Mulvaney, S. Eckhardt, A. Stohl, G. Plunkett, S. Kipfstuhl, J. Freitag, E. Isaksson, K. E. Gleason, S. O. Brugger, D. B. McWethy, N. J.

Abram, P. Liu, A. J. Aristarain, Hemispheric black carbon increase after the 13th-century Māori arrival in New Zealand. *Nature* **598**, 82–85 (2021).

126. A. I. Wilson, Database of Roman mines, version 3.0, OxREP databases (2025); https://oxrep.web.ox.ac.uk/mines-database [accessed 15 July 2026].

127. J. Strauss, A. I. Wilson, M. Flohr, OxREP Shipwrecks Database, version 2.1 (2017); https://oxrep.web.ox.ac.uk/shipwrecks-database.

128. W. Scheidel, Orbis: The Stanford Geospatial Network Model of the Roman World. Social Science Research Network 2609654 [Preprint] (22 May 2015). https://doi.org/10.2139/ssrn.2609654.

129. L. Stephens, D. Fuller, N. Boivin, T. Rick, N. Gauthier, A. Kay, B. Marwick, C. G. Armstrong, C. M. Barton, T. Denham, K. Douglass, J. Driver, L. Janz, P. Roberts, J. D. Rogers, H. Thakar, M. Altaweel, A. L. Johnson, M. M. S. Vattuone, M. Aldenderfer, S. Archila, G. Artioli, M. T. Bale, T. Beach, F. Borrell, T. Braje, P. I. Buckland, N. G. J. Cano, J. M. Capriles, A. D. Castillo, Ç. Çilingiroğlu, M. N. Cleary, J. Conolly, P. R. Coutros, R. A. Covey, M. Cremaschi, A. Crowther, L. Der, S. di Lernia, J. F. Doershuk, W. E. Doolittle, K. J. Edwards, J. M. Erlandson, D. Evans, A. Fairbairn, P. Faulkner, G. Feinman, R. Fernandes, S. M. Fitzpatrick, R. Fyfe, E. Garcea, S. Goldstein, R. C. Goodman, J. D. Guedes, J. Herrmann, P. Hiscock, P. Hommel, K. A. Horsburgh, C. Hritz, J. W. Ives, A. Junno, J. G. Kahn, B. Kaufman, C. Kearns, T. R. Kidder, F. Lanoë, D. Lawrence, G.-A. Lee, M. J. Levin, H. B. Lindskoug, J. A. López-Sáez, S. Macrae, R. Marchant, J. M. Marston, S. McClure, M. D. McCoy, A. V. Miller, M. Morrison, G. M. Matuzeviciute, J. Müller, A. Nayak, S. Noerwidi, T. M. Peres, C. E. Peterson, L. Proctor, A. R. Randall, S. Renette, G. R. Schug, K. Ryzewski, R. Saini, V. Scheinsohn, P. Schmidt, P. Sebillaud, O. Seitsonen, I. A. Simpson, A. Sołtysiak, R. J. Speakman, R. N. Spengler, M. L. Steffen, M. J. Storozum, K. M. Strickland, J. Thompson, T. L. Thurston, S. Ulm, M. C. Ustunkaya, M. H. Welker, C. West, P. R. Williams, D. K. Wright, N. Wright, M. Zahir, A. Zerboni, E. Beaudoin, S. M. Garcia, J. Powell, A. Thornton, J. O. Kaplan, M.-J. Gaillard, K. K. Goldewijk, E. Ellis, Archaeological assessment reveals Earth's early transformation through land use. *Science* **365**, 897–902 (2019).

130. M. Chenevoy, P. Ledru, J.-L. Feybesse, D. Jauffret, B. Etlicher, *Carte Géologique de la France à 1/50 000, Feuille n 769, Annonay* (BRGM, 1998).

131. H. Cubizolle, P. Bonnel, C. Oberlin, A. Tourman, J. Porteret, Advantages and limits of radiocarbon dating applied to peat inception during the end of the Lateglacial and the Holocene: The example of mires in the Eastern Massif Central (France). *Quaternaire* **18**, 187–208 (2007).

132. J. Argant, H. Cubizolle, L'évolution Holocène de la végétation des monts de la Madeleine, du Forez, du Livradois et du Pilat (Massif Central oriental, France): l'Apport d'une nouvelle série d'analyses palynologiques. *Quaternaire* **16**, 85–94 (2005).

133. P. J. Reimer, W. E. N. Austin, E. Bard, A. Bayliss, P. G. Blackwell, C. B. Ramsey, M. Butzin, H. Cheng, R. L. Edwards, M. Friedrich, P. M. Grootes, T. P. Guilderson, I. Hajdas, T. J. Heaton, A. G. Hogg, K. A. Hughen, B. Kromer, S. W. Manning, R. Muscheler, J. G. Palmer, C. Pearson, J. van der Plicht, R. W. Reimer, D. A. Richards, E. M. Scott, J. R. Southon, C. S. M. Turney, L. Wacker, F. Adolphi, U. Büntgen, M. Capano, S. M. Fahrni, A. Fogtmann-Schulz, R. Friedrich, P. Köhler, S. Kudsk, F. Miyake, J. Olsen, F. Reinig, M. Sakamoto, A. Sookdeo, S. Talamo, The IntCal20 Northern Hemisphere radiocarbon age calibration curve (0–55 cal kBP). *Radiocarbon* **62**, 725–757 (2020).

134. M. Blaauw, J. A. Christen, Flexible paleoclimate age-depth models using an autoregressive gamma process. *Bayesian Anal.* **6**, 457–474 (2011).

135. P. G. Appleby, P. J. Nolan, F. Oldfield, N. Richardson, S. R. Higgitt, ^{210}Pb dating of lake sediments and ombrotrophic peats by gamma essay. *Sci. Total Environ.* **69**, 157–177 (1988).

136. R. Bruel, P. Sabatier, *serac*: An R package for ShortlivEd RAdionuclide chronology of recent sediment cores. *J. Environ. Radioact.* **225**, 106449 (2020).

137. F. Oldfield, P. G. Appleby, R. S. Cambray, J. D. Eakins, K. E. Barber, R. W. Battarbee, G. R. Pearson, J. M. Williams, ^{210}Pb, ^{137}Cs and ^{239}Pu profiles in ombrotrophic peat. *Oikos* **33**, 40–45 (1979).

138. W. Shotyk, H. Kempter, M. Krachler, C. Zaccone, Stable (^{206}Pb, ^{207}Pb, ^{208}Pb) and radioactive (^{210}Pb) lead isotopes in 1 year of growth of *Sphagnum* moss from four ombrotrophic bogs in southern Germany: Geochemical significance and environmental implications. *Geochim. Cosmochim. Acta* **163**, 101–125 (2015).

139. J. C. Ritchie, J. R. McHenry, Application of radioactive fallout cesium-137 for measuring soil erosion and sediment accumulation rates and patterns: A review. *J. Environ. Qual.* **19**, 215–233 (1990).

140. A. Foucher, P.-A. Chaboche, P. Sabatier, O. Evrard, A worldwide meta-analysis (1977–2020) of sediment core dating using fallout radionuclides including ^{137}Cs and $^{210}Pb_{xs}$. *Earth Syst. Sci. Data* **13**, 4951–4966 (2021).

141. P. G. Appleby, W. Shotyk, A. Fankhauser, Lead-210 age dating of three peat cores in the Jura Mountains, Switzerland. *Water Air Soil Poll.* **100**, 223–231 (1997).

142. A. Oleksandrenko, P. G. Appleby, M. J. M. Duke, T. Noernberg, W. Shotyk, Americium-241 in peat bogs as a global marker of the beginning of the Anthropocene: Examples from Europe and North America. *Environ. Rev.* **32**, 707–725 (2024).

143. H. Delile, "Signatures des paléo-pollutions et des paléoenvironnements dans les archives sédimentaires des ports antiques de Rome et d'Éphèse," thesis, Université Lumière Lyon 2, Lyon (2014).

144. H. Delile, J. Blichert-Toft, J.-P. Goiran, S. Keay, F. Albarède, Lead in ancient Rome's city waters. *Proc. Natl. Acad. Sci. U.S.A.* **111**, 6594–6599 (2014).

145. H. Delile, D. Keenan-Jones, J. Blichert-Toft, J.-P. Goiran, F. Arnaud-Godet, P. Romano, F. Albarède, A lead isotope perspective on urban development in ancient Naples. *Proc. Natl. Acad. Sci. U.S.A.* **113**, 6148–6153 (2016).

146. H. Delile, E. Pleuger, J. Blichert-Toft, J.-P. Goiran, N. Fagel, A. Gadhoum, A. Abichou, I. B. Jerbania, E. Fentress, A. I. Wilson, Economic resilience of Carthage during the Punic Wars:

Insights from sediments of the Medjerda delta around Utica (Tunisia). *Proc. Natl. Acad. Sci. U.S.A.* **116**, 9764–9769 (2019).

147. H. Delile, J. Leidwanger, J.-P. Goiran, J. Blichert-Toft, F. Stock, G. Brocard, L. Radloff, E. S. Greene, N. Tuna, Harbor geoarchaeology and urban change in the ancient Knidia (SW Türkiye). *Quat. Sci. Rev.* **369**, 109526 (2025).

148. J. Eisele, W. Abouchami, S. J. G. Galer, A. W. Hofmann, The 320 kyr Pb isotope evolution of Mauna Kea lavas recorded in the HSDP-2 drill core. *Geochem. Geophys. Geosyst.* **4**, 1–32 (2003).

149. F. Monna, J. Dominik, J.-L. Loizeau, M. Pardos, P. Arpagaus, Origin and evolution of Pb in sediments of Lake Geneva (Switzerland−France). Establishing a stable Pb record. *Environ. Sci. Technol.* **33**, 2850–2857 (1999).

150. F. Arnaud, M. Revel-Rolland, D. Bosch, T. Winiarski, M. Desmet, N. Tribovillard, N. Givelet, A 300 year history of lead contamination in northern French Alps reconstructed from distant lake sediment records. *J. Environ. Monit.* **6**, 448–456 (2004).

151. M. Garçon, C. Chauvel, E. Chapron, X. Faïn, M. Lin, S. Campillo, S. Bureau, M. Desmet, M.-C. Bailly-Maître, L. Charlet, Silver and lead in high-altitude lake sediments: Proxies for climate changes and human activities. *Appl. Geochem.* **27**, 760–773 (2012).

152. F. Pasquetti, G. Zanchetta, B. Caron, J. Noel, R. Avanzinelli, B. Vannière, M. Desmet, M. Magny, B. Wagner, L. Dallai, P. Fulignati, M. Bini, I. Baneschi, Lead legacy of pre-industrial activities in lake sediments: The case study of the Lake Accesa (Southern Tuscany, Italy). *Anthropocene* **49**, 100464 (2025).

153. M. P. Griffore, A. E. Shiel, E. C. Rutila, A. L. Hillman, F. Barreiro-Lostres, B. L. Valero-Garcés, M. Morellón, M. B. Abbott, Lead isotope fingerprinting techniques help identify and quantify 3000 years of atmospheric lead pollution from Laguna Roya, northwestern Iberia. *Anthropocene* **42**, 100375 (2023).

154. F. Elbaz-Poulichet, L. Dezileau, R. Freydier, D. Cossa, P. Sabatier, A 3500-year record of Hg and Pb contamination in a Mediterranean sedimentary archive (the Pierre Blanche Lagoon, France). *Environ. Sci. Technol.* **45**, 8642–8647 (2011).

155. D. Weiss, W. Shotyk, P. G. Appleby, J. D. Kramers, A. K. Cheburkin, Atmospheric Pb deposition since the Industrial Revolution recorded by five Swiss peat profiles: Enrichment factors, fluxes, isotopic composition, and sources. *Environ. Sci. Technol.* **33**, 1340–1352 (1999).

156. V. Py, A. Veron, J.-L. Édouard, J.-L. de Beaulieu, B. Ancel, M. Segard, A. Durand, P. Leveau, Interdisciplinary characterisation and environmental imprints of mining and forestry in the upper Durance valley (France) during the Holocene. *Quat. Int.* **353**, 74–97 (2014).

157. M. Allan, D. L. Pinti, B. Ghaleb, S. Verheyden, N. Mattielli, N. Fagel, Reconstruction of atmospheric lead pollution during the Roman period recorded in Belgian ombrotrophic peatlands cores. *Atmos.* **9**, 253 (2018).

158. F. De Vleeschouwer, L. Gérard, C. Goormaghtigh, N. Mattielli, G. Le Roux, N. Fagel, Atmospheric lead and heavy metal pollution records from a Belgian peat bog spanning the last two millenia: Human impact on a regional to global scale. *Sci. Total Environ.* **377**, 282–295 (2007).

159. W. Shotyk, M. E. Goodsite, F. Roos-Barraclough, N. Givelet, G. Le Roux, D. Weiss, A. K. Cheburkin, K. Knudsen, J. Heinemeier, W. O. Van der Knaap, S. A. Norton, C. Lohse, Accumulation rates and predominant atmospheric sources of natural and anthropogenic Hg and Pb on the Faroe Islands. *Geochim. Cosmochim. Acta* **69**, 1–17 (2005).

160. C. E. Dunlap, E. Steinnes, A. R. Flegal, A synthesis of lead isotopes in two millennia of European air. *Earth Planet. Sci. Lett.* **167**, 81–88 (1999).

161. V. Renson, N. Fagel, N. Mattielli, S. Nekrassoff, M. Streel, F. De Vleeschouwer, Roman road pollution assessed by elemental and lead isotope geochemistry in East Belgium. *Appl. Geochem.* **23**, 3253–3266 (2008).
